# A five necroptosis-related lncRNA signature predicts the prognosis of bladder cancer and identifies hot or cold tumors

**DOI:** 10.1097/MD.0000000000035196

**Published:** 2023-10-13

**Authors:** Han Li, Zhengtong Lv, Ming Liu

**Affiliations:** a Department of Urology, Beijing Hospital, National Center of Gerontology, Institute of Geriatric Medicine, Chinese Academy of Medical Sciences, Beijing, China; b Peking University Fifth School of Clinical Medicine, Beijing, China; c Graduate School of Peking Union Medical College and Chinese Academy of Medical Sciences, Beijing, China.

**Keywords:** bladder cancer (BC), cold tumors, hot tumors, immune infiltration, immunotherapy, necroptosis-related lncRNAs, prognosis, risk prediction model, signature

## Abstract

Bladder cancer (BC) is a leading cause of male cancer-related deaths globally. Immunotherapy is showing promise as a treatment option for BC. Numerous studies suggested that necroptosis and long noncoding RNAs (lncRNAs) were critical players in the development of cancers and interacting with cancer immunity. However, the prognostic value of necroptosis-related lncRNAs and their impact on immunotherapeutic response in patients with BC have yet to be well examined. Thus, this study aims to find new biomarkers for predicting prognosis and determining immune subtypes of BC to select appropriate patients from a heterogeneous population. The clinicopathology and transcriptome information from The Cancer Genome Atlas (TCGA) was downloaded, and coexpression analysis was performed to identify necroptosis-related lncRNAs. Then LASSO regression was employed to construct a prediction signature. The signature performance was evaluated by Kaplan–Meier (K–M) method, Time-dependent receiver operating characteristics (ROC). The functional enrichment, immune infiltration, immune checkpoint activation, and the half-maximal inhibitory concentration (IC50) of common drugs in risk groups were compared. The consensus clustering analysis based on lncRNAs associated with necroptosis was made to get 2 clusters to identify hot and cold tumors further. Lastly, the immune response between cold and hot tumors was discussed. In this study, a model containing 5 necroptosis-related lncRNAs was constructed. The risk score distribution of these lncRNAs was compared between low- and high-risk groups in the training, testing, and entire sets. K–M analysis showed that the low-risk patients had significantly better prognosis. The area under the ROC curve (AUC) for the 1-, 3-, and 5-year ROC curves in the entire sets were 0.690, 0.709, and 0.722, respectively. High-risk patients were enriched in lncRNAs related to tumor immunity and had better immune cell infiltration and immune checkpoint activation. Hot tumors and cold tumors were effectively distinguished by clusters 1 and cluster 2, respectively. We developed a necroptosis-related signature based on 5 prognostic lncRNAs, expected to become a new tool for evaluating the prognosis of patients with BC and classifying hot or cold tumors, thus facilitating the development of precision therapy for BC.

## 1. Introduction

BC is the tenth most prevalent cancer worldwide and is ranked ninth concerning death reasons of malignancies in men.^[1]^ Almost all cases of BC originate in the urothelium.^[2]^ Transurethral resection is the primary treatment for BC.^[3]^ With the rapid development of radiomics, more than 30% of patients have found postoperative recurrence after transurethral resection of BC.^[4,5]^ Cisplatin-based regimens have become the first line of treatment for recurrent or advanced BC, but the prognosis is still poor.^[6,7]^ Systemic chemotherapy for BC patients remains challenging. Therefore, predictive markers used for prognosis stratification and clinical decision-making personalization are strongly desired.

Necroptosis is a molecularly defined necrotic cell death pathway.^[8]^ Necroptosis regulate tumor immune response mainly by promoting the release of DAMPs, cytokines, or chemokines to promote the interaction between dying cancer cells and immune cells. Necroptosis form an immune suppression microenvironment by CXCL1 and promote the progression of pancreatic tumor.^[9]^ CD8 + T cells could be activated by necroptotic cancer cells and enable anti-tumor immunity in a study using genetic methods.^[10]^ With the increasing attention to immunogenic apoptosis and severe apoptosis resistance, necrotic apoptosis would provide a new idea for the immunotherapy of BC.

Noncoding RNAs with lengths longer than 200 nucleotides are classified as long noncoding RNA (lncRNAs), which play important roles in tumorigenesis and BC resistance to chemotherapy drugs.^[11]^ Unlike common tumor markers, LncRNA is increasingly proven to contribute to tumor immune cell infiltration of BC and regulate related immune pathways.^[12–14]^ LncRNA LNMAT1 could upregulate CCL2 expression and encourage macrophages to recruit to tumors to further accelerate lymph node metastasis of BC.^[15]^ However, the biological functions and therapeutic potential of necroptosis-related lncRNAs in BC are rarely studied.

In the context of tumors, hot refers to tumors that have immune cell infiltration, while cold refers to tumors that lack immune cell infiltration and immunotherapy response rate remains relatively low in most cold tumors. With the increasing application of immunotherapy in BC, how to identify hot and cold tumors is very important.^[16]^ However, a convenient but accurate method for distinguishing tumors is still lacking.^[17]^ Therefore, the establishment of a necroptosis-related lncRNA model to accurately distinguish tumors and predict the prognosis of BC is very important to guide clinical diagnosis and treatment.

## 2. Materials and methods

### 2.1. Data processing

From the TCGA (https://portal.gdc.cancer.gov/) database, in addition to the fragments per kilobase of transcript per million mapped reads-standardized RNA-seq data we downloaded, we also collected the corresponding patients’ clinical and prognostic information. For normalizing RNA expression raw profiles, we use Strawberry Perl to match transcription and human configuration data and get synthetic data matrices. Then we use the synthetic data matrices to identify differentially expressed lncRNAs. For the clinical data, those whose overall survival (OS) is less than thirty days or without OS values were eliminated to increase the study reliability.^[18]^ To distinguish the train and test risk group, the caret R package was used to spilt 400 patients into 2 at a ratio of 1:1. The data used in our study were accessed from the TCGA database, a freely available database, thus ethical approval of Beijing Hospital was unnecessary for our study.

### 2.2. Necroptosis-related LncRNAs screening

First, mRNA and lncRNA synthetic data matrices were divided by Strawberry Perl. According to the previous reports of necroptosis, 67 necroptosis-related genes were collected (Supplementary Table 1, http://links.lww.com/MD/K118). Limma R package was used to screen the mRNA and lncRNA synthetic data matrices. LncRNAs under a cutoff value of *P* < .001 and Pearson correlation coefficients >0.4 by correlation analysis were selected. And we obtained 892 necroptosis-related lncRNAs. Then, 696 lncRNAs were considered differentially expressed necroptosis-related lncRNAs in BC patients (False discovery rate (FDR) > 0.05, log2 fold change (FC) > 1, and *P* < .05).

### 2.3. Construction of risk signature and its verification

Univariate Cox proportional hazard regression was used in clinical and genetic data merging matrices to confirm lncRNAs with a clear correlation to OS (*P* < .01). The least absolute shrinkage and selection operator (LASSO) method is a commonly used variable selection method, which reduces the number of variables by penalizing feature weights, effectively reducing overfitting and optimizing models. LASSO was performed to get the coefficient of each targeted gene through the L1-norm method under R package “caret” and established the risk model. The equation applied for calculating the risk score was as below:


$$\begin{array}{l} risk\;score=\sum_{k=1}^{n}Expr\left(IncRNA_{k}\right)*\left(coef_{k}\right) \end{array}$$

Coef was interpreted as the coefficient and expr was interpreted as lncRNA expression. Based on the median score, we divided every subject into a high/low-risk group.

### 2.4. Independence factors and ROC

We evaluated the prognostic value of clinical characteristics and risk score by univariate Cox and multivariate Cox regression analyses. ROC curve evaluating the accuracy of different factors in predicting survival was developed.

### 2.5. Gene set enrichment analyses

We identified annotated gene set (kegg.v7.4.symbols.gmt) as a reference set. GSEA (version 4.2) was applied for screening the enriched pathways between 2 risk groups, *P* < .05 and FDR < 0.25 as selection criteria.

### 2.6. Tumor microenvironment (TME) and immune checkpoints

First, immune cellular factors in risk populations were analyzed. Several databases were used to assess the statuses of immune infiltration respectively on the platform of TIMER 2.0 (http://timer.cistrome.org/). TIMER, CIBERSORT, XCELL, QUANTISEQ, MCP counter, EPIC, and CIBERSORT are all included in this step. The difference between the immune infiltrating cells studied by limma, scales, ggplot2, and ggtext R packages was assessed by Wilcoxon signed-rank test and the results were shown in a bubble graph. In addition, we used the GGPUBR software package to compare groups at low and high risk in terms of TME scores and immune checkpoint activation.

### 2.7. The role of the signature in predicting the clinical treatment response

Chemoresistance may be associated with a poor prognosis, so it necessary to test its relationship with the signature. The analyses for IC50 could reflect the therapy response of different widely used drugs. To calculate half-maximal inhibitory concentration (IC50), Genomics of Drug Sensitivity in Cancer (https://www.cancerrxgene.org)-based PRRophetic R package was used.

### 2.8. Characterization of clusters

Cluster analysis, calculated using the “ConsensusClusterPlus (CC)” R package, was applied to classify potential subgroups based on different lncRNA expressions. Principal component analysis was conducted to characterize the independent principal components of the clusters. By t-distributed stochastic neighbor embedding analysis with the Rtsne R package, clustering visualization was exhibited. Kaplan–Meier (K–M) survival which was used for survival comparison was made between clusters. What more, the “GSVA” package was performed to analyze the difference between 2 clusters of immune-related functions. pRRophetic R package was also developed to obtain the IC50 values in order to identify the most suitable drugs.

## 3. Results and discussion

### 3.1. Results

#### 3.1.1. Differentially expressed necroptosis related LncRNAs.

**Figure 1.**
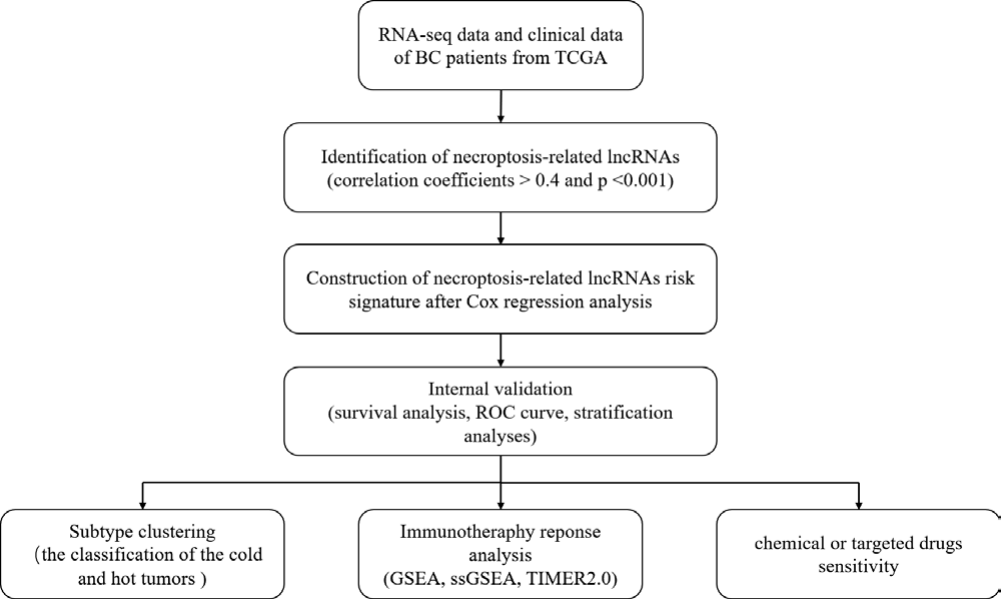
The flowchart of our study.

**Figure 2.**
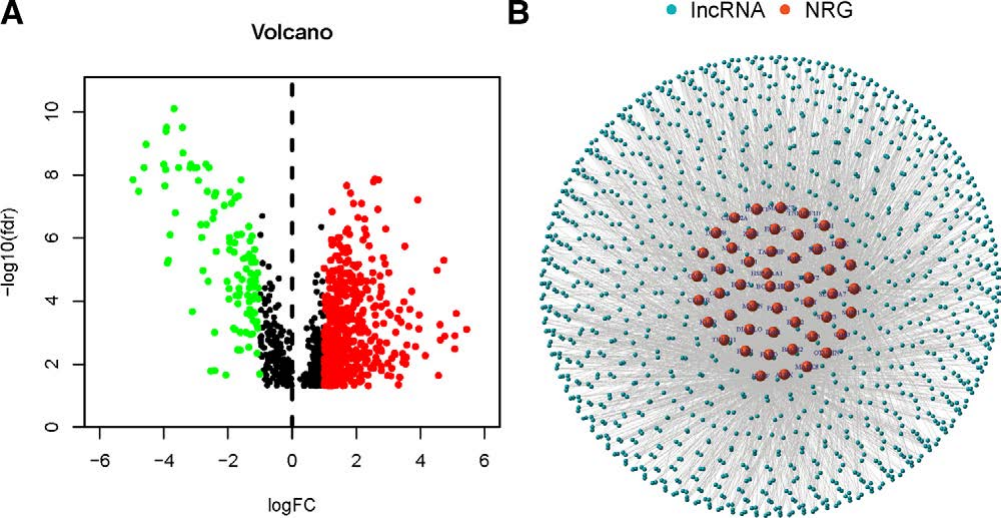
Differentially expressed necroptosis related LncRNAs in BC patients. (A) Differentially expressed necroptosis-related lncRNAs. (B) Network visualization. BC = bladder cancer, LncRNAs = long noncoding RNAs.

The flow of the study was exhibited in Figure 1. We found 19 normal samples and 414 tumor samples from the TCGA matrix. Through the correlation analysis of which correlation coefficients >0.4, we obtained 892 necroptosis-related lncRNAs (*P* < .001, Supplementary Table 2, http://links.lww.com/MD/K119). After comparing the tumor with the normal samples, we found 696 differentially expressed necroptosis-related lncRNAs (588 upregulated, 108 downregulated, |Log2FC| > 1 and *P* < .05, Fig. 2A). Figure 2B represent the network visualization and elucidated the correlational relationship between genes and lncRNAs.

#### 3.1.2. Gene signature construction.

**Figure 3.**
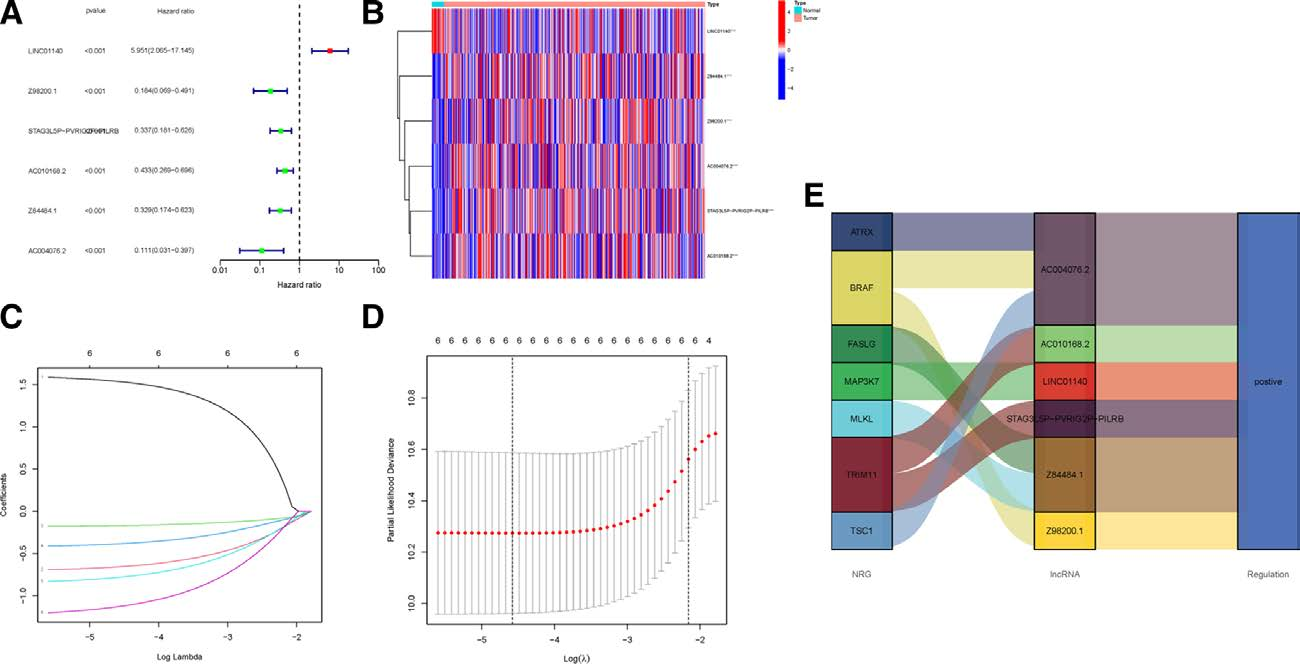
Gene signature construction of BC patients. (A) Analysis of univariate Cox. (B) This heatmap shows the 6 prognostic lncRNA profiles. (C) Variable selection. (D) The cross-validation plots. (E) Six prognostic lncRNAs related Sankey diagram. BC = bladder cancer, LncRNAs = long noncoding RNAs.

The univariate Cox and multivariate Cox regression analysis screened 6 significant differentially expressed necroptosis-related prognostic lncRNAs (LINC01140, Z98200.1, AC010168.2, STAG3L5P-PVRIG2P-PILRB, Z84484.1, AC004076.2) (*P* < .05, Fig. 3A). The resulting lncRNAs were then used for a predictive signature construction. The heatmap in Figure 3B visualizes the 6 necroptosis-related lncRNAs expression in the cohort. By LASSO Cox regression analysis, we finally constructed the prognostic signature with 5 differentially expressed necroptosis-related lncRNAs including LINC01140, Z98200.1, AC010168.2, Z84484.1 and AC004076.2 (Fig. 3C and 3D). Besides, the Sankey diagram demonstrated that all 6 lncRNAs were upregulated (Fig. 3E).

**Figure 4.**
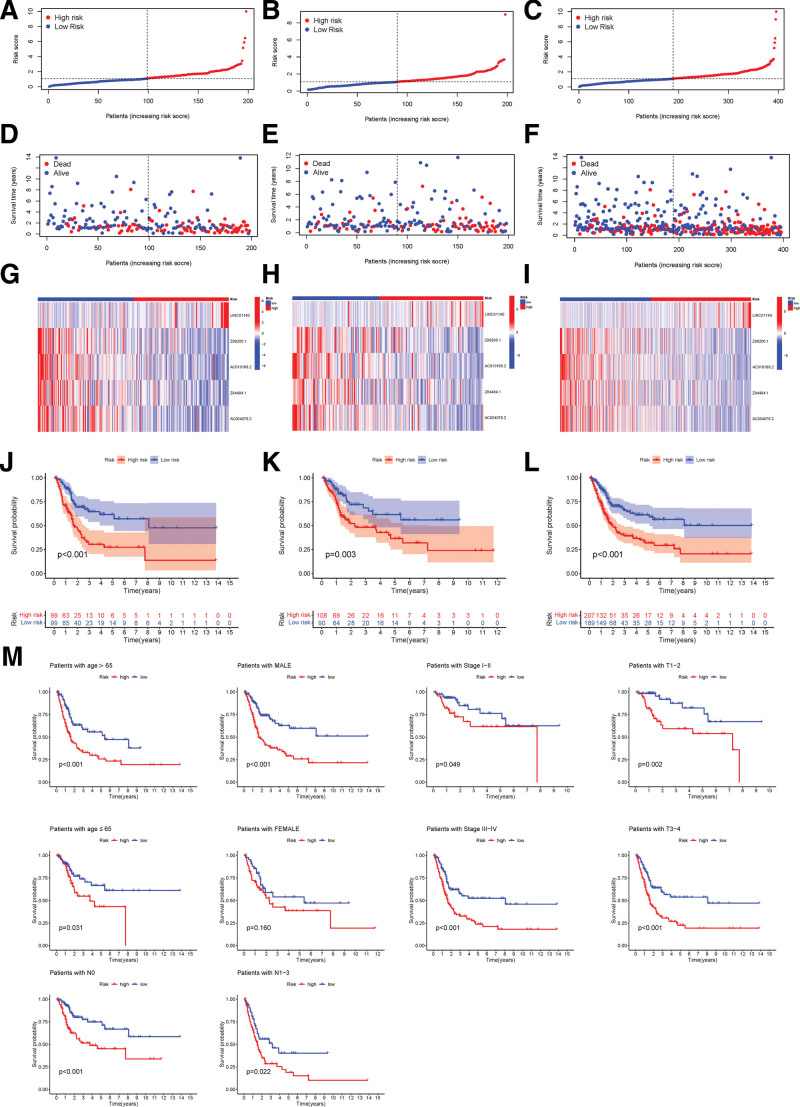
Prognostic analysis of the risk model in the 3 groups. (A–C) The distribution of risk score in the train, test, and entire sets, respectively. (D–F) Survival status of BC patients at different risk in the train, test, and whole sets, respectively. (G–I) The expression level of the 5 lncRNAs in the train, test, and whole sets respectively on the heatmap. (J–L) K–M curves for risk score in 3 groups. (M) K–M curves for age, gender, stage, T or N respectively in TCGA cohort. BC = bladder cancer. K–M = Kaplan–Meier, LncRNAs = long noncoding RNAs, TCGA = The Cancer Genome Atlas.

**Figure 5.**
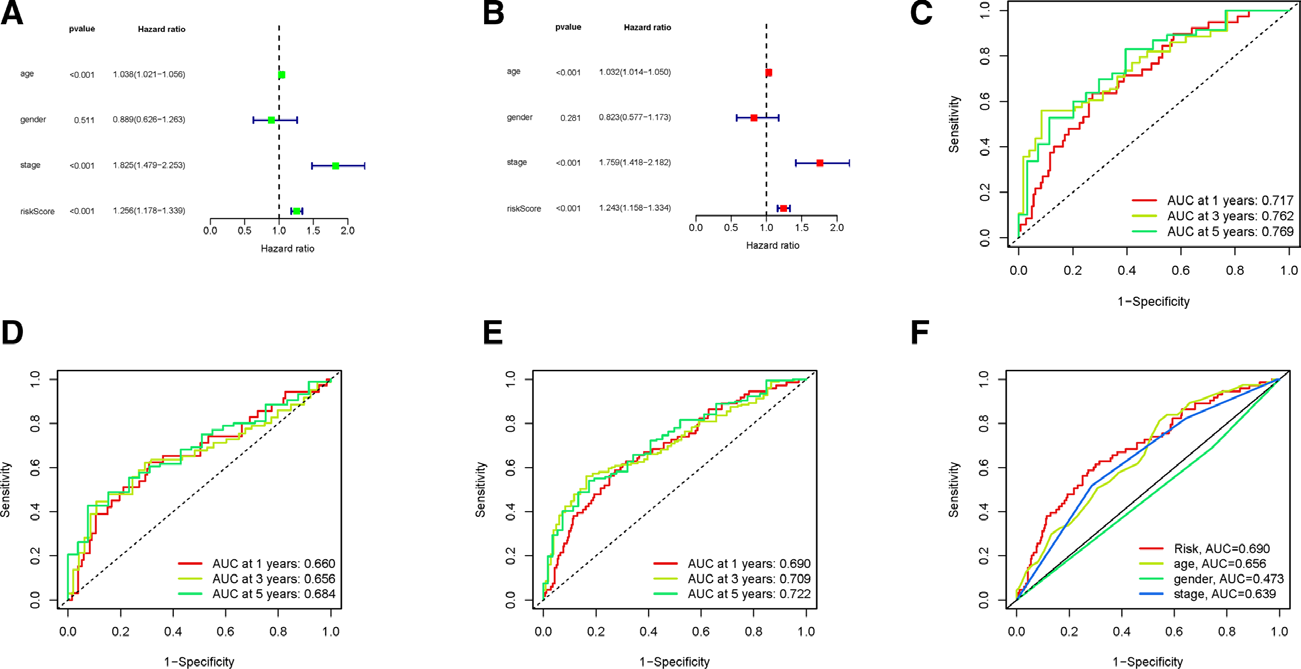
ROC curve plotting of the risk model. (A, B) Analyses of the risk score and clinical parameters using uni- and multi-Cox models. (C–E) ROC curves for 3 consecutive years of the train, test, and whole sets, respectively. (F) ROC curves for risk score and clinical parameters. multi-Cox = multivariate Cox, ROC = receiver operating characteristics.

We calculated the risk score as follows: risk score = LINC01140 × (1.6189) + Z98200.1 × (0.7836) + AC010168.2 × (0.4489) + Z84484.1 × (0.8783) + AC004076.2 × (1.4006). The same method of calculating the risk score was applied in the train, test, and all groups. With the risk score classification, we discovered that the higher the patient risk score, the poorer the patient survival (Fig. 4A–4I). We also verified that high risk was associated with reduced survival by K–M analysis (*P* < .001, Fig. 4J–4L). And as is shown in Figure 4M, age, gender, stage, T, and N had similar effects on it. We further confirmed the independent prognostic factors by univariate and multivariate analysis. The risk score and tumor stage were found to be the independent risk factors associated with the life expectancy that fit our idea (*P* < .001, Fig. 5A and 5B).

As previously stated, we randomly divided the TCGA data, on average, into a training and a testing cohort for further analysis. The sensitivity and specificity of the model in different cohorts were examined by time-dependent ROC. In the train set, our model revealed good predictive power with AUCs of 0.717, 0.762, and 0.769 for first, third, and fifth-year OS, respectively (Fig. 5C). In the test set in the first, third, and fifth years, the AUCs of OS were 0.660, 0.656, and 0.684, respectively (Fig. 5D). In all set in the first, third, and fifth years, the AUCs of OS were 0.690, 0.709, and 0.722, respectively (Fig. 5E). In the first year, when age, gender, and stage were used as predictors, they had AUCs for OS of 0.656,0.473, and 0.639, respectively (Fig. 5F). In the fifth year, when the predictive model, age, gender, and stage were collectively used as predictors, they had AUCs for OS of 0.623, which was lower compared to the AUCs of 0.691 predicted by the model alone (Supplementary Figure 3, http://links.lww.com/MD/K124). All in all, the ROC curves confirmed that the predictive model and risk score won good robustness and predictive performance.

#### 3.1.3. Gene set enrichment analyses.

**Figure 6.**
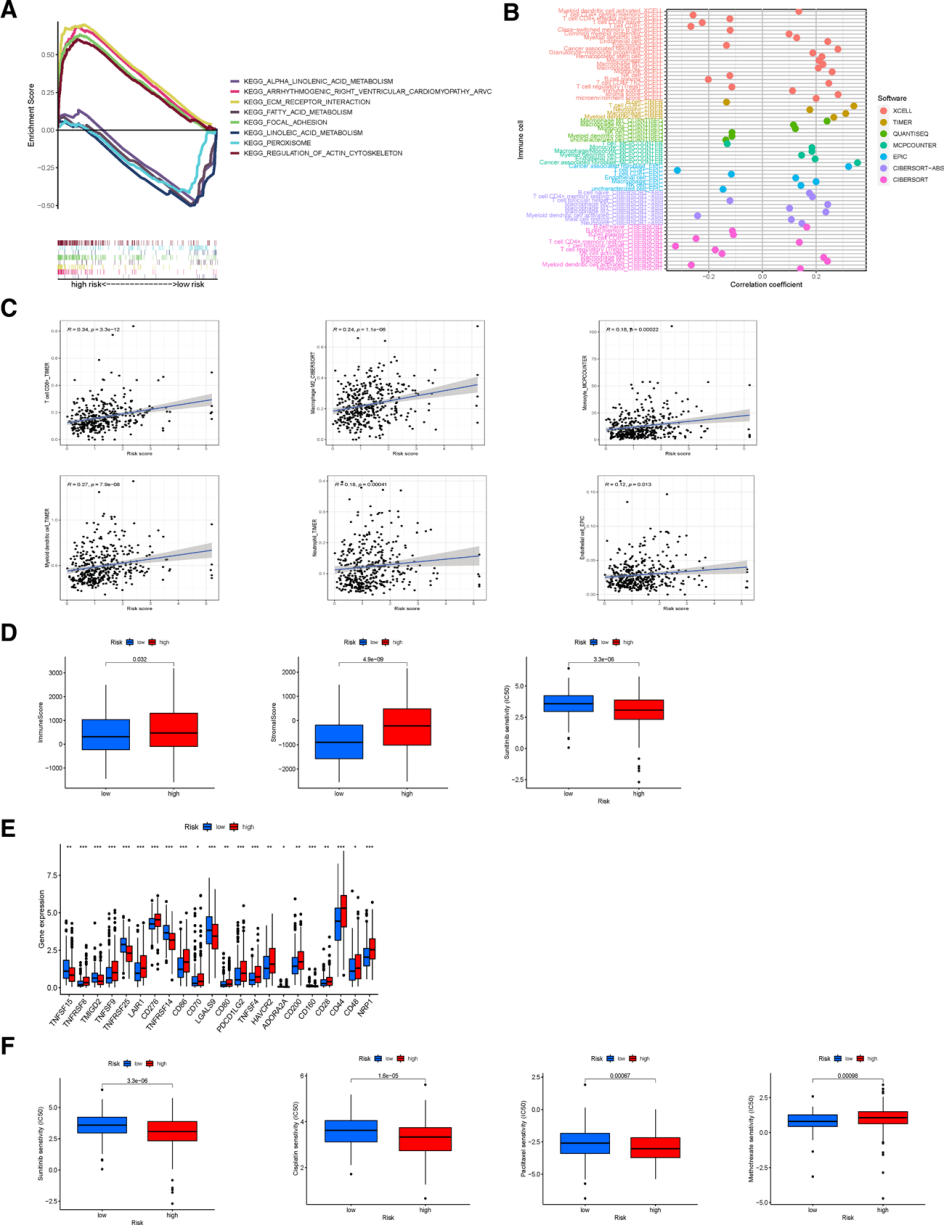
The immune characteristics of risk groups. (A) Top 4 KEGG pathways in risk groups. (B) Bubble plot of immune cells in risk groups. (C) The association of risk score with immune cells. (D) The immune-related scores of risk groups. (E) The checkpoints expression of risk groups. (F) Therapy response of common chemotherapy drugs for risk groups.

In order to investigate the cytological behavior in risk groups, different KEGG pathways in the entire set were explored by GSEA software (Supplementary Figure 1, http://links.lww.com/MD/K122). Immune and tumor-related pathways such as alpha-linolenic acid metabolism, ECM receptor interaction, and focal adhesion were illustrated as the first 4 pathways enriched in high and low-risk groups as shown in Figure 6A (*P* < .05, FDR < 0.25, |NES| > 1.5).

#### 3.1.4. Immune characteristics and therapy response of risk groups.

As is shown in the immune cell bubble chart, although on a different platform, the risk score was generally correlated with immune cells (Supplementary Table 3, http://links.lww.com/MD/K120). In the high-risk group, immune cells infiltrate at a greater rate, such as T cell CD8 + and macrophage at TIMER, Monocyte at XCELL, Macrophage M1 at CIBERSORT-ABS was found (all *P* < .05) (Fig. 6B). In the Figure 6C, the risk score further revealed a positive association with part of the immune cells like Macrophage M2 which play a prominent role in establishing the TME in BC.^[19]^ There were higher stromal scores, immune scores, and ESTIMATE scores in the high-risk group, which indicated that significant differences in TME exist in the 2 groups (Fig. 6D). Besides, the expressions of most immune checkpoint genes were positively correlated with risk scores and were significantly higher in the high-risk group (Fig. 6E). In addition, the predictive signature was also utilized to analyze the efficacy of the US Food and Drug Administration (FDA)-approved chemotherapeutic or targeted drugs for BC. Sunitinib, paclitaxel, cisplatin, and docetaxel were found to have lower IC50 in the higher immune score and higher risk group. However, a higher IC50 of methotrexate was found in the high-risk group (Fig. 6F).

#### 3.1.5. The classification of the cold and hot tumors and individualized treatment in clusters.

**Figure 7.**
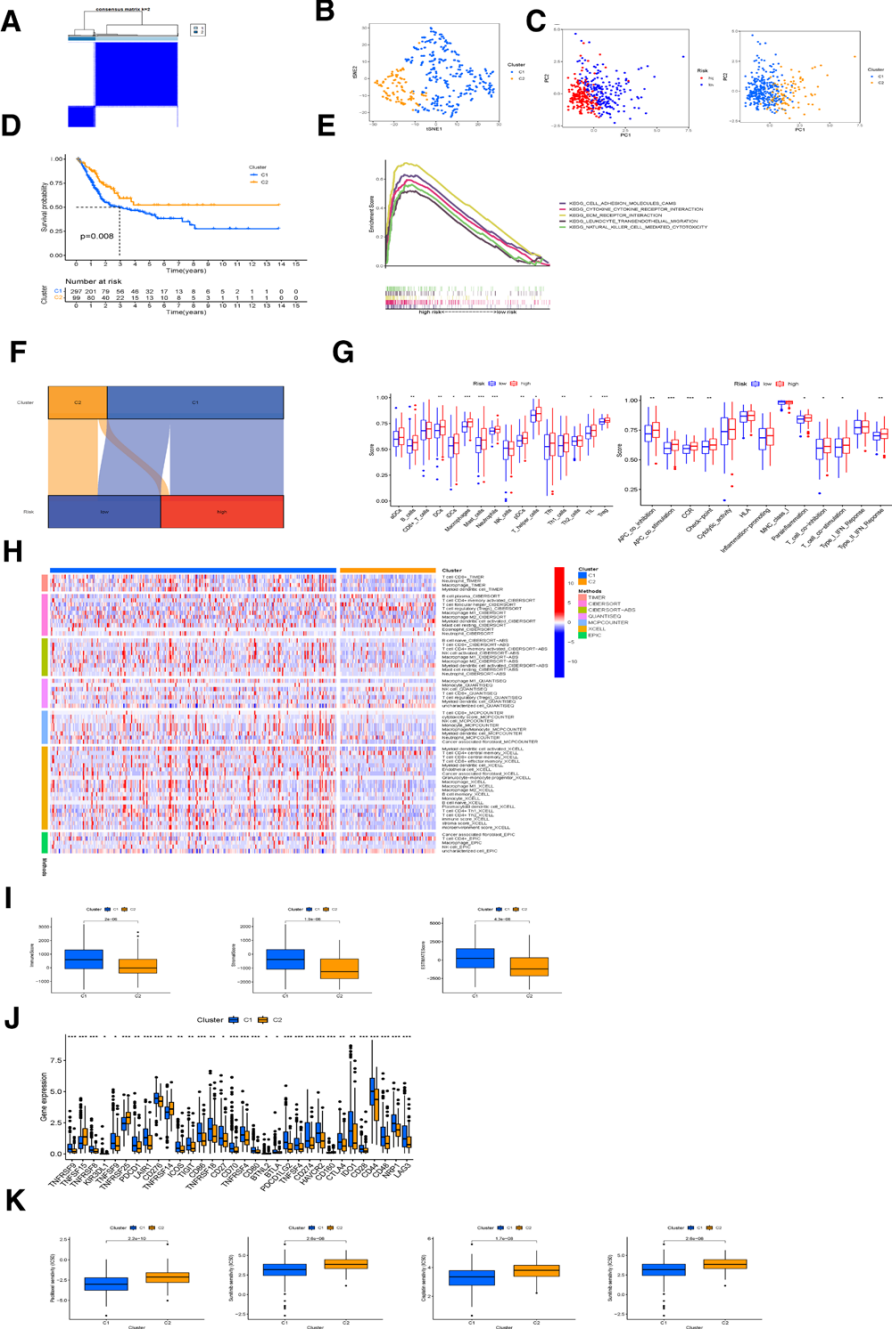
The classification of cold or hot tumors and individualized treatment in clusters. (A) Consensus clustering matrix (k = 2). (B) The plot of t-SNE. (C) PCA plots. (D) K–M curves of OS in clusters. (E) Top 4 pathways of cluster 2. (F) Sankey diagrams indicating correlation between risk groups and clusters. (G) The ssGSEA scores which represent 16-cell infiltration and immune functions in clusters. (H) Immune cells in clusters were visualized by heat map. (I) The differences in immune-related scores between the 2 clusters were detected. (J) The expression of 32 checkpoints in 2 clusters. (K) Therapy response of 4 FDA-approved chemotherapy drugs for clusters. FDA = US Food and Drug Administration. K–M = Kaplan–Meier, OS = overall survival, PCA = principal component analysis, ssGSEA = single sample GSEA.

According to Consensus clustering analysis and the 5 differentially expressed necroptosis-related lncRNAs, the 396 BC patients could be separated into 2 groups after setting clustering variable (k) to 2 which represent the highest intragroup correlations and the lowest intergroup correlations (Fig. 7A, Supplementary Figure 2, http://links.lww.com/MD/K123). TSNE verified the reliability of the classification between the 2 clusters (Fig. 7B). The principal component analysis was also used to indicate the difference between the 2 clusters (Fig. 7C). Cluster 1 exhibits decreased survival rate compared with Cluster 2 (*P* < .001, Fig. 7D). GSEA was conducted for biological functions between 2 clusters. In cluster 1, 4 of the top 5 pathways with enrichment were related to immunity, such as “natural killer (NK) cell-mediated cytotoxicity” (*P* < .05; FDR < 0.25; |NES| > 1.9, Fig. 7E), which involved in immune regulation and tumor progression.^[20]^ The enrichment scores of immune cells and the activity of immune-related functions were evaluated across low-and high-risk groups by the single sample GSEA (Fig. 7G). Besides, from Figure 7F and Supplementary Table 4, http://links.lww.com/MD/K121, we could validate that cluster 1 and cluster 2 was correlated with the high-risk group and low-risk group respectively. So among 16 kinds of immune cells, except for NK cells, all other immune cells, such as antigen-presenting cells and CD8 + T cells, 2 critical cell types participating in immune therapy response, infiltrate at a greater rate in cluster 1. All 13 immune-related functions, such as inflammation − promoting, were found to express more in cluster 1 (Fig. 7G). This suggests that cluster 1 was of more active immunological function. When the immune cells were examined between the 2 clusters on different platforms (Fig. 7H), cluster 1 showed better infiltration. Cluster 1 also showed a higher immune score compared to cluster 2, as well as a higher ESTIMAT (microenvironment) score and a higher stromal, and result in a different TME (Fig. 7I). Potential changes in immune checkpoints between the 2 clusters were further investigated. The majority of the immune checkpoints showed increased activation in cluster 1, such as PD-1, LAG3, BTLA, and HAVCR2 (Fig. 7J), which is consistent with the results in the high-risk group. According to intrinsic tumor parameters, hot tumors were highly infiltrated with antigen-presenting cells and crucial immune-associated factors. Hot tumors are of high response rates to immunotherapy. Therefore, cluster 1 should be regarded as the hot tumor that was more sensitive to immunotherapy while cluster 2 is the cold tumor that was less sensitive. Clinically, cisplatin, gemcitabine, paclitaxel, and docetaxel are commonly used for chemotherapy in urothelial carcinoma. When the drug sensitivity was compared, these 4 chemical or targeted drugs were found to have lower IC50 in cluster 1 (Fig. 7K).

## 4. Discussion

Immunotherapy, especially checkpoint inhibitors, plays an increasingly important role in modern tumor treatments. However, due to the heterogeneity of TME, the clinical outcome of immunotherapy may vary.^[21]^ Thus, hot and cold tumors have appeared in a new classification of tumors. Tumors with poor lymphocytic infiltration are called “cold tumors” with immunologic inertia, which often respond poorly to immunotherapy. The opposite was a hot tumor, which response well to immunotherapy. As we know, immune checkpoint inhibitor therapy or other immunotherapies specifically activate immune cells (mainly T cells) surrounding tumor cells to induce an immune response that kills the hot tumor. However, in cold tumors, these immune checkpoint inhibitors or immunotherapy do not work well. Therefore, how to accurately apply immunotherapy and immune checkpoint inhibitors is the key to enhancing the sensitivity of tumor immunotherapy, and also an important means to transform cold tumors into hot tumors.^[22]^

Firstly, the differentially expressed necroptosis-related lncRNAs in BC patients for prognosis were screened out. And then 5 lncRNAs (LINC01140, Z98200.1, AC010168.2, Z84484.1, AC004076.2) were aggregated to construct a novel risk signature based on the regression coefficients. According to the risk scores calculated by the 5 lncRNAs, molecular subtypes were regrouped in low and high-risk groups. Then a survival study measuring the predictive value of the necroptosis-related lncRNAs was conducted. Patients with low-risk necroptosis-related lncRNAs had a higher survival rate. Through IC50 prediction analyses and immune checkpoint comparison, the high-risk group was found to have better immunotherapy responses. This suggests that we can use appropriate immune checkpoint inhibitors for BC patients with different risks. The above indicates that our model can effectively predict the prognosis of BC patients and further guide their treatments. However, the ability of this risk signature for distinguishing hot/cold tumors needs to be explored to direct clinical treatments better than before. Clustering methodologies were widely used for classifying genetic subtypes. So different clusters have different TME leading to different immunotherapy responses based on their genetic features.^[23]^ To confirm the predictive ability of lncRNA related model in immunotherapy, the BC patients were divided into different groups based on the expression of these lncRNAs and we finally identified 2 clusters characterized by different immune infiltration. In the study, clusters 1 and 2 were redefined concisely as hot and cold tumor groups respectively. The immune score, stromal score, and tumor purity between the 2 clusters were calculated to ensure our hypothesis and classification. Several types of T cells, such as CD8 + T cells and M1 macrophage cells were mainly enriched in cluster 1, suggesting that this pattern was further confirmed to be a hot tumor that is more responsive to immunotherapy. There were also higher immune scores and greater activation of CTLA4, HAVCR2, and PD-L1 in cluster 1. Besides, cluster 1 was more sensitive to immunotherapeutic and chemotherapeutic drugs. Therefore, the lncRNA model can well distinguish cold and hot tumors which facilitate immunotherapy and guide clinical trials.

We developed a risk model containing 5 lncRNAs and a Sankey diagram of necroptosis genes and related lncRNAs. We found mRNA (TSC1, TRIM11, MLKL, MAP3K7, FASLG, BRAF) significantly co-expressed with these lncRNAs. LINC011400 could change macrophage M2 polarization through TME and modulate FGF9 to affect BC cell aggressiveness.^[24]^ MLKL, associated with Z84484.1, is a core necroptotic protein that inflicts membrane permeabilization and necroptosis. MLKL-mRNA treatment, combined with immune checkpoint blockade, has been proven to effectively enhance the anti-tumor activity.^[25]^ The protein encoded by TSC1, associated with AC004076.2, impacts the regulation of mTOR activity and participates in the functional regulation of tumor-associated macrophages.^[26]^ AC010168.2 was reported to modulate the protein-coding gene HIST4H4 to affect the survival of BC.^[27]^ But in our study, AC010168.2 was associated with TRIM11. The lncRNA Z98200.1 was first discovered. A deeper understanding of these lncRNAs will help us make better clinical decisions. Extending our gene signature study to BC patients, the FDA-approved drugs sensitivity for BC patients of different subgroups was compared by IC50. Besides, the immune cell bubble and the heatmap of immune cell infiltration were used to validate the immune checkpoint inhibitor sensitivity between risk groups and clusters. Our study demonstrates that anti-PD-1/L1 immunotherapy probably has anti-tumor efficacy in high-risk groups. What more, conventional chemotherapy drugs sunitinib, paclitaxel, cisplatin, and docetaxel were still effective for high-risk groups. However methotrexate was found to be resistant to them. This suggests that immunotherapy combined with chemotherapy may have better efficacy for high-risk groups, which provides support for a comprehensive evaluation and precise treatment of BC patients.

However, our research has some limitations. Firstly, we were unable to acquire data extracted from other databases to make external validation to estimate the model dependability. We have used GSE13507 in the GEO database, but we could not get appropriate information on lncRNAs.

Further validation in independent datasets or research to assess the generalizability of our model is necessary. Secondly, no further functional or mechanistic research on the necroptosis-related lncRNAs in BC was conducted and verified. Thirdly, to ensure the accuracy and reliability of our constructed model for necroptosis-associated lncRNA risk signatures, we only utilized tumor samples with available clinical data in both univariate and multivariate analyses. Therefore, the limited number of normal samples had no impact on the validity of our model. Importantly, our model is suitable for future clinical testing, thereby emphasizing its potential practical application. Last but not least, this is a retrospective study with some fixed biases.

## 5. Conclusions

In summary, we constructed a prognostic signature based on 5 necroptosis-related lncRNAs that demonstrated excellent predictive performance for survival, immune landscape, and therapeutic responses for patients with BC. However, since the data used in this study were obtained from public sources, further in vivo and in vitro experiments are necessary to provide more evidence. Our signature successfully stratified BC patients into low-risk and high-risk groups with distinct differences in immune cell infiltration. Furthermore, our classification system could differentiate between hot and cold tumors, providing a comprehensive understanding of the regulatory mechanism network involving immunity, necroptosis, and lncRNAs in BC. As a result, this research may guide future studies on BC-related molecules, and emphatically motivate practical engaged scholarship in academic research.

## Acknowledgments

We acknowledge the TCGA databases for providing its platform and thank all the researchers and study participants for their contributions.

## Author contributions

**Conceptualization:** Han Li.

**Methodology:** Zhengtong Lv.

**Supervision:** Ming Liu.

**Validation:** Ming Liu.

**Writing – original draft:** Han Li.

**Writing – review & editing:** Ming Liu.

## Supplementary Material
