# Supplementary material for: A five necroptosis-related lncRNA signature predicts the prognosis of bladder cancer and identifies hot or cold tumors
Source: Medicine (Baltimore). 2023 Oct 13;102(41):e35196. doi: 10.1097/MD.0000000000035196 (PMC10578762; doi:10.1097/MD.0000000000035196)
Supplement: Supplementary file 2 [file medi-102-e35196-s002.docx]

Necroptosis

gene lncRNA cor pvalue Regulation

BRAF AL929236.1 0.572322195086422 2.14813070004225e-37 postive

ATRX AL929236.1 0.423343849056197 1.96592976362548e-19 postive

ATRX AL356019.2 0.539255671524201 1.31428000439901e-32 postive

MAPK8 LINC02156 0.4261036180118 1.08374424977992e-19 postive

OTULIN LINC02156 0.462816035396552 2.28812352478589e-23 postive

BRAF LINC02156 0.734211583121545 2.73507774659999e-71 postive

ATRX LINC02156 0.544440572112928 2.52910917895002e-33 postive

ATRX AL162724.2 0.482953328909742 1.40064835422016e-25 postive

CD40 AC135507.1 0.408622154260677 4.3022055286402e-18 postive

SIRT1 PTPRG-AS1 0.497716392232579 2.67981971988885e-27 postive

TSC1 AL117336.3 0.497528105900424 2.82195113741615e-27 postive

BRAF LINC01068 0.453489629221093 2.16685419374502e-22 postive

MAPK8 AC009754.1 0.453614411303334 2.10360276171679e-22 postive

OTULIN AC009754.1 0.462598296038164 2.41333411771691e-23 postive

BRAF AC009754.1 0.733364647217796 4.76859337362294e-71 postive

ATRX AC009754.1 0.561343050992116 9.57542965041691e-36 postive

BRAF AC108463.2 0.424729766037547 1.45874455002134e-19 postive

AXL AC108463.2 0.400041289870561 2.42546678434308e-17 postive

ATRX AC108463.2 0.522912252858584 1.97388777010773e-30 postive

TSC1 AL121580.1 0.445752328169489 1.32844592508195e-21 postive

MAPK8 AL121580.1 0.481955644641899 1.81745273668465e-25 postive

IPMK AL121580.1 0.421911910375232 2.67197649619405e-19 postive

OTULIN AL121580.1 0.50739260499777 1.80191441318592e-28 postive

BRAF AL121580.1 0.800482764975379 1.24848501618821e-93 postive

ATRX AL121580.1 0.644475162188697 5.81291615414266e-50 postive

TSC1 AC092611.1 0.555615508111439 6.56649983894156e-35 postive

MAPK8 AC092611.1 0.451542880213487 3.43454974630405e-22 postive

OTULIN AC092611.1 0.408569089624251 4.34913661349476e-18 postive

BRAF AC092611.1 0.730874287732211 2.41514525692717e-70 postive

ATRX AC092611.1 0.523054348819268 1.89192307625218e-30 postive

AXL AC009549.1 0.461000351766366 3.56382030717332e-23 postive

TSC1 AC092123.1 0.451587488712954 3.39860259716211e-22 postive

BRAF AC092123.1 0.428044741027716 7.10524416184145e-20 postive

ATRX AC018653.3 0.439389126710456 5.70274999115559e-21 postive

BRAF AL359921.1 0.445801625675295 1.31337793984384e-21 postive

ATRX AL359921.1 0.535628477737824 4.0930994215854e-32 postive

TSC1 PHKA2-AS1 0.418011117719586 6.11856537665997e-19 postive

TSC1 AC011461.1 0.470315767157326 3.56712142373531e-24 postive

ATRX AC011461.1 0.415545989987368 1.02714353069961e-18 postive

RNF31 AC025766.1 0.47710093450298 6.37828403320856e-25 postive

TSC1 LINC01004 0.495820933058289 4.50206817598452e-27 postive

BRAF LINC01004 0.480531891426949 2.63190607597956e-25 postive

TSC1 AL137244.1 0.446652878150937 1.07824024738251e-21 postive

BRAF AL137244.1 0.481042949968733 2.30480915398365e-25 postive

TSC1 AC005387.2 0.411085947439599 2.59412025371609e-18 postive

BRAF AC005387.2 0.489126417580206 2.74160811630001e-26 postive

ATRX AC005387.2 0.430909751921962 3.79135230425788e-20 postive

OTULIN AL110115.1 0.43334036596074 2.21475177228121e-20 postive

BRAF AL110115.1 0.589530812899935 4.15885083863067e-40 postive

ATRX AL110115.1 0.569179490854923 6.46292256973108e-37 postive

TNFRSF1B AC107959.1 0.404577227318823 9.78275184474196e-18 postive

BRAF AL358334.3 0.434655128120009 1.65291464600516e-20 postive

ATRX AL358334.3 0.450411029661427 4.48316843463014e-22 postive

MAP3K7 AL606763.1 0.40154335214638 1.79837772586733e-17 postive

MAP3K7 SOX1-OT 0.476362488525817 7.70690234835546e-25 postive

TSC1 PRC1-AS1 0.414240398975167 1.34907809441922e-18 postive

BRAF PRC1-AS1 0.497375379670057 2.94268908828862e-27 postive

ATRX PRC1-AS1 0.59448295152821 6.42337599225408e-41 postive

BRAF AC016727.1 0.442052408290319 3.11081244718946e-21 postive

ATRX AC016727.1 0.401244235475 1.90899297582663e-17 postive

TSC1 AC008121.2 0.428233079266193 6.81909154981616e-20 postive

MAPK8 AC008121.2 0.435085156888969 1.50164334955356e-20 postive

BRAF AC008121.2 0.746824318217679 5.35121491092386e-75 postive

ATRX AC008121.2 0.43833401685399 7.23969342572879e-21 postive

TSC1 AC025031.1 0.442040204275925 3.11950180133335e-21 postive

MAPK8 AC025031.1 0.441763801987913 3.32283515860088e-21 postive

OTULIN AC025031.1 0.47881991176558 4.09861656097833e-25 postive

BRAF AC025031.1 0.811958350745775 2.28570280792192e-98 postive

ATRX AC025031.1 0.557725944913816 3.2442344724118e-35 postive

TSC1 AC114980.1 0.402364189757086 1.52618456655532e-17 postive

BRAF AC114980.1 0.534933556331747 5.08035223075293e-32 postive

ATRX AC114980.1 0.571232699928119 3.15123901753794e-37 postive

ATRX EIPR1-IT1 0.52320530245447 1.80853455343946e-30 postive

DNMT1 AL449423.1 0.460721817370651 3.81359694792602e-23 postive

CDKN2A AL449423.1 0.581294231916975 8.66275355104259e-39 postive

DDX58 AL445490.1 0.45862161165973 6.34297829107632e-23 postive

TSC1 CRTC3-AS1 0.426243311622432 1.05140885087181e-19 postive

MAPK8 CRTC3-AS1 0.443866351150634 2.05244151329881e-21 postive

BRAF CRTC3-AS1 0.6883892659471 1.98950553671039e-59 postive

ATRX CRTC3-AS1 0.506872796220807 2.08770914331294e-28 postive

TSC1 AC092301.1 0.583525530195139 3.83822850039543e-39 postive

BRAF AC092301.1 0.502870198953911 6.43172837653704e-28 postive

ATRX AC092301.1 0.575175761350425 7.82027639519372e-38 postive

TSC1 AC104532.2 0.451965602304065 3.10843427049971e-22 postive

BRAF AC104532.2 0.495313502253009 5.16999656224143e-27 postive

FAS AL157394.1 0.786534720432365 2.84210064588629e-88 postive

CYLD AL157394.1 0.410843496898973 2.72703428493685e-18 postive

CFLAR AL157394.1 0.446830099001023 1.03478197414653e-21 postive

BRAF AC106028.4 0.423447521970958 1.92262624010243e-19 postive

BRAF LINC01535 0.417985542898779 6.15167299990086e-19 postive

BRAF AC106771.1 0.475111297735015 1.06086613400143e-24 postive

TSC1 AC108449.2 0.490967628905635 1.67470105602769e-26 postive

MAPK8 AC108449.2 0.470387690399465 3.50332399773056e-24 postive

IPMK AC108449.2 0.509823653581192 9.02104779908666e-29 postive

OTULIN AC108449.2 0.494080961349177 7.22772736421197e-27 postive

BRAF AC108449.2 0.718513467280907 5.84239882323168e-67 postive

SIRT1 AC108449.2 0.437239407500594 9.26517696889048e-21 postive

ATRX AC108449.2 0.77637102487591 1.28881711205541e-84 postive

TNFRSF1B VIM-AS1 0.415097219426514 1.12820803743338e-18 postive

AXL VIM-AS1 0.407498280416541 5.41136163041849e-18 postive

TSC1 AC006480.2 0.489475140306347 2.49781747870574e-26 postive

BRAF AC006480.2 0.584457589904741 2.72675668091179e-39 postive

ATRX AC006480.2 0.549261891240303 5.32317984772804e-34 postive

TSC1 AC136604.2 0.459415713033913 5.23512497165561e-23 postive

BRAF AC136604.2 0.586523478546618 1.27297591039757e-39 postive

ATRX AC136604.2 0.599635279404536 8.88602457919945e-42 postive

KLF9 AC106881.1 0.435397790768727 1.40031317207892e-20 postive

BRAF AL078587.1 0.552880829463375 1.625165878479e-34 postive

TSC1 AL158163.2 0.427652235496948 7.74013413845209e-20 postive

BRAF AL158163.2 0.557869160999242 3.09209216871348e-35 postive

ATRX AL158163.2 0.476623882601802 7.20798916570784e-25 postive

BRAF AC012568.1 0.444564076783018 1.74788814642077e-21 postive

ATRX AC012568.1 0.589638784831819 3.99425146029532e-40 postive

BRAF SRRM2-AS1 0.415008579542446 1.14929724215221e-18 postive

BRAF AC007546.1 0.558103634160716 2.85811007006876e-35 postive

ATRX AC007546.1 0.447385634359537 9.09440360638202e-22 postive

TSC1 HM13-IT1 0.539796597757675 1.10814881027532e-32 postive

ATRX HM13-IT1 0.498151561563618 2.37782381511348e-27 postive

IPMK AC025031.2 0.417895126583681 6.27014013726146e-19 postive

OTULIN AC025031.2 0.402241829388517 1.56402690208729e-17 postive

BRAF AC025031.2 0.551260423448713 2.76944113274948e-34 postive

ATRX AC025031.2 0.639656507659904 5.1228144720539e-49 postive

TRIM11 AL670729.1 0.83740056752461 3.86687767445726e-110 postive

MAP3K7 LINC02241 0.420951030440502 3.28020949112831e-19 postive

BRAF AP001205.1 0.429638809974069 5.01330724229626e-20 postive

TSC1 AL512770.1 0.505446443456692 3.1228212027453e-28 postive

TSC1 AC021491.4 0.448445899150503 7.10341641213771e-22 postive

BRAF AC021491.4 0.744952190878928 1.96209029171124e-74 postive

TSC1 AC012170.2 0.412336727348155 2.00335709425386e-18 postive

BRAF AC012170.2 0.602290262227395 3.16174196108791e-42 postive

ATRX AC012170.2 0.608260291571175 2.98731244946818e-43 postive

TSC1 AC105137.2 0.444370493462795 1.82761124642733e-21 postive

BRAF AC105137.2 0.557387911182158 3.63335359425853e-35 postive

ATRX AC105137.2 0.453576626552561 2.12256099241508e-22 postive

TSC1 C1orf220 0.537356908155261 2.38616300565156e-32 postive

BRAF C1orf220 0.563810948097928 4.1288374902129e-36 postive

ATRX C1orf220 0.448414744683666 7.15526471927778e-22 postive

TSC1 AC126118.1 0.419345337924837 4.61429658737048e-19 postive

MAP3K7 LINC01967 0.429986395726839 4.64507801758142e-20 postive

TSC1 UBE2Q1-AS1 0.447351147055267 9.16765580789265e-22 postive

BRAF UBE2Q1-AS1 0.542922592455334 4.10957951808496e-33 postive

ATRX UBE2Q1-AS1 0.553301640561973 1.41440103570318e-34 postive

TSC1 AC012358.1 0.445010600602328 1.57683636256997e-21 postive

BRAF AC012358.1 0.577280756662762 3.68754641453875e-38 postive

ATRX AC012358.1 0.579203951179453 1.84661738154968e-38 postive

PLK1 AL133355.1 -0.436547060690585 1.08247032549885e-20 negative

TSC1 AC062037.2 0.406769489084104 6.27628342923117e-18 postive

FADD LINC02584 0.480365358855695 2.74808675072935e-25 postive

BRAF AC011815.1 0.432285628660364 2.79812896804308e-20 postive

ATRX AC011815.1 0.470525233412166 3.38444032499283e-24 postive

DNMT1 LINC01775 0.441996941678337 3.15049824545793e-21 postive

BACH2 AC010247.2 0.432740128316674 2.53020253632183e-20 postive

FLT3 AC087521.1 0.429012822769473 5.7503721784851e-20 postive

KLF9 AC087521.1 0.58295636402273 4.72681132760819e-39 postive

KLF9 AC002398.2 0.514199514346015 2.56024008073575e-29 postive

BRAF AP001160.4 0.410744858121902 2.78300631584548e-18 postive

TSC1 AL031600.1 0.466532240235643 9.16243739066511e-24 postive

ATRX AL031600.1 0.498584795466367 2.11062315957921e-27 postive

MAP3K7 AC008555.1 0.465135239089528 1.29419123459875e-23 postive

TSC1 AL513365.2 0.49535796824758 5.10775003106135e-27 postive

MAPK8 AL513365.2 0.441539777705002 3.49717385122915e-21 postive

OTULIN AL513365.2 0.429057912025215 5.69389177664712e-20 postive

BRAF AL513365.2 0.613745849539469 3.26911112610797e-44 postive

ATRX AL513365.2 0.634959343995011 4.11785098919151e-48 postive

TSC1 AC087289.1 0.475901056315326 8.67217226169335e-25 postive

MAPK8 AC087289.1 0.425090605088037 1.34940113612406e-19 postive

BRAF AC087289.1 0.652702130590163 1.29072057051569e-51 postive

ATRX AC087289.1 0.548344542024796 7.17449057304543e-34 postive

TSC1 AC021321.1 0.503839955639969 4.90372905332975e-28 postive

BRAF AC021321.1 0.632441796677161 1.23992597465173e-47 postive

TSC1 AL590652.1 0.437745035383993 8.26826306824196e-21 postive

BRAF AL590652.1 0.468163641355435 6.10917517944299e-24 postive

KLF9 AL136084.3 0.562495461965822 6.47059835490201e-36 postive

STAT3 AP000866.5 -0.405426248676805 8.24096662007931e-18 negative

BRAF AP000866.5 0.426401580581839 1.01592064747707e-19 postive

TSC1 U73166.1 0.418637215585901 5.36058806704545e-19 postive

BRAF LINC00515 0.419715328571299 4.26605186698425e-19 postive

TSC1 AC008115.3 0.472045963178582 2.30813148597992e-24 postive

BRAF AC008115.3 0.502546642070213 7.03960070610748e-28 postive

ATRX AC008115.3 0.624071325550376 4.50544943047908e-46 postive

TSC1 AL031709.1 0.488237387391 3.47460066160147e-26 postive

BRAF AL031709.1 0.419850067010077 4.14576880294064e-19 postive

ATRX AL031709.1 0.453383838147637 2.221945417024e-22 postive

BRAF AC118755.1 0.475719773020069 9.08323377284557e-25 postive

BRAF PIK3IP1-AS1 0.457652454538346 8.01201995542935e-23 postive

TSC1 AL109923.1 0.409985588585078 3.2533880036331e-18 postive

MAPK8 AL109923.1 0.440295190899646 4.64300119178923e-21 postive

OTULIN AL109923.1 0.49109165593176 1.6198344477927e-26 postive

BRAF AL109923.1 0.758293637947117 1.4447503496277e-78 postive

ATRX AL109923.1 0.604194974434105 1.49749972149195e-42 postive

TSC1 AC019183.1 0.420231868565809 3.82276672755224e-19 postive

IPMK AC019183.1 0.406621313238978 6.46809001478909e-18 postive

OTULIN AC019183.1 0.441846791858777 3.26044966343175e-21 postive

BRAF AC019183.1 0.612233268624856 6.04301938685384e-44 postive

ATRX AC019183.1 0.611863562082652 7.01863140241522e-44 postive

TSC1 AC010201.1 0.401544657263516 1.79790916992861e-17 postive

BRAF AC010201.1 0.447432892125288 8.99496276386472e-22 postive

TSC1 DLEU7-AS1 0.44230643615586 2.93526311339609e-21 postive

MAPK8 DLEU7-AS1 0.41842674167269 5.60446770553231e-19 postive

OTULIN DLEU7-AS1 0.409420286774557 3.65358479153735e-18 postive

BRAF DLEU7-AS1 0.754778987094362 1.88070816275915e-77 postive

ATRX DLEU7-AS1 0.514506705485982 2.34198489522463e-29 postive

MAPK8 AC007285.2 0.4144439081834 1.29304781541461e-18 postive

OTULIN AC007285.2 0.419416868054644 4.54485716260756e-19 postive

BRAF AC007285.2 0.599672646680619 8.7583089103919e-42 postive

ATRX AC007285.2 0.52147883351 3.02442234949259e-30 postive

CYLD LINC02100 0.448420469384427 7.14570958188153e-22 postive

TSC1 AC010422.4 0.464731329340308 1.42966872186053e-23 postive

OTULIN AC010422.4 0.400081391638809 2.40622072976296e-17 postive

BRAF AC010422.4 0.679450318064019 2.28945805314571e-57 postive

ATRX AC010422.4 0.605314890786978 9.62746718491582e-43 postive

BRAF AC084781.1 0.651024671298412 2.83245617544258e-51 postive

SLC39A7 AC092803.1 0.46573839629063 1.1151306275726e-23 postive

ATRX GNG12-AS1 0.650944902610297 2.93995533107226e-51 postive

LEF1 GNG12-AS1 0.407740182270095 5.15106689907789e-18 postive

TSC1 AC006435.2 0.516627149273273 1.26298423617457e-29 postive

BRAF AC006435.2 0.439194608722687 5.95960760039327e-21 postive

OTULIN ARAP1-AS2 0.419703536649079 4.27674070629842e-19 postive

BRAF ARAP1-AS2 0.504358568652389 4.24007082690588e-28 postive

ATRX ARAP1-AS2 0.645154936270098 4.26277911576143e-50 postive

BRAF AC091982.1 0.55457098432847 9.29151776848083e-35 postive

ATRX AC091982.1 0.559845134924371 1.59005207522727e-35 postive

TSC1 AC011503.2 0.408991684338409 3.9889194638759e-18 postive

BRAF AC011503.2 0.599531937667082 9.24892008293238e-42 postive

ATRX AC019080.5 0.463910467369785 1.74955896485975e-23 postive

TSC1 AC073957.3 0.573072542189293 1.64847913208284e-37 postive

BRAF AC073957.3 0.64343465286329 9.33072398935544e-50 postive

ATRX AC073957.3 0.428485247928478 6.45365979978966e-20 postive

TSC1 AL031775.2 0.440775820525149 4.1622456319571e-21 postive

BRAF AL031775.2 0.489458257068438 2.50911102402115e-26 postive

ATRX AL031775.2 0.414302522182879 1.33172566407656e-18 postive

TSC1 AL161452.1 0.415861925278581 9.6139231072412e-19 postive

TSC1 CCDC183-AS1 0.467720354494084 6.82192322858123e-24 postive

TSC1 AC027243.2 0.459350247502583 5.31873057404543e-23 postive

OTULIN AC027243.2 0.400957239873609 2.0214017717793e-17 postive

BRAF AC027243.2 0.695914894947155 3.19740360771293e-61 postive

ATRX AC027243.2 0.475504128497916 9.59731405021605e-25 postive

BRAF AC005225.2 0.694707376913095 6.25721130624331e-61 postive

SIRT3 AP006621.3 0.402405932396795 1.51348191841466e-17 postive

KLF9 AC079313.2 0.447385866470929 9.09391255395911e-22 postive

BRAF AL132656.2 0.477456615814762 5.82172299594867e-25 postive

MYC AL604028.1 -0.409732762244341 3.42674032539311e-18 negative

MAP3K7 AC010271.2 0.430880324226692 3.8160077486318e-20 postive

BRAF G2E3-AS1 0.483957738756331 1.07660607246996e-25 postive

ATRX AC090772.1 0.47648415785406 7.47057337634592e-25 postive

TRIM11 AL132655.2 0.423134777890243 2.05619869518607e-19 postive

TSC1 AL031670.1 0.459976551002451 4.57001655654359e-23 postive

MAPK8 AL031670.1 0.40010014468592 2.39727216565475e-17 postive

OTULIN AL031670.1 0.468618336191763 5.45446717348983e-24 postive

BRAF AL031670.1 0.530049234139919 2.28753158886423e-31 postive

ATRX AL031670.1 0.641018394684794 2.7804110743486e-49 postive

ATRX AC005740.4 0.44619154825464 1.19998528236467e-21 postive

TSC1 AC021491.2 0.523107322108913 1.862235775811e-30 postive

MAPK8 AC021491.2 0.41477547593317 1.20662626698362e-18 postive

BRAF AC021491.2 0.777611210027998 4.72514063869648e-85 postive

ATRX AC021491.2 0.491672702611539 1.38553640601678e-26 postive

DNMT1 AP003392.4 0.425309543267213 1.28703398064798e-19 postive

TSC1 AC073575.2 0.425895384959602 1.13377037153386e-19 postive

TSC1 AC010271.1 0.409173262077747 3.84331159093178e-18 postive

TSC1 AC112493.1 0.418818017308042 5.1594524390876e-19 postive

BRAF AC112493.1 0.626531502149318 1.58513391982934e-46 postive

ATRX AC112493.1 0.437024450587167 9.72404060859071e-21 postive

TSC1 AC024075.1 0.499739970953148 1.53465950841255e-27 postive

BRAF AC024075.1 0.474149985084024 1.35488080832616e-24 postive

ATRX AC024075.1 0.664759730205245 3.90879984916223e-54 postive

FASLG U62317.4 0.439701217219472 5.31325284359188e-21 postive

ZBP1 U62317.4 0.686815404482204 4.64535342159926e-59 postive

TSC1 AC010491.1 0.42127386417625 3.06202191901929e-19 postive

TNFRSF1B AC131097.4 0.466942587763993 8.27605482517311e-24 postive

AXL AC131097.4 0.420498418398055 3.61208276746903e-19 postive

TSC1 AL158835.2 0.416782302282668 7.92556362588052e-19 postive

BRAF AL158835.2 0.49063882603631 1.82919790406952e-26 postive

MAPK8 AC079336.2 0.404647859706321 9.64434477361744e-18 postive

BRAF AC079336.2 0.532504960116394 1.07682596069525e-31 postive

ATRX AC079336.2 0.43945415991056 5.61933257729127e-21 postive

MAPK8 AL356599.1 0.415981791224546 9.37545460855982e-19 postive

BRAF AL356599.1 0.551513697919282 2.54854420276369e-34 postive

SIRT1 AL356599.1 0.418509520318711 5.50727474943506e-19 postive

ATRX AL356599.1 0.626147311866112 1.86715622037739e-46 postive

TRIM11 TFAP2A-AS1 0.430592967514376 4.06521803058e-20 postive

BACH2 AL121820.1 0.436548813466031 1.08204457406016e-20 postive

BRAF AC008731.1 0.547315476613696 1.00166192672469e-33 postive

ATRX AC008731.1 0.607743823741407 3.67098111132225e-43 postive

MAPK8 LINC02109 0.406578595603176 6.52444850954561e-18 postive

OTULIN LINC02109 0.441190111073254 3.78746126429115e-21 postive

BRAF LINC02109 0.780994760165063 2.95831162689015e-86 postive

ATRX LINC02109 0.432203725691808 2.84929820030134e-20 postive

TSC1 FAM13A-AS1 0.489509867042604 2.47474565408189e-26 postive

OTULIN FAM13A-AS1 0.404695865807582 9.55137578119527e-18 postive

BRAF FAM13A-AS1 0.654425984878654 5.72522936244566e-52 postive

ATRX FAM13A-AS1 0.58166478682693 7.57066611485169e-39 postive

TSC1 ITFG1-AS1 0.400207290534928 2.3467683001943e-17 postive

MAPK8 ITFG1-AS1 0.477506408313218 5.74773626930034e-25 postive

BRAF ITFG1-AS1 0.614591883070771 2.31502540652592e-44 postive

ATRX ITFG1-AS1 0.624183499772819 4.29675462852084e-46 postive

TSC1 ZDHHC20-IT1 0.522226276496805 2.4216928628561e-30 postive

MAPK8 ZDHHC20-IT1 0.438378442464396 7.16743670185019e-21 postive

BRAF ZDHHC20-IT1 0.755420148940106 1.18144963512071e-77 postive

ATRX ZDHHC20-IT1 0.591782569532144 1.78587144629009e-40 postive

TSC1 AC010615.2 0.432867554266783 2.45973314335284e-20 postive

BRAF AC010615.2 0.51831850495013 7.693559116888e-30 postive

FASLG LINC02195 0.66020876280442 3.60100666233782e-53 postive

MLKL LINC02195 0.482018240887391 1.78803425120658e-25 postive

ZBP1 LINC02195 0.490955020612161 1.68038041155223e-26 postive

MAPK8 TTC3-AS1 0.419370614479166 4.58964033934203e-19 postive

OTULIN TTC3-AS1 0.413725576188601 1.50170618252518e-18 postive

BRAF TTC3-AS1 0.653622638150579 8.36763791207958e-52 postive

ATRX TTC3-AS1 0.534130928577447 6.51642513085513e-32 postive

LEF1 AC004034.1 0.487989212892679 3.71172505455106e-26 postive

BRAF AL731567.1 0.499789825529996 1.51365609820364e-27 postive

MAP3K7 LINC01140 0.421860761429292 2.7013514695761e-19 postive

IPMK AC087286.2 0.422907670898531 2.15888104054754e-19 postive

OTULIN AC087286.2 0.451990031545881 3.09055086382864e-22 postive

BRAF AC087286.2 0.552023761737731 2.1552500189385e-34 postive

ATRX AC087286.2 0.670148654646514 2.6785341929309e-55 postive

TSC1 AL731566.1 0.515631143311199 1.68889887627934e-29 postive

MAPK8 AL731566.1 0.500582822652812 1.21534365465362e-27 postive

OTULIN AL731566.1 0.487017073525601 4.80462096404868e-26 postive

BRAF AL731566.1 0.761572400874526 1.26679720829803e-79 postive

SIRT1 AL731566.1 0.401595904222442 1.7796053437441e-17 postive

ATRX AL731566.1 0.656498367608892 2.13945193199911e-52 postive

TSC1 AC073517.1 0.431800436564523 3.11500932104693e-20 postive

MAPK8 AC073517.1 0.472752882157039 1.93066024702036e-24 postive

OTULIN AC073517.1 0.446996727033009 9.95498371144595e-22 postive

BRAF AC073517.1 0.712358204744064 2.42392262877094e-65 postive

ATRX AC073517.1 0.59024596503725 3.18191017497893e-40 postive

TSC1 AC022154.1 0.448907772438359 6.37682589313126e-22 postive

BRAF AC022154.1 0.423581003846262 1.86825220754545e-19 postive

ATRX AC022154.1 0.581325808830462 8.56390663727469e-39 postive

MAP3K7 AL035530.2 0.432088104673721 2.92310526143358e-20 postive

ATRX AL035530.2 0.440120888422121 4.83054073230068e-21 postive

TSC1 AL139041.1 0.513555561621985 3.08511492609331e-29 postive

BRAF AL139041.1 0.727257147607472 2.46801668478947e-69 postive

FLT3 AC009041.2 0.468835106328021 5.1672144519261e-24 postive

RIPK3 AC106782.2 0.406421320040268 6.73613923936292e-18 postive

TSC1 AC020978.7 0.423480307041657 1.9091285849713e-19 postive

BRAF AC020978.7 0.49872112759934 2.03284607896018e-27 postive

ATRX AC020978.7 0.478444613007339 4.51505725175625e-25 postive

OTULIN GRK5-IT1 0.429298733160967 5.40135684194566e-20 postive

BRAF GRK5-IT1 0.470039485337499 3.82302837131741e-24 postive

ATRX GRK5-IT1 0.646610699409608 2.18810899858162e-50 postive

TSC1 NDUFB2-AS1 0.451442780641356 3.51658372375823e-22 postive

BRAF NDUFB2-AS1 0.664856070930971 3.72773060585188e-54 postive

TSC1 AL158152.1 0.520497039165546 4.04637782622298e-30 postive

MAPK8 AL158152.1 0.469410552219013 4.47509458313638e-24 postive

IPMK AL158152.1 0.417903328853686 6.2593012362756e-19 postive

OTULIN AL158152.1 0.511518119777025 5.55123253284354e-29 postive

BRAF AL158152.1 0.807008447546947 2.76361350888207e-96 postive

ATRX AL158152.1 0.652207055224538 1.62853134571996e-51 postive

MAP3K7 AC109449.1 0.403015954876966 1.33928511137161e-17 postive

TSC1 AC078795.1 0.514336541652865 2.46050549742618e-29 postive

MAPK8 AC078795.1 0.471908059135661 2.38983940907523e-24 postive

IPMK AC078795.1 0.412888843973878 1.78676437585127e-18 postive

OTULIN AC078795.1 0.475227305616723 1.02995232506387e-24 postive

BRAF AC078795.1 0.728848707296289 8.91693185627989e-70 postive

ATRX AC078795.1 0.708458611284503 2.44142109577116e-64 postive

ATRX AC009716.1 0.615258551871379 1.76252690073281e-44 postive

TSC1 AC073487.1 0.558017867698477 2.94159039882469e-35 postive

MAPK8 AC073487.1 0.424447069101407 1.55046204388776e-19 postive

OTULIN AC073487.1 0.413175751395026 1.68348391330574e-18 postive

BRAF AC073487.1 0.648717273853748 8.28237560445744e-51 postive

ATRX AC073487.1 0.666819000547137 1.41274987113795e-54 postive

TSC1 AC008735.2 0.409230896930305 3.79819663149777e-18 postive

GATA3 AC008735.2 0.403175702387843 1.29702543926517e-17 postive

MAPK8 KCNIP2-AS1 0.426905400914094 9.10622398051937e-20 postive

BRAF KCNIP2-AS1 0.609334414590026 1.9436273663605e-43 postive

ATRX KCNIP2-AS1 0.43388588463205 1.96185431844022e-20 postive

TSC1 CDC42-IT1 0.438929825517415 6.32765345339923e-21 postive

MAPK8 CDC42-IT1 0.470892277167818 3.08633115459469e-24 postive

IPMK CDC42-IT1 0.410057369247654 3.20576334918937e-18 postive

OTULIN CDC42-IT1 0.510663782632452 7.09340659727366e-29 postive

BRAF CDC42-IT1 0.782635629622417 7.5805523301147e-87 postive

ATRX CDC42-IT1 0.638410630232796 8.93570483996522e-49 postive

BRAF AC105206.2 0.447954073422747 7.96692144462222e-22 postive

ATRX AC105206.2 0.450566692022219 4.32211643208054e-22 postive

TSC1 AC139887.2 0.591432502990668 2.03757616099061e-40 postive

MAPK8 AC139887.2 0.493113237739187 9.39369166422154e-27 postive

OTULIN AC139887.2 0.458684095002983 6.24800709078711e-23 postive

BRAF AC139887.2 0.744135362321897 3.44642104128925e-74 postive

SIRT1 AC139887.2 0.403726440416266 1.16114193877034e-17 postive

ATRX AC139887.2 0.678137328263904 4.5320780018998e-57 postive

ATRX AC138207.4 0.552234652831391 2.01077476908542e-34 postive

MAPK8 AL049539.1 0.403544304513754 1.20445418394877e-17 postive

OTULIN AL049539.1 0.46829857603278 5.90718749092635e-24 postive

BRAF AL049539.1 0.594953327642789 5.37005593190111e-41 postive

ATRX AL049539.1 0.500813673754051 1.13999005718956e-27 postive

TSC1 AP001001.1 0.441062639246852 3.89908807896541e-21 postive

IPMK AP001001.1 0.408946139943983 4.02628467441263e-18 postive

BRAF AP001001.1 0.613707951257399 3.31995890833622e-44 postive

ATRX AP001001.1 0.658495798434732 8.22362351149424e-53 postive

TSC1 AC004596.1 0.436386478479977 1.12218431038628e-20 postive

BRAF AC004596.1 0.522571193718194 2.18522580418261e-30 postive

ATRX AC004596.1 0.489156453602325 2.71971653392793e-26 postive

TSC1 AL513327.1 0.488649623244789 3.11335524728884e-26 postive

MAPK8 AL513327.1 0.437969270931112 7.86072125944888e-21 postive

OTULIN AL513327.1 0.457971378603731 7.41982472853145e-23 postive

BRAF AL513327.1 0.609632174559334 1.72480525477308e-43 postive

ATRX AL513327.1 0.674105188075892 3.60951360457528e-56 postive

TSC1 AL354733.3 0.656351156248424 2.2949895192922e-52 postive

BRAF AL354733.3 0.425183653434384 1.32253976316029e-19 postive

ATRX AL354733.3 0.481845665577031 1.87030173629652e-25 postive

TSC1 AC012615.6 0.487870463627177 3.83077185185649e-26 postive

ATRX AC012615.6 0.450218527323769 4.69053795820689e-22 postive

RNF31 AL096870.2 0.475293204247827 1.01278945573293e-24 postive

TSC1 GAS8-AS1 0.434762797950568 1.61368712181638e-20 postive

BRAF GAS8-AS1 0.480524141185625 2.63720376245528e-25 postive

ATRX GAS8-AS1 0.507841364510158 1.58653248899563e-28 postive

TSC1 AP001628.1 0.435264079870694 1.44279916414347e-20 postive

MAPK8 AP001628.1 0.469974399143588 3.88590363399003e-24 postive

OTULIN AP001628.1 0.458908733246937 5.9180121967859e-23 postive

BRAF AP001628.1 0.817825373322474 6.44613840140667e-101 postive

ATRX AP001628.1 0.533223308055487 8.62818545778563e-32 postive

ATRX AC092614.1 0.441228213547282 3.75471066378077e-21 postive

TRIM11 AL353622.2 0.414072907713556 1.39697837107667e-18 postive

ATRX ATP1B3-AS1 0.52573701045129 8.46314209604152e-31 postive

TSC1 AC066613.1 0.461220571679127 3.37782319301357e-23 postive

BRAF AC066613.1 0.545047318198719 2.08159761841395e-33 postive

ATRX AC066613.1 0.629164895727959 5.12777098675725e-47 postive

TSC1 SPAG5-AS1 0.556461840687572 4.9521593376211e-35 postive

MAPK8 SPAG5-AS1 0.483682877108853 1.15708568899734e-25 postive

OTULIN SPAG5-AS1 0.41630437503034 8.76221895416079e-19 postive

BRAF SPAG5-AS1 0.7910854598671 5.6348777309389e-90 postive

ATRX SPAG5-AS1 0.605317360238923 9.6180750198482e-43 postive

TSC1 AC010976.1 0.474576339531358 1.21569188057203e-24 postive

MAPK8 AC010976.1 0.485015989774854 8.15173160104648e-26 postive

IPMK AC010976.1 0.410997384159513 2.6419159249123e-18 postive

OTULIN AC010976.1 0.507374322472941 1.81127648635016e-28 postive

BRAF AC010976.1 0.801879307114803 3.44016177883074e-94 postive

ATRX AC010976.1 0.64485121471267 4.89687899951126e-50 postive

TSC1 U47924.1 0.4207186938063 3.44663447099422e-19 postive

BRAF U47924.1 0.659825731906383 4.33334011860705e-53 postive

TSC1 AC007622.2 0.478456652779357 4.50107088618948e-25 postive

MAPK8 AC007622.2 0.503388167990077 5.56486035179518e-28 postive

IPMK AC007622.2 0.441228773512469 3.75423143824336e-21 postive

OTULIN AC007622.2 0.510959735540993 6.51640314100521e-29 postive

BRAF AC007622.2 0.804386812035831 3.31218277423094e-95 postive

ATRX AC007622.2 0.693025395797852 1.58536983115703e-60 postive

AXL LINC01119 0.411409351215379 2.42670176696301e-18 postive

KLF9 LINC01119 0.431421195424724 3.38707315858259e-20 postive

TSC1 AL139289.1 0.45455673059366 1.68125089019657e-22 postive

BRAF AL139289.1 0.408926151428833 4.04279201100327e-18 postive

TSC1 GSN-AS1 0.417873545137611 6.29874728742482e-19 postive

IPMK GSN-AS1 0.422374569439767 2.42016193334329e-19 postive

OTULIN GSN-AS1 0.475504367329249 9.59672910323131e-25 postive

BRAF GSN-AS1 0.602370056093946 3.06459759398655e-42 postive

ATRX GSN-AS1 0.687219596446546 3.73821384468837e-59 postive

KLF9 LINC01352 0.444176858453263 1.91093882366248e-21 postive

KLF9 AC053503.4 0.442684632714164 2.69184540947115e-21 postive

TNFRSF1B CYTOR 0.443680618334718 2.14196747256972e-21 postive

BRAF AC024560.3 0.550983003440895 3.03318082127292e-34 postive

TRIM11 AC097347.1 0.409652885459892 3.4833738314205e-18 postive

TSC1 AC067750.1 0.455767263024772 1.25937002789242e-22 postive

MAPK8 AC067750.1 0.462930241463476 2.22503461378739e-23 postive

BRAF AC067750.1 0.666729654990225 1.47677643346144e-54 postive

ATRX AC067750.1 0.508638677746526 1.26479804423245e-28 postive

TSC1 AL513327.2 0.41437378798152 1.3120898540537e-18 postive

MAPK8 AL513327.2 0.448425989682805 7.13650752806406e-22 postive

BRAF AL513327.2 0.673158236506421 5.84808063280537e-56 postive

ATRX AL513327.2 0.508900569053612 1.1739086124323e-28 postive

BRAF DSG2-AS1 0.488058838873944 3.64363166446268e-26 postive

FASLG LINC01943 0.519935019793471 4.77806095050958e-30 postive

TNFRSF1B LINC01943 0.615806656787541 1.40787458648317e-44 postive

MAPK8 AC015849.4 0.404744570802586 9.45795397710174e-18 postive

OTULIN AC015849.4 0.428851234145793 5.95735257385448e-20 postive

BRAF AC015849.4 0.623736095892492 5.19090971747788e-46 postive

ATRX AC015849.4 0.619450250630601 3.12634716420078e-45 postive

MAPK8 AC104984.2 0.403601896238228 1.1905897998909e-17 postive

OTULIN AC104984.2 0.4440698899496 1.95856515451699e-21 postive

BRAF AC104984.2 0.600464746158358 6.44095720974095e-42 postive

ATRX AC104984.2 0.6281719087617 7.85778641167237e-47 postive

TSC1 AC109460.2 0.543548199481419 3.36540866519927e-33 postive

BRAF AC109460.2 0.46227744674704 2.61025035380426e-23 postive

ATRX AC109460.2 0.495624842074741 4.74941934696601e-27 postive

GATA3 AC026801.2 0.412019623820654 2.13922495063037e-18 postive

TSC1 RUSC1-AS1 0.452217188659148 2.92904419583426e-22 postive

BRAF ADNP-AS1 0.444119595483512 1.9362906462101e-21 postive

ATRX ADNP-AS1 0.412663929246935 1.87206513197818e-18 postive

ID1 KRT7-AS 0.424863794581751 1.41714824689376e-19 postive

TSC1 Z82243.1 0.419947435920645 4.06093006262827e-19 postive

OTULIN Z82243.1 0.455655154967721 1.29358536790075e-22 postive

BRAF Z82243.1 0.510243602975349 8.0003033601065e-29 postive

ATRX Z82243.1 0.635022632368566 4.00478094664901e-48 postive

TSC1 AC068790.2 0.429338287140642 5.35474550921384e-20 postive

MAPK8 AC068790.2 0.49195914178416 1.28268716197476e-26 postive

IPMK AC068790.2 0.41856418613742 5.4440013931428e-19 postive

OTULIN AC068790.2 0.514470099653915 2.36699510153247e-29 postive

BRAF AC068790.2 0.755372107858553 1.22339117833007e-77 postive

ATRX AC068790.2 0.642839652209625 1.22202900100493e-49 postive

BRAF AP003559.1 0.402570649155422 1.46436367203495e-17 postive

TSC1 AC006064.2 0.414116216987981 1.38443444368058e-18 postive

MAPK8 AC006064.2 0.449900298200521 5.05427169268422e-22 postive

IPMK AC006064.2 0.407915379001977 4.97027749808075e-18 postive

OTULIN AC006064.2 0.549990579434745 4.1968471319733e-34 postive

BRAF AC006064.2 0.686351198126226 5.959271447277e-59 postive

ATRX AC006064.2 0.630192372294382 3.29161675638894e-47 postive

TSC1 MED4-AS1 0.420554501176506 3.56923192615567e-19 postive

MAPK8 MED4-AS1 0.409480530253104 3.60873056979862e-18 postive

OTULIN MED4-AS1 0.428204578474066 6.86165399679708e-20 postive

BRAF MED4-AS1 0.751758857163229 1.64835663584494e-76 postive

ATRX MED4-AS1 0.54961902925609 4.73805197401344e-34 postive

BRAF AC253536.6 0.556093706687833 5.5993719160654e-35 postive

ATRX AC253536.6 0.426444572373186 1.00648617549291e-19 postive

MAP3K7 FGF12-AS3 0.495789687740564 4.54061118573324e-27 postive

BRAF FGF12-AS3 0.535473236207517 4.29570897290458e-32 postive

BRAF TUG1 0.419960908345513 4.04932676787944e-19 postive

TSC1 AP001020.3 0.488049111770772 3.65306999013911e-26 postive

MAPK8 AP001020.3 0.465381268745351 1.217962059571e-23 postive

IPMK AP001020.3 0.403985297714976 1.10220872894763e-17 postive

OTULIN AP001020.3 0.514420508763721 2.40129870300146e-29 postive

BRAF AP001020.3 0.815785657580964 5.08228043085049e-100 postive

ATRX AP001020.3 0.646402727784905 2.40736362362391e-50 postive

TSC1 AC004771.2 0.419508688425613 4.45722633545507e-19 postive

BRAF AC004771.2 0.629194878172359 5.06198686293269e-47 postive

ATRX AC004771.2 0.547480311005859 9.49598806767839e-34 postive

TSC1 AC139887.1 0.534109862066058 6.55908432165536e-32 postive

MAPK8 AC139887.1 0.440486572719309 4.44534362603886e-21 postive

BRAF AC139887.1 0.695143428995604 4.9119395152266e-61 postive

ATRX AC139887.1 0.604215295819595 1.4855669488451e-42 postive

OTULIN AL356055.1 0.46606203657517 1.02936856182471e-23 postive

BRAF AL356055.1 0.567618935865148 1.11190519904179e-36 postive

ATRX AL356055.1 0.556103760713328 5.58063105110576e-35 postive

TSC1 AL138921.1 0.455559849918129 1.32339217762161e-22 postive

MAPK8 AL138921.1 0.405055647273335 8.88211625536974e-18 postive

BRAF AL138921.1 0.606353171804752 6.38206798685841e-43 postive

ATRX AL138921.1 0.467018079977361 8.12246021902245e-24 postive

TSC1 AC137932.3 0.451445135318178 3.51463200254035e-22 postive

BRAF AC137932.3 0.472497315404746 2.05951231725355e-24 postive

ATRX AC137932.3 0.499895784710222 1.46995493731882e-27 postive

TSC1 AC211476.2 0.446039391107604 1.24303711050391e-21 postive

MAPK8 AC211476.2 0.45413651824291 1.85811758547025e-22 postive

OTULIN AC211476.2 0.44559617198759 1.37730938051013e-21 postive

BRAF AC211476.2 0.683055304618258 3.44704498118592e-58 postive

ATRX AC211476.2 0.600152439215964 7.27141622034161e-42 postive

TSC1 AC145423.3 0.444495851721601 1.77558609309256e-21 postive

BRAF AC145423.3 0.565249992226724 2.51989599525403e-36 postive

ATRX AC145423.3 0.490722034978094 1.78881839048944e-26 postive

BRAF AC012184.3 0.407248291674682 5.69394612233775e-18 postive

TSC1 AC009095.1 0.493298713687782 8.93401379786719e-27 postive

MAPK8 AC009095.1 0.460697001753906 3.83667069506529e-23 postive

OTULIN AC009095.1 0.453072785729844 2.39205399801173e-22 postive

BRAF AC009095.1 0.685269000380274 1.06314644727673e-58 postive

ATRX AC009095.1 0.693134182413122 1.49314212604223e-60 postive

MAP3K7 AC021683.1 0.435890721788597 1.25406917043559e-20 postive

KLF9 AL445423.1 0.43821168486959 7.44239227432289e-21 postive

FLT3 PCAT19 0.435327224370762 1.42257936752184e-20 postive

KLF9 PCAT19 0.606477435649293 6.07502429162577e-43 postive

TSC1 Z94721.1 0.581968456192008 6.7782818231567e-39 postive

MAPK8 Z94721.1 0.413731997499207 1.49970168045459e-18 postive

BRAF Z94721.1 0.5756861285378 6.5204998230185e-38 postive

ATRX Z94721.1 0.546347394320803 1.3696378338668e-33 postive

FLT3 AC010976.2 0.423412551513139 1.9371271776364e-19 postive

KLF9 AC010976.2 0.450039518273131 4.89184381877563e-22 postive

SIRT3 AC132192.2 0.500483338866816 1.24931501563522e-27 postive

TSC1 AL117329.1 0.414054685897785 1.40228942771805e-18 postive

MAPK8 AL117329.1 0.485666532329275 6.86715392265466e-26 postive

IPMK AL117329.1 0.402176237556242 1.58469069905937e-17 postive

OTULIN AL117329.1 0.484477522303836 9.39230848959114e-26 postive

BRAF AL117329.1 0.792167080100768 2.1873317333546e-90 postive

ATRX AL117329.1 0.619098430093637 3.61838906305806e-45 postive

TSC1 AC012360.3 0.52218750885849 2.44980394240213e-30 postive

TSC1 AC127024.4 0.527727517474181 4.63747248429599e-31 postive

MAPK8 AC127024.4 0.413533647232403 1.56285164568108e-18 postive

BRAF AC127024.4 0.610661659295831 1.14016713189607e-43 postive

ATRX AC127024.4 0.655924847091085 2.81155104687324e-52 postive

MAPK8 AC087276.2 0.405746737064563 7.72337177087054e-18 postive

BRAF AC087276.2 0.571821140260937 2.56255989104424e-37 postive

ATRX AC087276.2 0.643992174085666 7.24257765264976e-50 postive

FAS DANCR -0.42050138750084 3.60980154168435e-19 negative

STAT3 DANCR -0.426746238953495 9.42669487926588e-20 negative

BRAF AC233280.1 0.414297926872661 1.3330016823798e-18 postive

TSC1 LINC01876 0.425881127965274 1.13727746580704e-19 postive

MAPK8 LINC01876 0.46956514652562 4.30530218093628e-24 postive

BRAF LINC01876 0.807631609239975 1.52281373232291e-96 postive

ATRX LINC01876 0.52427589821755 1.31281340446932e-30 postive

IPMK KLF7-IT1 0.428603946063605 6.28839109217802e-20 postive

OTULIN KLF7-IT1 0.452493548098238 2.74374310272202e-22 postive

BRAF KLF7-IT1 0.532373139348188 1.12144637707456e-31 postive

ATRX KLF7-IT1 0.672279629194384 9.13600873981151e-56 postive

TSC1 LINC01336 0.478319223324929 4.66329454754839e-25 postive

MAPK8 LINC01336 0.486566177566842 5.41406987809049e-26 postive

BRAF LINC01336 0.87576834283419 2.17914193497307e-132 postive

ATRX LINC01336 0.526898061047849 5.96150725274043e-31 postive

TSC1 MAL2-AS1 0.41618927093818 8.9763640561757e-19 postive

MAPK8 MAL2-AS1 0.464355920447358 1.56808633274113e-23 postive

OTULIN MAL2-AS1 0.480593703150535 2.59002897504498e-25 postive

BRAF MAL2-AS1 0.841765799920574 2.30550908542359e-112 postive

ATRX MAL2-AS1 0.520843892875646 3.65131822843362e-30 postive

TSC1 C21orf62-AS1 0.508976866533245 1.14866571478643e-28 postive

MAPK8 C21orf62-AS1 0.502390565216148 7.35278910571796e-28 postive

BRAF C21orf62-AS1 0.723083976479398 3.44286747667923e-68 postive

ATRX C21orf62-AS1 0.514254859499001 2.51948522220567e-29 postive

KLF9 AL139393.2 0.615863789109636 1.37525243598668e-44 postive

MAPK8 AC073529.1 0.483259485713679 1.29281823000836e-25 postive

OTULIN AC073529.1 0.505594791820047 2.99500321256916e-28 postive

DIABLO AC073529.1 0.420117842102303 3.91654625962028e-19 postive

BRAF AC073529.1 0.636485998350129 2.09990713477225e-48 postive

ATRX AC073529.1 0.602364188882127 3.07163869961089e-42 postive

BRAF AC022079.2 0.515132968945139 1.95241567378687e-29 postive

TSC1 MIR29B2CHG 0.489637288798301 2.39187781671011e-26 postive

BRAF MIR29B2CHG 0.607420886344306 4.17505638241414e-43 postive

ATRX MIR29B2CHG 0.49053729500639 1.87968986353596e-26 postive

BRAF AC127024.2 0.495896241312499 4.41049416006066e-27 postive

ATRX AC127024.2 0.56194489846587 7.8045356524825e-36 postive

FASLG U62317.2 0.425257077467371 1.30171582014511e-19 postive

MLKL U62317.2 0.483573566314698 1.19071804336695e-25 postive

ZBP1 U62317.2 0.402046124446435 1.62647846387959e-17 postive

DDX58 U62317.2 0.442192682941701 3.01263258050024e-21 postive

BRAF AC007881.3 0.529812884870404 2.45877792761785e-31 postive

TRIM11 AP005329.1 0.431513367275057 3.31887182863085e-20 postive

MAPK8 MORC2-AS1 0.438421165581273 7.09861947159583e-21 postive

OTULIN MORC2-AS1 0.456254530030933 1.12073678226632e-22 postive

BRAF MORC2-AS1 0.736404485883963 6.42017523808167e-72 postive

ATRX MORC2-AS1 0.488516601932702 3.22567070058613e-26 postive

AXL AL596244.1 0.604914047487109 1.1278947298171e-42 postive

DDX58 AL596244.1 0.471902512739749 2.39318468197031e-24 postive

TRIM11 AP002360.3 0.446086690563005 1.2294934829537e-21 postive

MAP3K7 COX10-AS1 0.436047848758642 1.21069633115459e-20 postive

ATRX COX10-AS1 0.463946975569957 1.73393743790155e-23 postive

TSC1 AC012557.1 0.413003404690549 1.74479806275779e-18 postive

MAPK8 AC012557.1 0.444539762444749 1.75771006784155e-21 postive

IPMK AC012557.1 0.400364187066079 2.27469592145585e-17 postive

OTULIN AC012557.1 0.497477935992867 2.86106313794969e-27 postive

BRAF AC012557.1 0.740305024265991 4.70190910717215e-73 postive

SIRT1 AC012557.1 0.401149453481613 1.94541964410144e-17 postive

ATRX AC012557.1 0.629227508495903 4.99134370676331e-47 postive

BRAF AC015853.1 0.460629228929957 3.90039033668774e-23 postive

ATRX AC015853.1 0.51466712642109 2.23542634514386e-29 postive

TSC1 PSPC1-AS2 0.509131037807342 1.09928446486282e-28 postive

MAPK8 PSPC1-AS2 0.420381329417588 3.70318505938983e-19 postive

BRAF PSPC1-AS2 0.694312565444289 7.78759779987547e-61 postive

ATRX PSPC1-AS2 0.521220586646433 3.26539978422365e-30 postive

MAPK8 AC004884.2 0.450226318042422 4.68196484241826e-22 postive

IPMK AC004884.2 0.422079534327963 2.57789621793417e-19 postive

OTULIN AC004884.2 0.510821062777371 6.78074719317868e-29 postive

BRAF AC004884.2 0.704285578905877 2.7725770092533e-63 postive

ATRX AC004884.2 0.643448980420936 9.27023587055485e-50 postive

BRAF AP006545.1 0.48782568486235 3.87663524239789e-26 postive

STAT3 AC137767.1 -0.420091542391122 3.93849541715194e-19 negative

TSC1 AL080317.2 0.452184198034742 2.95197297454147e-22 postive

MAPK8 AL080317.2 0.44629360673747 1.17193558558282e-21 postive

OTULIN AL080317.2 0.451064589535151 3.84433119057818e-22 postive

BRAF AL080317.2 0.816088231322101 3.74746956303901e-100 postive

ATRX AL080317.2 0.514561815920586 2.30482371578937e-29 postive

FLT3 AC027449.1 0.454523995576756 1.69441101535352e-22 postive

KLF9 AC027449.1 0.550816505436846 3.20324992368485e-34 postive

TSC1 AL139353.2 0.432588904367291 2.61641652161918e-20 postive

MAPK8 AL139353.2 0.424318092266611 1.59417090559508e-19 postive

OTULIN AL139353.2 0.448032342993509 7.82288652195657e-22 postive

BRAF AL139353.2 0.671375962676665 1.44321063329848e-55 postive

ATRX AL139353.2 0.597208995002583 2.26558653873745e-41 postive

ATRX AC010525.1 0.417524338280652 6.77989474446318e-19 postive

BRAF AL138831.2 0.405372388396123 8.33123793026371e-18 postive

BRAF AC095055.1 0.547821212250061 8.50271712353659e-34 postive

TSC1 LINC01126 0.567126963095709 1.31854313211898e-36 postive

MAPK8 LINC01126 0.416662191981368 8.12811825617379e-19 postive

OTULIN LINC01126 0.42384645030056 1.76458234889188e-19 postive

BRAF LINC01126 0.681685991476491 7.09870250782692e-58 postive

ATRX LINC01126 0.533559222403146 7.77755395336624e-32 postive

BRAF AC007285.1 0.400073912002612 2.409799009195e-17 postive

ATRX AC007285.1 0.544188692119084 2.74173272762144e-33 postive

TSC1 AC022274.1 0.401045463164968 1.98617157744125e-17 postive

MAPK8 AC022274.1 0.402304487870231 1.54453471565961e-17 postive

OTULIN AC022274.1 0.423987421351268 1.71185056530629e-19 postive

BRAF AC022274.1 0.773726509636457 1.07183657660339e-83 postive

ATRX AC022274.1 0.432806847395132 2.49306091112457e-20 postive

MAP3K7 AC005330.1 0.413175619609902 1.6835299775708e-18 postive

ATRX AC090673.1 0.424878810804535 1.41256128631848e-19 postive

GATA3 ZNF32-AS1 0.402395506422816 1.51664486009863e-17 postive

TSC1 AC106037.1 0.533586549559706 7.71211988822766e-32 postive

MAPK8 AC106037.1 0.40790475088373 4.98106480424534e-18 postive

OTULIN AC106037.1 0.511341942533182 5.8394079335754e-29 postive

BRAF AC106037.1 0.712837817861433 1.81964283361545e-65 postive

ATRX AC106037.1 0.630241982703145 3.22177636757228e-47 postive

TSC1 AC011477.3 0.472706792727398 1.95329452126846e-24 postive

BRAF AC011477.3 0.5254196337119 9.31166186402952e-31 postive

SIRT1 AC011477.3 0.408238557421728 4.65300879472537e-18 postive

GATA3 AC011477.3 0.408182308884957 4.70675964515901e-18 postive

IDH1 AC011477.3 0.496380565779149 3.86399723361142e-27 postive

ATRX RBMS3-AS3 0.403402392947213 1.23929845188596e-17 postive

OTULIN LINC-PINT 0.448300255807542 7.34902571074008e-22 postive

CFLAR LINC-PINT 0.432255750706767 2.81668961032267e-20 postive

BRAF LINC-PINT 0.473989222729893 1.411351395845e-24 postive

ATRX LINC-PINT 0.598780338945919 1.23688829315747e-41 postive

BRAF AC092140.2 0.427543417130808 7.92583045034839e-20 postive

ATRX AC092140.2 0.409777866435034 3.39516209172748e-18 postive

OTULIN AL133243.1 0.448853640237276 6.45803854838352e-22 postive

BRAF AL133243.1 0.568580396401329 7.96230408255413e-37 postive

ATRX AL133243.1 0.564703214130585 3.0407922491077e-36 postive

TSC1 FBXL19-AS1 0.515299155082802 1.86027905974601e-29 postive

BRAF FBXL19-AS1 0.465650898300915 1.13950322610528e-23 postive

FADD AP002336.2 0.444957172757451 1.59640036089119e-21 postive

MAPK8 AL163051.2 0.408714796058698 4.22146286780435e-18 postive

OTULIN AL163051.2 0.403302820692245 1.26433668998026e-17 postive

BRAF AL163051.2 0.640307297772886 3.8269473038942e-49 postive

ATRX AL163051.2 0.584127949818139 3.07762287934329e-39 postive

BRAF LINC00664 0.423519051111808 1.89329783068447e-19 postive

BCL2 AL391807.1 0.41831367978387 5.73994953234883e-19 postive

KLF9 MBNL1-AS1 0.528120136495998 4.11667289370432e-31 postive

ATRX AC079313.1 0.436991172758348 9.79705158709265e-21 postive

TSC1 HDAC2-AS2 0.465372573196717 1.22057914248071e-23 postive

BRAF HDAC2-AS2 0.537223649974836 2.48780494227541e-32 postive

GATA3 HDAC2-AS2 0.439404703254228 5.68265966934302e-21 postive

ATRX HDAC2-AS2 0.405124557003933 8.75929891438842e-18 postive

IDH1 HDAC2-AS2 0.435048398421091 1.51402204816127e-20 postive

TSC1 ANKRD10-IT1 0.573420744584517 1.45756249000592e-37 postive

BRAF ANKRD10-IT1 0.490374369333042 1.96360529238466e-26 postive

ATRX ANKRD10-IT1 0.585479917468543 1.87167100607257e-39 postive

KLF9 AP001189.3 0.575951463858177 5.93178444870848e-38 postive

BRAF ABALON 0.404588730842861 9.76007781452254e-18 postive

ATRX ABALON 0.475087032773364 1.06744716526039e-24 postive

BRAF PRR7-AS1 0.442515963631043 2.79784401144017e-21 postive

ATRX PRR7-AS1 0.56895677515865 6.98449720063983e-37 postive

TSC1 AC245060.5 0.404437988915652 1.00613395594302e-17 postive

TSC1 AC099482.1 0.411378805910325 2.44204858898562e-18 postive

MAPK8 AC099482.1 0.412939323878051 1.76815139772133e-18 postive

OTULIN AC099482.1 0.420391891995329 3.69487477564349e-19 postive

BRAF AC099482.1 0.799924337477304 2.08441542608538e-93 postive

ATRX AC099482.1 0.445274299941454 1.48368465666116e-21 postive

TSC1 FMR1-IT1 0.53683699080405 2.80760710225794e-32 postive

BRAF FMR1-IT1 0.612041301491132 6.53152532580709e-44 postive

ATRX FMR1-IT1 0.620983859784498 1.64964080000244e-45 postive

TSC1 AC020978.2 0.468757194979533 5.26869760778121e-24 postive

BRAF AC020978.2 0.530584187913332 1.94228617839321e-31 postive

ATRX AC020978.2 0.60068188891808 5.91965651835513e-42 postive

TSC1 MKNK1-AS1 0.401313191991939 1.8829137576178e-17 postive

BRAF MKNK1-AS1 0.435458467525798 1.38144230446438e-20 postive

ATRX MKNK1-AS1 0.658114266685104 9.87696944402639e-53 postive

TSC1 AC008543.3 0.513234617378453 3.38510770655186e-29 postive

MAPK8 AC008543.3 0.466539224564825 9.14659442006308e-24 postive

OTULIN AC008543.3 0.438533405178694 6.92091370049592e-21 postive

BRAF AC008543.3 0.821560719500715 1.37250020375877e-102 postive

ATRX AC008543.3 0.565717287514182 2.1454601272793e-36 postive

TSC1 AC100821.2 0.488257140668638 3.45638292889942e-26 postive

BRAF AC100821.2 0.682485277722306 4.6586328717475e-58 postive

ATRX AC100821.2 0.470584648244226 3.33432557769353e-24 postive

TSC1 AC022272.1 0.464127371160703 1.65874508882647e-23 postive

MAPK8 AC022272.1 0.471345384192409 2.75386082292332e-24 postive

IPMK AC022272.1 0.421918105971768 2.66843976964555e-19 postive

OTULIN AC022272.1 0.510504501636465 7.42456206822435e-29 postive

BRAF AC022272.1 0.82226202227197 6.59590962812434e-103 postive

ATRX AC022272.1 0.648326134387178 9.92537350688481e-51 postive

TSC1 AL731569.1 0.45559067444701 1.31367839336265e-22 postive

MAPK8 AL731569.1 0.427070082953377 8.78593255402428e-20 postive

BRAF AL731569.1 0.5464049705101 1.34442455366399e-33 postive

TSC1 AL022311.1 0.409660659996334 3.47782140249269e-18 postive

BRAF AL022311.1 0.611421507931127 8.3918529871221e-44 postive

ATRX AL022311.1 0.524387369044699 1.26966436796237e-30 postive

OTULIN LRP4-AS1 0.425539930098464 1.22446991830192e-19 postive

BRAF LRP4-AS1 0.603950324158505 1.64883468685432e-42 postive

ATRX LRP4-AS1 0.444859465491439 1.63279923031684e-21 postive

TSC1 DNAJC3-DT 0.422396739236416 2.40870071500971e-19 postive

TSC1 AC018766.1 0.55671228092399 4.55476970015711e-35 postive

MAPK8 AC018766.1 0.420150643964389 3.88933922069921e-19 postive

BRAF AC018766.1 0.651608698153894 2.1556120752242e-51 postive

ATRX AC018766.1 0.52467252576007 1.16556261272502e-30 postive

TNFRSF1B AC017002.3 0.409062413076495 3.93156778594055e-18 postive

CFLAR AC017002.3 0.40534255795822 8.38165223662914e-18 postive

BRAF AC037459.2 0.446379334192504 1.14887453640367e-21 postive

ATRX AC037459.2 0.581621376223846 7.69119491532941e-39 postive

GATA3 WASIR2 0.460291470052525 4.23384886475706e-23 postive

TSC1 AL928654.2 0.447248040480008 9.39014421451021e-22 postive

BRAF AC009961.1 0.486246793439103 5.89134955426169e-26 postive

TSC1 SGMS1-AS1 0.461048418416934 3.52238023275726e-23 postive

MAPK8 SGMS1-AS1 0.400187160945143 2.35617604126537e-17 postive

IPMK SGMS1-AS1 0.419946731737583 4.06153744826891e-19 postive

BRAF SGMS1-AS1 0.545824744303949 1.62100316211252e-33 postive

ATRX SGMS1-AS1 0.591678771172729 1.85710626265615e-40 postive

BRAF AL390195.2 0.609458455321395 1.8493132320258e-43 postive

ATRX AL390195.2 0.431981812503624 2.99261684514609e-20 postive

PLK1 AC026401.3 0.437453211736669 8.82995336901569e-21 postive

LEF1 AL158166.1 0.517063959993382 1.11152038401142e-29 postive

TSC1 LAMTOR5-AS1 0.519615701831773 5.2505489898049e-30 postive

MAPK8 LAMTOR5-AS1 0.527640118449628 4.76199166342301e-31 postive

IPMK LAMTOR5-AS1 0.411914004705284 2.18646090154864e-18 postive

OTULIN LAMTOR5-AS1 0.524233585395872 1.32956918530708e-30 postive

BRAF LAMTOR5-AS1 0.800786919511664 9.43719300642569e-94 postive

ATRX LAMTOR5-AS1 0.620695535134088 1.8608255319141e-45 postive

MAPK8 AC092127.2 0.536555899084385 3.0653108406953e-32 postive

BRAF AC092127.2 0.483778621364479 1.12839923433276e-25 postive

TSC1 BMS1P4 0.406854878601063 6.1683029577856e-18 postive

MAPK8 BMS1P4 0.404963805113019 9.04844164875926e-18 postive

OTULIN BMS1P4 0.401387442491519 1.85522393134685e-17 postive

BRAF BMS1P4 0.624128365658806 4.39810297356489e-46 postive

ATRX BMS1P4 0.497934693813482 2.52386654403592e-27 postive

BRAF LINC02340 0.421182336050822 3.12238716993102e-19 postive

TSC1 AL162741.1 0.400635392859869 2.15521086958603e-17 postive

BRAF AL162741.1 0.44571638246673 1.33954017568423e-21 postive

TSC1 AC078846.1 0.46557146491441 1.16208416416446e-23 postive

MAPK8 AC078846.1 0.434701259140917 1.63599394805552e-20 postive

BRAF AC078846.1 0.688560149113759 1.81391982171633e-59 postive

ATRX AC078846.1 0.5371394742539 2.55420477632456e-32 postive

SLC39A7 LINC01213 0.425435411165986 1.25247306041382e-19 postive

TRIM11 AC005387.1 0.436350503472598 1.13127631185823e-20 postive

MAPK8 Z97989.1 0.439337910658491 5.76930211237192e-21 postive

BRAF Z97989.1 0.451006923557377 3.89688368268784e-22 postive

TSC1 GHRLOS 0.551066994366308 2.95081118167896e-34 postive

BRAF GHRLOS 0.618668242848591 4.32537293687271e-45 postive

ATRX GHRLOS 0.403006983587876 1.34169811444148e-17 postive

BRAF AC105339.2 0.548018575841755 7.97541559096758e-34 postive

ATRX AC105339.2 0.567807967733381 1.04133701056481e-36 postive

TSC1 AF230666.1 0.461637644299177 3.0514267450995e-23 postive

MAPK8 AF230666.1 0.429910174514898 4.72347445324731e-20 postive

OTULIN AF230666.1 0.435681830622303 1.31410893133209e-20 postive

BRAF AF230666.1 0.703147748715795 5.33833104326988e-63 postive

ATRX AF230666.1 0.605028389700803 1.07811585032335e-42 postive

TSC1 N4BP2L2-IT2 0.492514001063441 1.10444389381232e-26 postive

MAPK8 N4BP2L2-IT2 0.445834730586347 1.30335397030788e-21 postive

OTULIN N4BP2L2-IT2 0.478052900804191 4.99426892994965e-25 postive

BRAF N4BP2L2-IT2 0.794538631913575 2.69276099007721e-91 postive

ATRX N4BP2L2-IT2 0.616598062481844 1.01704840297538e-44 postive

TSC1 AL021707.4 0.536305485294821 3.31451674736097e-32 postive

BRAF AL021707.4 0.665678547066337 2.48470641051101e-54 postive

ATRX AL021707.4 0.561223129583303 9.97315849968841e-36 postive

TSC1 AP001793.1 0.460615176795531 3.91373215791868e-23 postive

BRAF AP001793.1 0.495278056938694 5.22015160461219e-27 postive

ATRX AP001793.1 0.558445090921369 2.54838188554445e-35 postive

TNFRSF1B AL133371.2 0.588330021153274 6.50991361897242e-40 postive

CYLD AL133371.2 0.407068051334292 5.90664249049983e-18 postive

FAS AL357054.4 0.405077163364319 8.84358756599612e-18 postive

FASLG AL357054.4 0.439805723992256 5.18877913226734e-21 postive

MLKL AL357054.4 0.456065219383079 1.17271229309475e-22 postive

CYLD AL357054.4 0.45640570538261 1.08086867271843e-22 postive

CYLD XXYLT1-AS2 0.442422756846375 2.85817279095724e-21 postive

TSC1 AC245884.1 0.464681783451601 1.44722199175602e-23 postive

BRAF AC245884.1 0.52962242910375 2.60594774925528e-31 postive

TSC1 AC090246.1 0.40921833153322 3.8079878572224e-18 postive

BRAF AC090246.1 0.508662835649849 1.25613078999157e-28 postive

TSC1 AC109460.1 0.424124544559553 1.66204766852616e-19 postive

BRAF Z98200.1 0.509742632017679 9.23230516815257e-29 postive

IPMK AC037487.2 0.404382627512504 1.01742610194473e-17 postive

OTULIN AC037487.2 0.463690148071733 1.84682235544465e-23 postive

BRAF AC037487.2 0.552217812614519 2.02195047157221e-34 postive

ATRX AC037487.2 0.674242093776443 3.36578319400723e-56 postive

ATRX AC011510.1 0.584262955533191 2.92882050939951e-39 postive

OTULIN AC105036.3 0.420037842109962 3.98368916060444e-19 postive

BRAF AC105036.3 0.569532160976182 5.71482255204997e-37 postive

ATRX AC105036.3 0.54697931122456 1.11675273626006e-33 postive

TSC1 AP001432.1 0.441164026748603 3.81004395064914e-21 postive

OTULIN AP001432.1 0.404044467423649 1.08915640328871e-17 postive

BRAF AP001432.1 0.60487084673676 1.14728933146227e-42 postive

ATRX AP001432.1 0.60832110224011 2.91562474304203e-43 postive

TSC1 AC129510.1 0.540769907313143 8.14605172412188e-33 postive

BRAF AC129510.1 0.544027940956772 2.88657963960066e-33 postive

ATRX AC129510.1 0.57526665840057 7.57133903592608e-38 postive

STAT3 AC002398.1 -0.423845825151856 1.76481971788489e-19 negative

TERT AC239803.3 0.445234732188644 1.49730790375173e-21 postive

BRAF AL117350.1 0.485710930810492 6.78716334858136e-26 postive

ATRX AL117350.1 0.408478442337253 4.43047375382712e-18 postive

TSC1 AC068533.3 0.423933332744036 1.73189699562905e-19 postive

MAPK8 AC068533.3 0.426782529015972 9.35266173935986e-20 postive

OTULIN AC068533.3 0.457974250251487 7.41469365924969e-23 postive

BRAF AC068533.3 0.713437568555014 1.27027778652732e-65 postive

ATRX AC068533.3 0.599307821080635 1.00871585130671e-41 postive

BRAF AC012186.2 0.420131473864327 3.90521691419363e-19 postive

TSC1 AC092828.1 0.410429112230457 2.96988177704319e-18 postive

MAPK8 AC092828.1 0.472203127740073 2.21836965288184e-24 postive

OTULIN AC092828.1 0.509717470727961 9.29890091937782e-29 postive

BRAF AC092828.1 0.824544081412824 5.9419969408643e-104 postive

ATRX AC092828.1 0.564986036620989 2.75927982623839e-36 postive

TRAF2 ARRDC1-AS1 0.501507700830043 9.4016044799869e-28 postive

IDH2 DCST1-AS1 0.425119448124541 1.34101773257579e-19 postive

TSC1 AC004837.2 0.419128958566568 4.83077075371993e-19 postive

MAPK8 AC004837.2 0.460035691763691 4.50493830779175e-23 postive

IPMK AC004837.2 0.443565648259808 2.1993019635726e-21 postive

OTULIN AC004837.2 0.50030275352753 1.3133949879866e-27 postive

BRAF AC004837.2 0.711832222051254 3.31739765629414e-65 postive

ATRX AC004837.2 0.64410007705342 6.8956138494377e-50 postive

KLF9 LINC00702 0.488705655025162 3.06720959336433e-26 postive

FASLG LINC00426 0.574172307102084 1.11693800763622e-37 postive

TNFRSF1B LINC00426 0.607409607847968 4.19384897407376e-43 postive

CYLD LINC00426 0.458562284869376 6.43446891317716e-23 postive

FLT3 LINC00426 0.668382121771604 6.48943590500858e-55 postive

BACH2 LINC00426 0.625380448457476 2.58721450893971e-46 postive

CD40 LINC00426 0.401243432932653 1.90929857651084e-17 postive

TSC1 AC007292.2 0.400295795766649 2.30584079902845e-17 postive

BRAF AC007292.2 0.540015299929406 1.03420040133355e-32 postive

ATRX AC007292.2 0.419204460804735 4.75412343870264e-19 postive

TSC1 LINC01675 0.412990802210234 1.74936675637516e-18 postive

OTULIN LINC01675 0.428112063600471 7.00162454242277e-20 postive

BRAF LINC01675 0.800765308345058 9.62688183526642e-94 postive

ATRX LINC01675 0.442054618575836 3.10924125984525e-21 postive

BRAF AC108681.1 0.486223808427202 5.92725797083363e-26 postive

TSC1 CTBP1-AS 0.431578566263656 3.27144732299663e-20 postive

TSC1 AP000873.2 0.518528940945012 7.23201260244961e-30 postive

BRAF AP000873.2 0.574848971305668 8.78407176328011e-38 postive

ATRX AP000873.2 0.446746843588249 1.05497854787081e-21 postive

BRAF AL031667.3 0.560828442148144 1.1401248666106e-35 postive

ATRX AL031667.3 0.408259698611355 4.6329628567147e-18 postive

MAP3K7 AL133338.1 0.604298247743232 1.43782700287579e-42 postive

TSC1 AC245884.10 0.474863571709853 1.1299791450307e-24 postive

MAPK8 AC245884.10 0.469352966485531 4.54001654974824e-24 postive

OTULIN AC245884.10 0.440763694341131 4.1737489272627e-21 postive

BRAF AC245884.10 0.76945417039071 3.09188073550238e-82 postive

ATRX AC245884.10 0.572156305164982 2.27740621114535e-37 postive

BRAF PAXIP1-AS2 0.436309326000631 1.14177223805789e-20 postive

SIRT1 PAXIP1-AS2 0.408628504269907 4.2966229721896e-18 postive

ATRX PAXIP1-AS2 0.544367693838455 2.58889285506883e-33 postive

TSC1 AL021368.2 0.52323188559505 1.79423084170045e-30 postive

MAPK8 AL021368.2 0.445093464015784 1.54696031590912e-21 postive

BRAF AL021368.2 0.67304366899523 6.19890188434514e-56 postive

SIRT1 AL021368.2 0.410285758080459 3.05874954595567e-18 postive

ATRX AL021368.2 0.607467170570995 4.09880618964042e-43 postive

TARDBP ATP2A1-AS1 0.440826144323746 4.11483978118646e-21 postive

TSC1 AL121652.1 0.414896685929304 1.17647356458785e-18 postive

MAPK8 AL121652.1 0.435989639093895 1.22658922185623e-20 postive

OTULIN AL121652.1 0.48165987463446 1.96304910541143e-25 postive

BRAF AL121652.1 0.803345319730678 8.79198572905206e-95 postive

ATRX AL121652.1 0.536373661374761 3.24474960950259e-32 postive

TSC1 AC073283.1 0.444887420376028 1.6223024709417e-21 postive

OTULIN AC073283.1 0.40385329490114 1.13188421012273e-17 postive

BRAF AC073283.1 0.596455132173857 3.0253187532034e-41 postive

ATRX AC073283.1 0.439009579677418 6.21450021770921e-21 postive

TSC1 AC005104.1 0.448751564508982 6.61396311499976e-22 postive

MAPK8 AC005104.1 0.439247119566747 5.8891655934383e-21 postive

BRAF AC005104.1 0.645626869348688 3.43538004460043e-50 postive

ATRX AC005104.1 0.6455144556752 3.61670675597404e-50 postive

ZBP1 AL139352.1 0.618613112294502 4.42535320890823e-45 postive

TSC1 MUC20-OT1 0.460054514194259 4.48441859657949e-23 postive

BRAF MUC20-OT1 0.536545686044029 3.07510210203131e-32 postive

ATRX MUC20-OT1 0.405817692237127 7.61317486784652e-18 postive

MAPK8 AC026782.2 0.413171863544718 1.6848433967164e-18 postive

BRAF AC026782.2 0.710946433936148 5.61833937886567e-65 postive

ATRX AC026782.2 0.480573965540867 2.60332909382042e-25 postive

TSC1 AC024361.3 0.512708382936463 3.94057443477851e-29 postive

MAPK8 AC024361.3 0.457345940168244 8.62501324415478e-23 postive

OTULIN AC024361.3 0.416906066165719 7.72204467659438e-19 postive

BRAF AC024361.3 0.757949393785259 1.86122173115911e-78 postive

ATRX AC024361.3 0.462611574481346 2.40550851726354e-23 postive

IPMK AL161891.1 0.404976470451462 9.02532406705142e-18 postive

DNMT1 AL161891.1 0.440027276397462 4.93432285691306e-21 postive

ATRX AL161891.1 0.502317909292129 7.50324110948286e-28 postive

TSC1 AL590729.1 0.521735581791914 2.8022923036604e-30 postive

MAPK8 AL590729.1 0.481474732111492 2.05998718328191e-25 postive

IPMK AL590729.1 0.422629915705073 2.29134441090957e-19 postive

OTULIN AL590729.1 0.488983424581154 2.84823761108425e-26 postive

BRAF AL590729.1 0.756830330040112 4.22796130741019e-78 postive

ATRX AL590729.1 0.677171832534917 7.47117897717352e-57 postive

OTULIN DENND6A-AS1 0.450736956801393 4.15248468144291e-22 postive

BRAF DENND6A-AS1 0.61578121226137 1.42264900662864e-44 postive

ATRX DENND6A-AS1 0.601162576367118 4.90971407929754e-42 postive

TSC1 AL031282.2 0.561116061072516 1.03420537101918e-35 postive

MAPK8 AL031282.2 0.419513233588979 4.45293202611586e-19 postive

BRAF AL031282.2 0.441806647697174 3.290481511285e-21 postive

ATRX AL031282.2 0.452381207823337 2.81763063809467e-22 postive

MAP3K7 DNM3OS 0.438840291274801 6.45710234523222e-21 postive

BRAF LINC00471 0.400343664848402 2.28399790025907e-17 postive

KLF9 MIR1-1HG-AS1 0.428446202408102 6.50895392826099e-20 postive

TSC1 AC006017.1 0.444031327097971 1.97602010531327e-21 postive

MAPK8 AC006017.1 0.431944632319924 3.01731379063735e-20 postive

OTULIN AC006017.1 0.43691708565702 9.96154270846808e-21 postive

BRAF AC006017.1 0.804797542458771 2.25014012944679e-95 postive

ATRX AC006017.1 0.519043685103018 6.21524815331489e-30 postive

BRAF AC093799.1 0.46683504417153 8.49982139391776e-24 postive

TSC1 AC026124.2 0.412814319042307 1.81459612705935e-18 postive

MAPK8 AC026124.2 0.479177722778551 3.73698426017993e-25 postive

OTULIN AC026124.2 0.503469703247584 5.4393544893464e-28 postive

BRAF AC026124.2 0.735464110188166 1.19737356035985e-71 postive

ATRX AC026124.2 0.569322267289297 6.14904144656666e-37 postive

MAPK8 CARMN 0.43359914193832 2.09101710892347e-20 postive

OTULIN CARMN 0.493320147719673 8.88234616896543e-27 postive

BRAF CARMN 0.737631639254104 2.83507389356782e-72 postive

ATRX CARMN 0.636362878674224 2.21742019909144e-48 postive

TSC1 LINC02569 0.445905175019783 1.28227411577227e-21 postive

BRAF LINC02569 0.640233340683163 3.95604622458626e-49 postive

ATRX LINC02569 0.425108748574755 1.34412162141387e-19 postive

BRAF AC004584.1 0.436134964001294 1.18728968739346e-20 postive

ATRX AC004584.1 0.472653778195044 1.97965369995356e-24 postive

TSC1 AL356481.1 0.496759225652149 3.48382545827212e-27 postive

BRAF AL356481.1 0.531955002073749 1.27543807693929e-31 postive

ATRX RMRP 0.448063264901744 7.76669288504476e-22 postive

OTULIN RC3H1-IT1 0.425439002923983 1.25150036229415e-19 postive

BRAF RC3H1-IT1 0.582849196644584 4.91560166517002e-39 postive

ATRX RC3H1-IT1 0.562757948544409 5.91672405032516e-36 postive

TSC1 AC089999.2 0.446040643360713 1.24267665494192e-21 postive

BRAF AC089999.2 0.623413021026101 5.94905616299417e-46 postive

ATRX AC089999.2 0.481151142523896 2.24089216366887e-25 postive

TSC1 AL355574.1 0.4696931609946 4.16952727866323e-24 postive

BRAF AC011389.2 0.555783711816092 6.20880275483593e-35 postive

TSC1 MANEA-DT 0.429638426393219 5.01372907543054e-20 postive

MAP3K7 MANEA-DT 0.488832896132795 2.96491253024027e-26 postive

BRAF MANEA-DT 0.532447641015525 1.09600807633741e-31 postive

ATRX MANEA-DT 0.570955731898728 3.47287744577064e-37 postive

TSC1 AC021087.2 0.462864335338086 2.26122950827544e-23 postive

OTULIN AC021087.2 0.533121595443297 8.90345070701246e-32 postive

BRAF AC021087.2 0.646228595452679 2.60760310237645e-50 postive

ATRX AC021087.2 0.451841498313055 3.20087232368605e-22 postive

TSC1 LINC00641 0.481892907530872 1.84741686070203e-25 postive

MAPK8 LINC00641 0.460628641663087 3.90094701873456e-23 postive

BRAF LINC00641 0.552404595870176 1.9013613791455e-34 postive

SIRT1 LINC00641 0.495820596825068 4.50248121482043e-27 postive

ATRX LINC00641 0.622507382969934 8.71044759576056e-46 postive

KLF9 MIR497HG 0.491498058077488 1.45219851556201e-26 postive

MAPK8 AC018752.1 0.422212357669973 2.5056705518081e-19 postive

IPMK AC018752.1 0.491437055050752 1.4762221555309e-26 postive

OTULIN AC018752.1 0.433653708814705 2.06580857270152e-20 postive

BRAF AC018752.1 0.60115188648783 4.93019820546448e-42 postive

SIRT1 AC018752.1 0.439889523009079 5.09104927617619e-21 postive

ATRX AC018752.1 0.759443832361076 6.17871172515039e-79 postive

TSC1 AC023024.1 0.458609495244605 6.36155870995555e-23 postive

BRAF AC023024.1 0.652127845505818 1.69017747264935e-51 postive

ATRX AC023024.1 0.432332238989474 2.76941421696498e-20 postive

OTULIN AC099850.3 0.403539494981302 1.20561916601242e-17 postive

DNMT1 AC099850.3 0.573088724941398 1.63908124041055e-37 postive

HAT1 AC099850.3 0.444824288375521 1.64610302376935e-21 postive

PLK1 AC099850.3 0.613797747293764 3.20073054199513e-44 postive

TSC1 AC131934.1 0.428892567407538 5.90372044220582e-20 postive

MAPK8 AC131934.1 0.426034206319504 1.10017341184111e-19 postive

OTULIN AC131934.1 0.465104529204027 1.30403079232252e-23 postive

BRAF AC131934.1 0.63275976720841 1.07938428449588e-47 postive

ATRX AC131934.1 0.648748233672138 8.16449261397497e-51 postive

TSC1 AC078778.2 0.472904142927824 1.85818050819524e-24 postive

MAPK8 AC078778.2 0.437052135377807 9.66370937388451e-21 postive

OTULIN AC078778.2 0.509800019909373 9.08217218901758e-29 postive

BRAF AC078778.2 0.722096452025435 6.37818634169457e-68 postive

ATRX AC078778.2 0.668529333868237 6.02950929575629e-55 postive

TSC1 AL391095.3 0.441288792932074 3.70321398290861e-21 postive

BRAF AL391095.3 0.48692892489227 4.91818160439126e-26 postive

ATRX AL391095.3 0.433336568672177 2.21662012501988e-20 postive

TSC1 AC124312.3 0.426133097395769 1.07684003435952e-19 postive

MAPK8 AC124312.3 0.433413778235476 2.17893463685999e-20 postive

BRAF AC124312.3 0.728495101400462 1.11871129941642e-69 postive

ATRX AC124312.3 0.449537200932546 5.50333784586557e-22 postive

MAPK8 AC025031.3 0.410598674474976 2.86804177831049e-18 postive

OTULIN AC025031.3 0.423930450326059 1.73297174538047e-19 postive

BRAF AC025031.3 0.676414893345928 1.10409634811365e-56 postive

ATRX AC025031.3 0.497246559288335 3.04848563347289e-27 postive

TERT AC092809.4 0.446513756401806 1.11361199635137e-21 postive

TSC1 AC087294.1 0.484395081843251 9.59802659984566e-26 postive

MAPK8 AC087294.1 0.474929176106802 1.11125505964402e-24 postive

OTULIN AC087294.1 0.498314533671965 2.27360024891853e-27 postive

BRAF AC087294.1 0.788912721016542 3.70778794748703e-89 postive

ATRX AC087294.1 0.623816293623473 5.01806423760643e-46 postive

MAPK8 AC005856.1 0.424722574388021 1.46101017318984e-19 postive

OTULIN AC005856.1 0.443914300647666 2.02993459988586e-21 postive

BRAF AC005856.1 0.711090830697713 5.15664786395488e-65 postive

ATRX AC005856.1 0.549201675476684 5.42864949264708e-34 postive

BRAF AC063943.1 0.595742720859004 3.97329941543928e-41 postive

DDX58 NRIR 0.443990401073483 1.99471242951583e-21 postive

BRAF AC093157.2 0.474138256063043 1.35892429654057e-24 postive

ATRX AC093157.2 0.473057143552875 1.7876001719338e-24 postive

TSC1 AC003991.2 0.453729389161679 2.04693464594733e-22 postive

MAPK8 AC003991.2 0.453955336862673 1.93992429581984e-22 postive

OTULIN AC003991.2 0.456364436420108 1.09161121473132e-22 postive

BRAF AC003991.2 0.849945931213785 1.01446624611248e-116 postive

ATRX AC003991.2 0.554679033392602 8.96431166964073e-35 postive

OTULIN AC093423.2 0.404182464169783 1.05930317560757e-17 postive

BRAF AC093423.2 0.405123360489531 8.76141713921149e-18 postive

ATRX AC093423.2 0.625232121418504 2.75540373695612e-46 postive

ATRX AC087481.3 0.400233083960526 2.33476746964816e-17 postive

TSC1 AC040934.1 0.461319640270611 3.29730498616538e-23 postive

MAPK8 AC040934.1 0.45808848819786 7.21338735172808e-23 postive

OTULIN AC040934.1 0.405267355703744 8.51008144706796e-18 postive

BRAF AC040934.1 0.67009192867182 2.75599984180487e-55 postive

ATRX AC040934.1 0.608051501476613 3.24700374787505e-43 postive

TSC1 AL031716.1 0.400482519461585 2.22178208623812e-17 postive

BRAF AL031716.1 0.563623020122403 4.40306653364777e-36 postive

TSC1 AL159169.3 0.40609081309623 7.20324998649687e-18 postive

BRAF AL159169.3 0.423937237173506 1.73044222104526e-19 postive

TSC1 C9orf163 0.425187292296452 1.32150005652977e-19 postive

LEF1 AC104971.1 0.409055019144155 3.93752512321349e-18 postive

TSC1 AC013403.2 0.527032660005758 5.72369599213489e-31 postive

TSC1 AL158834.2 0.466023453732921 1.03924070664451e-23 postive

BRAF AL158834.2 0.644434720046736 5.92102806206279e-50 postive

ATRX AL158834.2 0.478129527269625 4.89673435103079e-25 postive

OTULIN ADAMTSL4-AS1 0.41813440389566 5.9614106449087e-19 postive

BRAF ADAMTSL4-AS1 0.530099935906025 2.25236201393514e-31 postive

ATRX ADAMTSL4-AS1 0.602892596636914 2.49761601785924e-42 postive

TSC1 AC008543.1 0.463552273059183 1.91038326736675e-23 postive

MAPK8 AC008543.1 0.519419416034494 5.5636163180343e-30 postive

OTULIN AC008543.1 0.437419707871512 8.89679851939356e-21 postive

BRAF AC008543.1 0.659299846053576 5.58489334800074e-53 postive

SIRT1 AC008543.1 0.453450575348456 2.18703304358581e-22 postive

ATRX AC008543.1 0.535353452483647 4.45879468336336e-32 postive

TRIM11 STAG3L5P-PVRIG2P-PILRB 0.409333043811641 3.71951509175543e-18 postive

TSC1 AL359636.2 0.483993976085072 1.06641691064009e-25 postive

MAPK8 AL359636.2 0.424737364335409 1.45635457631887e-19 postive

BRAF AL359636.2 0.676344669483906 1.14476741043266e-56 postive

ATRX AL359636.2 0.480949820045405 2.36126679729568e-25 postive

MAPK8 TRAF3IP2-AS1 0.47236468314946 2.12969163827388e-24 postive

OTULIN TRAF3IP2-AS1 0.439283933947528 5.84026988247592e-21 postive

MAP3K7 TRAF3IP2-AS1 0.555854615932259 6.06386289685845e-35 postive

BRAF TRAF3IP2-AS1 0.566385244005626 1.70398404285915e-36 postive

SIRT1 TRAF3IP2-AS1 0.449671690606537 5.33259624274854e-22 postive

ATRX TRAF3IP2-AS1 0.604759551032552 1.19879039630345e-42 postive

TSC1 AL512506.1 0.451504293726408 3.46594635254873e-22 postive

MAPK8 AL512506.1 0.43525165362902 1.44681144096111e-20 postive

OTULIN AL512506.1 0.455779774155449 1.25560744278903e-22 postive

BRAF AL512506.1 0.787719605727481 1.03354760563912e-88 postive

ATRX AL512506.1 0.597244443723054 2.23494511856652e-41 postive

TNFRSF1B AL133415.1 0.421888058106912 2.68563557947272e-19 postive

LEF1 AL133415.1 0.419094125355245 4.86654099907318e-19 postive

TSC1 AC007365.1 0.416605827246454 8.22491995317181e-19 postive

MAPK8 AC007365.1 0.470042687894051 3.81996065532545e-24 postive

IPMK AC007365.1 0.444801453844566 1.65479604784753e-21 postive

OTULIN AC007365.1 0.545930781623923 1.566561966969e-33 postive

BRAF AC007365.1 0.741238149395783 2.49832576199543e-73 postive

ATRX AC007365.1 0.632934778898712 1.00000239318792e-47 postive

CYLD AL357060.1 0.475454620969491 9.71933106123632e-25 postive

MAPK8 AC004477.3 0.461136242531318 3.44788781564039e-23 postive

BRAF AC004477.3 0.532669067121269 1.0237240974824e-31 postive

TSC1 AC022165.1 0.411216447140968 2.52523871534976e-18 postive

MAPK8 AC022165.1 0.459364589886702 5.30030207653025e-23 postive

OTULIN AC022165.1 0.444329387509205 1.84499561816111e-21 postive

BRAF AC022165.1 0.715694170280499 3.25828513810048e-66 postive

ATRX AC022165.1 0.56275268229774 5.92736079385017e-36 postive

TSC1 AC005253.1 0.540659911718535 8.43476807721188e-33 postive

BRAF AC005253.1 0.535273989145972 4.57034829039085e-32 postive

ATRX AC005253.1 0.576092231340773 5.64115293002769e-38 postive

ATRX AC110609.1 0.406050438366625 7.26245162188973e-18 postive

GATA3 AC073335.2 0.445013384639655 1.57582341848367e-21 postive

MAP3K7 LINC02588 0.404589356079519 9.75884692304691e-18 postive

TSC1 AC007390.2 0.478537554872158 4.40819156497351e-25 postive

MAPK8 AC007390.2 0.465635453447383 1.14385964158217e-23 postive

OTULIN AC007390.2 0.439107378768609 6.07846396390249e-21 postive

BRAF AC007390.2 0.692013650211023 2.76475897347039e-60 postive

ATRX AC007390.2 0.627490296895609 1.05233957277295e-46 postive

MAPK8 DLEU1-AS1 0.440978567843338 3.97447706405004e-21 postive

OTULIN DLEU1-AS1 0.479443174867356 3.48922971430631e-25 postive

BRAF DLEU1-AS1 0.813798331928178 3.70788513069211e-99 postive

ATRX DLEU1-AS1 0.502305186745037 7.52989748851542e-28 postive

TSC1 AL121574.1 0.440159007245899 4.78889961716027e-21 postive

BRAF AL121574.1 0.55843213070637 2.55950687794239e-35 postive

OTULIN AC008635.1 0.415150725879339 1.11566257035419e-18 postive

BRAF AC008635.1 0.549719877111279 4.58467538450149e-34 postive

ATRX AC008635.1 0.603874471448869 1.69876336138788e-42 postive

TRIM11 AC040162.3 0.40385861919766 1.13067219584163e-17 postive

TRIM11 AC010168.2 0.487117019254887 4.67899263212313e-26 postive

MAPK8 AC068790.6 0.450844998947845 4.04826639721248e-22 postive

OTULIN AC068790.6 0.489216334038164 2.67658693787638e-26 postive

BRAF AC068790.6 0.706545526261002 7.47680480056706e-64 postive

ATRX AC068790.6 0.626759177101908 1.43838697852804e-46 postive

TSC1 AC055855.1 0.429619734691788 5.03432720117014e-20 postive

TSC1 AL365277.1 0.469025423541461 4.9273574688202e-24 postive

MAPK8 AL365277.1 0.461314825641877 3.30117393990835e-23 postive

IPMK AL365277.1 0.414256301448888 1.34461510069596e-18 postive

OTULIN AL365277.1 0.447873806860667 8.11734632536843e-22 postive

BRAF AL365277.1 0.738732977340475 1.35604445438479e-72 postive

ATRX AL365277.1 0.642848582426809 1.2170962269794e-49 postive

TSC1 AC006566.1 0.413955276739734 1.43161563976093e-18 postive

MAPK8 AC006566.1 0.440563598213747 4.36815363365355e-21 postive

OTULIN AC006566.1 0.474086819049412 1.37679791727337e-24 postive

BRAF AC006566.1 0.797230922019881 2.41434721881331e-92 postive

ATRX AC006566.1 0.509395766578744 1.01933950885046e-28 postive

TSC1 AC006001.2 0.481504809530759 2.0439235471785e-25 postive

BRAF AC006001.2 0.731910729466828 1.23220234938442e-70 postive

ATRX AC006001.2 0.411737406491619 2.26774302512749e-18 postive

TSC1 LAMC1-AS1 0.420697784402894 3.46201348638624e-19 postive

BRAF LAMC1-AS1 0.534219380301447 6.34028783294532e-32 postive

ATRX LAMC1-AS1 0.621850419799778 1.14768936564384e-45 postive

TSC1 ZNF252P-AS1 0.519377374544023 5.63303339543232e-30 postive

BRAF ZNF252P-AS1 0.639372217763373 5.81756073396563e-49 postive

BRAF AL158196.1 0.40829305452347 4.60150760189402e-18 postive

BRAF AP001469.1 0.522594975891773 2.16978989655415e-30 postive

ATRX AP001469.1 0.533177363149804 8.75146794612226e-32 postive

TSC1 AC103746.1 0.409459846087241 3.62406954644751e-18 postive

MAPK8 AC103746.1 0.403948223600285 1.11046520231999e-17 postive

OTULIN AC103746.1 0.416389660065268 8.60680081803944e-19 postive

BRAF AC103746.1 0.753599233070188 4.407657031446e-77 postive

ATRX AC103746.1 0.424588239535668 1.5039728194672e-19 postive

TSC1 LINC00663 0.418844768262682 5.13033072803095e-19 postive

GATA3 AC108860.2 0.430451919147438 4.19334013206349e-20 postive

TSC1 AC011815.2 0.465939439734249 1.061061591949e-23 postive

BRAF AC011815.2 0.501433345412983 9.59794685016762e-28 postive

BRAF AL591806.1 0.562195346696152 7.1669825111394e-36 postive

ATRX AL591806.1 0.422420193809296 2.39663346557912e-19 postive

BRAF AC010655.2 0.458615122813867 6.3529222135136e-23 postive

TSC1 AC011921.1 0.440416910613575 4.51631114954198e-21 postive

BRAF AC011921.1 0.422303303681579 2.457369385231e-19 postive

STAT3 AP003352.1 -0.425691648003049 1.18491472462423e-19 negative

TSC1 AC092802.2 0.5566132088427 4.70805178695215e-35 postive

MAPK8 AC092802.2 0.458892026220301 5.94195153929029e-23 postive

OTULIN AC092802.2 0.458793395781167 6.08523706706474e-23 postive

BRAF AC092802.2 0.817180804323644 1.24135482091973e-100 postive

ATRX AC092802.2 0.665717518010504 2.43732699392649e-54 postive

GATA3 AC127070.1 0.412617606578433 1.89012460018966e-18 postive

FASLG LINC02084 0.636325025981205 2.25484296960149e-48 postive

TNFRSF1B LINC02084 0.529369317026041 2.81509414636393e-31 postive

TSC1 AC019118.1 0.404198433297868 1.05590090360846e-17 postive

BRAF AC019118.1 0.431012725975686 3.706306191149e-20 postive

BRAF AC015908.2 0.449388300769305 5.69866550969748e-22 postive

BRAF AL008723.1 0.456154159750213 1.14800405786271e-22 postive

OTULIN AL024508.1 0.443155908141381 2.41620774096876e-21 postive

BRAF AL024508.1 0.706668815096745 6.95840136671595e-64 postive

ATRX AL024508.1 0.515127376477113 1.95559346611069e-29 postive

BCL2 AL109955.1 0.444639185607027 1.71788789029034e-21 postive

FLT3 AL109955.1 0.492800894461652 1.0221162178669e-26 postive

BACH2 AL109955.1 0.43993284260381 5.041241693627e-21 postive

TSC1 AP000866.1 0.614087002232482 2.84478510347296e-44 postive

BRAF AP000866.1 0.573956349036112 1.20580246654305e-37 postive

ATRX AP000866.1 0.570802931556613 3.66402239320226e-37 postive

LEF1 AC015660.2 0.43548346389603 1.3737413374238e-20 postive

ATRX AP003171.1 0.4811900485998 2.21833807212636e-25 postive

OTULIN RNF216-IT1 0.466961977081437 8.23633443558955e-24 postive

BRAF RNF216-IT1 0.569529937962737 5.71925837887717e-37 postive

ATRX RNF216-IT1 0.648251116861163 1.02755948089655e-50 postive

TSC1 AC008735.4 0.531435555153035 1.49613625206154e-31 postive

TSC1 AC084018.2 0.509514664899377 9.8533046553011e-29 postive

DIABLO AC084018.2 0.442194941344856 3.01107714323214e-21 postive

BRAF AC084018.2 0.507332360787429 1.8329463140322e-28 postive

ATRX AC084018.2 0.545587034879686 1.74994318177635e-33 postive

STAT3 AC016888.1 0.401174848363904 1.93559339404844e-17 postive

CD40 LINC00173 0.409171097170181 3.84501645190238e-18 postive

TSC1 TH2LCRR 0.446186483748555 1.20139431201371e-21 postive

MAPK8 TH2LCRR 0.552247452019001 2.00232173348074e-34 postive

OTULIN TH2LCRR 0.423355595019039 1.96097572002952e-19 postive

BRAF TH2LCRR 0.69186575971523 2.99832337665514e-60 postive

ATRX TH2LCRR 0.647821298274265 1.25317914153622e-50 postive

TRIM11 FLJ12825 0.424149420371487 1.65316694622322e-19 postive

TSC1 AL136221.1 0.426095525990609 1.08564705279805e-19 postive

MAPK8 AL136221.1 0.405068374811924 8.85930526049884e-18 postive

BRAF AL136221.1 0.660073598347475 3.84422389974001e-53 postive

ATRX AL136221.1 0.431449773096156 3.36578115913226e-20 postive

BRAF AC104041.1 0.659535798554342 4.98427254432548e-53 postive

ATRX AC104041.1 0.40042226767041 2.24857145142089e-17 postive

FASLG Z84484.1 0.406881881334567 6.1345380724938e-18 postive

MLKL Z84484.1 0.427463956873159 8.06419234047826e-20 postive

MAP3K7 AC008537.2 0.643794272038346 7.92453359593762e-50 postive

TSC1 AL138756.1 0.600371474155343 6.6786120005624e-42 postive

BRAF AL138756.1 0.676235982572559 1.21066442853688e-56 postive

ATRX AL138756.1 0.457948146990744 7.46146402457884e-23 postive

MAPK8 AC073316.3 0.449464503109271 5.5978640958559e-22 postive

OTULIN AC073316.3 0.466304287725888 9.69464655325377e-24 postive

BRAF AC073316.3 0.797013921017979 2.93634500214977e-92 postive

ATRX AC073316.3 0.528185611400064 4.03563822614199e-31 postive

TSC1 AC004832.4 0.423584018306707 1.86704190949813e-19 postive

IPMK AC004832.4 0.402407318850752 1.51306179685928e-17 postive

OTULIN AC004832.4 0.504968223223332 3.57268720599951e-28 postive

BRAF AC004832.4 0.625956706957261 2.02500120737482e-46 postive

ATRX AC004832.4 0.646025927221143 2.86153584774739e-50 postive

ID1 AL023284.4 0.447193463058236 9.51005988626192e-22 postive

HAT1 AL023284.4 -0.402567078433064 1.46541164665111e-17 negative

BRAF RAP2C-AS1 0.461066855542434 3.50661133871048e-23 postive

ATRX RAP2C-AS1 0.616592696186281 1.01929656874496e-44 postive

ATRX AL122035.2 0.503569142496831 5.29007016535892e-28 postive

BRAF AC011978.2 0.510976505166949 6.48513472854392e-29 postive

TSC1 AL021878.2 0.507335783222252 1.83116934677176e-28 postive

MAPK8 AL021878.2 0.460391390440316 4.1323750976699e-23 postive

BRAF AL021878.2 0.624514064213823 3.73588190005244e-46 postive

SIRT1 AL021878.2 0.406525799957636 6.59477080112628e-18 postive

ATRX AL021878.2 0.508304500220963 1.39093465669198e-28 postive

TSC1 FTX 0.406886990813243 6.12816953635911e-18 postive

MAPK8 FTX 0.454836289711443 1.57289214163115e-22 postive

IPMK FTX 0.422225387829353 2.49869334167175e-19 postive

OTULIN FTX 0.493914343618829 7.56183280823773e-27 postive

BRAF FTX 0.698299259247031 8.40918382334126e-62 postive

ATRX FTX 0.669229796622118 4.24742452546833e-55 postive

ID1 RAB11B-AS1 0.444139247021875 1.92755325255592e-21 postive

HAT1 RAB11B-AS1 -0.422554768156183 2.32853736502259e-19 negative

TSC1 AC004832.5 0.409955314923069 3.27368184097277e-18 postive

OTULIN AC004832.5 0.561591054525551 8.80204898086499e-36 postive

BRAF AC004832.5 0.662025607723315 1.49090244201829e-53 postive

ATRX AC004832.5 0.607724263281182 3.69971833158183e-43 postive

TSC1 AC004263.1 0.455577309863067 1.31788133170622e-22 postive

BRAF AC004263.1 0.545265842619264 1.94041995369779e-33 postive

MAPK8 AC018410.2 0.403155940051197 1.30218144960771e-17 postive

OTULIN AC018410.2 0.443914521071614 2.02983169934452e-21 postive

BRAF AC018410.2 0.569280993429458 6.23819381424734e-37 postive

ATRX AC018410.2 0.573834083752327 1.25918109793483e-37 postive

FASLG AC015819.1 0.545328648484449 1.90162692614409e-33 postive

TNFRSF1B AC015819.1 0.583316108439579 4.14407416648599e-39 postive

FLT3 AC015819.1 0.606865798324789 5.20671341763898e-43 postive

BACH2 AC015819.1 0.432366590213698 2.74843778210684e-20 postive

STAT3 AL390294.1 -0.435157818531051 1.47746660246367e-20 negative

GATA3 AL390294.1 0.668296409834481 6.77308198466371e-55 postive

ATRX KCNQ1OT1 0.445944005393066 1.27079853426709e-21 postive

BRAF DTX2P1-UPK3BP1-PMS2P11 0.561850498647175 8.05914767860121e-36 postive

ATRX DTX2P1-UPK3BP1-PMS2P11 0.410679038484834 2.82098236048323e-18 postive

TSC1 AC007686.3 0.480352234205043 2.75745545774265e-25 postive

BRAF AC007686.3 0.5603938846578 1.32084917506747e-35 postive

ATRX AC007686.3 0.424835801692028 1.42573833592592e-19 postive

BRAF LNX1-AS2 0.58400394109763 3.2208911075065e-39 postive

KLF9 AF001548.1 0.426066059860558 1.09260372260576e-19 postive

TSC1 AC011773.1 0.430030032096271 4.60077463867264e-20 postive

BRAF AC011773.1 0.5404633041146 8.97626484740233e-33 postive

ATRX AC011773.1 0.434310876966348 1.7847468728631e-20 postive

BRAF AL590006.1 0.524821983485242 1.11441915505676e-30 postive

ATRX AL590006.1 0.505085864130445 3.45641216797313e-28 postive

MAPK8 AC120114.1 0.435407394348661 1.39730956869827e-20 postive

BRAF AC120114.1 0.464972838763703 1.34706851007512e-23 postive

SIRT1 AC120114.1 0.408555067786572 4.36162159202577e-18 postive

ATRX AC120114.1 0.403988709142959 1.10145203725803e-17 postive

BACH2 AC017104.1 0.64532729995104 3.93988430855971e-50 postive

CD40 AC017104.1 0.429325297056273 5.37000947407261e-20 postive

IPMK ENTPD1-AS1 0.454832984474108 1.57413212176238e-22 postive

OTULIN ENTPD1-AS1 0.435531902580266 1.35893837671145e-20 postive

BRAF ENTPD1-AS1 0.491461726888728 1.46645920950052e-26 postive

ATRX ENTPD1-AS1 0.699351932117351 4.6434735368288e-62 postive

ATRX AC040904.1 0.553442382337455 1.35013590948503e-34 postive

BACH2 AC090152.1 0.479948903661391 3.06125537265845e-25 postive

ATRX STARD13-AS 0.58203884891469 6.60666571479912e-39 postive

TRIM11 AC010542.5 0.467527167103305 7.15765364578018e-24 postive

MAPK8 AC022211.1 0.425112193167778 1.34312158999619e-19 postive

BRAF AC022211.1 0.537047715365225 2.6285847164092e-32 postive

ATRX AC022211.1 0.545894516901404 1.58497412333196e-33 postive

TSC1 AC012645.2 0.459577356122027 5.03420329026249e-23 postive

BRAF AC012645.2 0.655653700008997 3.19850918962307e-52 postive

ATRX AC012645.2 0.416477326202827 8.44987127347919e-19 postive

MAPK8 AL109614.1 0.450022929462665 4.91092455508164e-22 postive

OTULIN AL109614.1 0.423080464623664 2.0803091470365e-19 postive

BRAF AL109614.1 0.693538908086599 1.19442873422219e-60 postive

ATRX AL109614.1 0.555230450470593 7.46397161760748e-35 postive

TRIM11 AC244034.2 0.464881453574736 1.37775473844536e-23 postive

TSC1 AL450344.3 0.409106994982552 3.89583528879359e-18 postive

MAPK8 AL450344.3 0.445004646187309 1.57900497154475e-21 postive

OTULIN AL450344.3 0.479613220696088 3.33911699675088e-25 postive

BRAF AL450344.3 0.825960644752757 1.31035890234819e-104 postive

ATRX AL450344.3 0.508816939726552 1.20220737795798e-28 postive

TSC1 AC106820.5 0.441904653338907 3.21763819581367e-21 postive

MAPK8 AC106820.5 0.467686646112235 6.87936291100893e-24 postive

BRAF AC106820.5 0.530962554117156 1.72974149871657e-31 postive

SIRT1 AC106820.5 0.41069922275349 2.80928274428172e-18 postive

ATRX AC106820.5 0.512211354116068 4.54756577562516e-29 postive

KLF9 AC005180.2 0.488554200160064 3.19352540600096e-26 postive

BRAF AC022639.1 0.67395839138917 3.89033174235072e-56 postive

MLKL AC116407.2 0.422351560334803 2.43211382487913e-19 postive

TSC1 AL390067.1 0.528993670432135 3.1564508227458e-31 postive

BRAF AL390067.1 0.55408670305594 1.09094068884899e-34 postive

TSC1 AL132780.1 0.443623623216784 2.1702038338988e-21 postive

ATRX AL132780.1 0.441205818218337 3.77392637842753e-21 postive

RNF31 AL132780.1 0.443590775239813 2.1866436023779e-21 postive

BRAF AC006206.2 0.413602123923033 1.54076007825362e-18 postive

TSC1 AC007128.1 0.463395976744075 1.98504911141569e-23 postive

MAPK8 AC007128.1 0.415676576428314 9.99446524849965e-19 postive

BRAF AC007128.1 0.712758079574997 1.90857011868589e-65 postive

ATRX AC007128.1 0.493353183255249 8.80329014144156e-27 postive

OTULIN LINC01194 0.408539933893887 4.37513629562922e-18 postive

BRAF LINC01194 0.518794997390922 6.68748311993475e-30 postive

TSC1 PDXDC2P-NPIPB14P 0.48411076089108 1.03422312832682e-25 postive

BRAF PDXDC2P-NPIPB14P 0.566791415524889 1.48085528164115e-36 postive

GATA3 AC020663.2 0.454270280231333 1.79991373150179e-22 postive

STAT3 LINC002481 -0.408634485344281 4.29137127792738e-18 negative

GATA3 LINC002481 0.404883402183332 9.1965622227155e-18 postive

BRAF AL031432.3 0.413706249344027 1.50775524814976e-18 postive

FAS LINC02081 0.405228841998094 8.57660183763043e-18 postive

AXL LINC02081 0.441181797178127 3.79464471817366e-21 postive

TSC1 AC090589.3 0.604753750084428 1.2015366713686e-42 postive

BRAF AC090589.3 0.4901457807934 2.08761109342809e-26 postive

ATRX AC090589.3 0.498319000357723 2.27080820718615e-27 postive

TSC1 MCM3AP-AS1 0.491722607073631 1.36704941022588e-26 postive

MAPK8 MCM3AP-AS1 0.443729129201979 2.11821984377707e-21 postive

BRAF MCM3AP-AS1 0.617774195043788 6.26215353190558e-45 postive

ATRX MCM3AP-AS1 0.595247787540765 4.79976239649546e-41 postive

TSC1 MCCC1-AS1 0.469854070444148 4.00484375573781e-24 postive

BRAF MCCC1-AS1 0.581789828705582 7.23384638051712e-39 postive

ATRX MCCC1-AS1 0.416992415526038 7.58310546135645e-19 postive

AXL LINC02104 0.536853402457582 2.79324166877911e-32 postive

TSC1 LINC01376 0.451678589897897 3.32633847240081e-22 postive

BRAF LINC01376 0.62472155110208 3.42157676037305e-46 postive

ATRX LINC01376 0.446505812605562 1.11566587426793e-21 postive

TERT AC092811.1 0.5539319522334 1.1482969137672e-34 postive

TSC1 AL080317.1 0.424761082920941 1.44891888957044e-19 postive

BRAF AL080317.1 0.568229459449742 8.99559875015162e-37 postive

MAP3K7 AC004449.1 0.434498416994402 1.71169352466299e-20 postive

TSC1 AC007255.1 0.410761311356766 2.77359211389633e-18 postive

BRAF AC007255.1 0.568455567084 8.31563084892934e-37 postive

TSC1 AC093788.1 0.421627426981502 2.83943961694398e-19 postive

TSC1 AC097634.1 0.500204620199303 1.34956814092134e-27 postive

BRAF AC097634.1 0.599344873389396 9.94355705300366e-42 postive

ATRX AC097634.1 0.475542193990196 9.50452665048665e-25 postive

TSC1 MIR600HG 0.706784514429471 6.50442869660383e-64 postive

MAPK8 MIR600HG 0.422779873323048 2.2188649071542e-19 postive

BRAF MIR600HG 0.540331329941343 9.35889829105564e-33 postive

TSC1 MORF4L2-AS1 0.610515114648974 1.20948037377209e-43 postive

BRAF MORF4L2-AS1 0.60170689032711 3.97096579438495e-42 postive

ATRX MORF4L2-AS1 0.440336330085626 4.59979515907985e-21 postive

BNIP3 MEG9 0.460177254132805 4.35285342266999e-23 postive

TSC1 AC008434.1 0.492194176823454 1.20395798947235e-26 postive

BRAF AC008434.1 0.473506574431819 1.59522117739201e-24 postive

ATRX AC008434.1 0.591003980448656 2.39395619391906e-40 postive

BRAF MIR34AHG 0.466734338677749 8.71476754000276e-24 postive

OTULIN AC104984.6 0.459431752017881 5.21484020272953e-23 postive

BRAF AC104984.6 0.574837326898969 8.82050289537379e-38 postive

ATRX AC104984.6 0.653136359248531 1.05225537153734e-51 postive

MAP3K7 FOXD3-AS1 0.416101440233444 9.14322493989688e-19 postive

BRAF AC068196.1 0.521011798211584 3.47402643250496e-30 postive

MYCN AL161772.1 0.490642894151206 1.82720296787608e-26 postive

BRAF AC009955.2 0.479194260688015 3.7210540423497e-25 postive

TSC1 AC010300.1 0.468228570948188 6.01114350099385e-24 postive

BRAF AC010300.1 0.762714515381722 5.37606351232666e-80 postive

ATRX AC010300.1 0.433333330733682 2.21821449105571e-20 postive

LEF1 LINC02178 0.509840459588271 8.97782953726154e-29 postive

DIABLO UBL7-AS1 0.420238675077144 3.81723924923724e-19 postive

BRAF UBL7-AS1 0.511828318007807 5.07760373015663e-29 postive

ATRX UBL7-AS1 0.48585645182701 6.53138108430462e-26 postive

TSC1 AC015802.4 0.456471557952825 1.06394242463469e-22 postive

BRAF AC026202.2 0.521626178725748 2.89489950155639e-30 postive

ATRX AC026202.2 0.493238377578363 9.08105601703588e-27 postive

TNFRSF1B AL034397.3 0.423154244550628 2.04762430304167e-19 postive

FLT3 AL034397.3 0.47146346477094 2.67319062051394e-24 postive

GATA3 AC007292.1 0.429814415547238 4.82381525786456e-20 postive

TSC1 ODF2-AS1 0.481179531182085 2.22441288369368e-25 postive

MAPK8 ODF2-AS1 0.441761874027659 3.32429834739226e-21 postive

OTULIN ODF2-AS1 0.435916804936766 1.24676475207774e-20 postive

BRAF ODF2-AS1 0.656663842237852 1.97707323365213e-52 postive

ATRX ODF2-AS1 0.618674984890702 4.31330082108206e-45 postive

TSC1 AC005522.1 0.42580086600668 1.15722144762593e-19 postive

MAPK8 AC005522.1 0.44090788619965 4.038969213102e-21 postive

OTULIN AC005522.1 0.504104325691743 4.55351407781009e-28 postive

BRAF AC005522.1 0.755681568765698 9.77040899678083e-78 postive

ATRX AC005522.1 0.610788873756105 1.08319876115968e-43 postive

TSC1 AC010973.1 0.407095607237236 5.87362577624923e-18 postive

BRAF AC010973.1 0.425587637866693 1.2118936527414e-19 postive

ATRX AC010973.1 0.548429020319903 6.98025263158289e-34 postive

BRAF AC022893.1 0.482593112282932 1.53893148680448e-25 postive

OTULIN AL137779.1 0.461546563792908 3.11993909841843e-23 postive

BRAF AL137779.1 0.530711324267675 1.86813402891529e-31 postive

ATRX AL137779.1 0.633351319835311 8.33596888890434e-48 postive

TSC1 YEATS2-AS1 0.492498283762245 1.10913874120654e-26 postive

MAPK8 YEATS2-AS1 0.43569423885694 1.31046475049089e-20 postive

BRAF YEATS2-AS1 0.624340092289305 4.02135880838577e-46 postive

ATRX YEATS2-AS1 0.588001944227812 7.35533794120818e-40 postive

TSC1 AC008676.1 0.414143465715849 1.37659909742845e-18 postive

BRAF AC008676.1 0.60776589870015 3.63881536150061e-43 postive

TNFRSF1B TSPOAP1-AS1 0.436138565929658 1.18633155857623e-20 postive

FLT3 TSPOAP1-AS1 0.537827903057547 2.05875411358157e-32 postive

BACH2 TSPOAP1-AS1 0.598820761970311 1.21772567507116e-41 postive

LEF1 TSPOAP1-AS1 0.476098714400198 8.24489102380645e-25 postive

TSC1 AC009121.1 0.527698651671539 4.67823782598807e-31 postive

MAPK8 AC009121.1 0.433802472850967 1.9985939740392e-20 postive

OTULIN AC009121.1 0.424405701033877 1.56435112536082e-19 postive

BRAF AC009121.1 0.560864118737327 1.12642443059338e-35 postive

ATRX AC009121.1 0.473743577739245 1.50215490249738e-24 postive

BRAF GLYCTK-AS1 0.558374751996436 2.60934091980485e-35 postive

ATRX GLYCTK-AS1 0.552306648191499 1.96368174168623e-34 postive

TSC1 AC011447.3 0.414276400407167 1.33899515134901e-18 postive

MAPK8 AC011447.3 0.413924564982659 1.44079717178518e-18 postive

BRAF AC011447.3 0.634737387744916 4.53999379413638e-48 postive

ATRX AC011447.3 0.437910547891535 7.96549475952548e-21 postive

ATRX AC012358.3 0.417244142811633 7.19195516698586e-19 postive

KLF9 AP003071.4 0.588268225535214 6.66143717906518e-40 postive

BRAF AL032819.1 0.503974095725396 4.72282776136607e-28 postive

MAPK8 AC091729.2 0.438627448971011 6.77539765203926e-21 postive

OTULIN AC091729.2 0.500100239498464 1.38912481767817e-27 postive

BRAF AC091729.2 0.610380760535485 1.27668948064049e-43 postive

ATRX AC091729.2 0.531735683627123 1.36438991959724e-31 postive

TSC1 AL358072.1 0.503456222165754 5.45991095935488e-28 postive

BRAF AL358072.1 0.532061781562278 1.23423118398052e-31 postive

BNIP3 LINC01134 0.554952978175081 8.18495740354791e-35 postive

OTULIN ALMS1-IT1 0.407037185648133 5.94384164577517e-18 postive

TRIM11 LINC01820 0.407899767262292 4.98613099723401e-18 postive

FLT3 AC093278.2 0.462784257939771 2.30598946429133e-23 postive

KLF9 AC093278.2 0.481519156833583 2.0363046250119e-25 postive

ATRX RASA3-IT1 0.492156686247568 1.21618824837357e-26 postive

ID1 AC080112.1 0.404676702732089 9.5883809896957e-18 postive

TSC1 AC245884.9 0.498807147829724 1.98523604524704e-27 postive

MAPK8 AC245884.9 0.410222758373044 3.09862672529927e-18 postive

BRAF AC245884.9 0.705738569949859 1.19555702966186e-63 postive

ATRX AC245884.9 0.507147780438663 1.9313498284305e-28 postive

OTULIN EGOT 0.453335665434283 2.24748706826668e-22 postive

BRAF EGOT 0.481810136070502 1.8876970905361e-25 postive

ATRX EGOT 0.45294644472524 2.46475861685023e-22 postive

BRAF AGAP1-IT1 0.418318739266776 5.73381845155635e-19 postive

ATRX AGAP1-IT1 0.463041087089697 2.1654442213533e-23 postive

IPMK AC007319.1 0.408933777513623 4.03648623816994e-18 postive

OTULIN AC007319.1 0.404749042682951 9.44942156643089e-18 postive

CFLAR AC007319.1 0.409344724004528 3.71062087838565e-18 postive

BRAF AC007319.1 0.441057152735769 3.90396469605313e-21 postive

ATRX AC007319.1 0.479936709383681 3.07093747358419e-25 postive

IPMK AC058791.1 0.429003792981266 5.76174934441506e-20 postive

OTULIN AC058791.1 0.474661745570701 1.18955667796148e-24 postive

BRAF AC058791.1 0.578668641071475 2.23965122274601e-38 postive

ATRX AC058791.1 0.665119406508933 3.27416600347661e-54 postive

MAPK8 AL133243.3 0.420831649897342 3.36471009703628e-19 postive

IPMK AL133243.3 0.406021149732143 7.30569685801705e-18 postive

OTULIN AL133243.3 0.444924320878439 1.60854851571782e-21 postive

BRAF AL133243.3 0.625590359812506 2.36645222572746e-46 postive

ATRX AL133243.3 0.666763121452602 1.45246339207388e-54 postive

MAPK8 AC244093.4 0.421643583812321 2.82965805846126e-19 postive

OTULIN AC244093.4 0.479771621481182 3.20502897316107e-25 postive

BRAF AC244093.4 0.639272114132246 6.08380604376278e-49 postive

ATRX AC244093.4 0.613507552979065 3.60215014958228e-44 postive

TSC1 AC009704.2 0.415889659273831 9.55822219980707e-19 postive

OTULIN AC009704.2 0.414230406089073 1.35189002930818e-18 postive

BRAF AC009704.2 0.638669678915158 7.96126062699989e-49 postive

ATRX AC009704.2 0.568021290274271 9.67015752368626e-37 postive

MAPK8 AC008114.1 0.408112974286558 4.77385638210064e-18 postive

IPMK AC008114.1 0.412545082964846 1.91874361675959e-18 postive

OTULIN AC008114.1 0.42023312344339 3.82174706597492e-19 postive

BRAF AC008114.1 0.651841142904645 1.9332918596538e-51 postive

ATRX AC008114.1 0.585612597668533 1.78229009553212e-39 postive

TSC1 AC011466.1 0.479545877808357 3.39778758018295e-25 postive

MAPK8 AC011466.1 0.418567042019245 5.4407155697544e-19 postive

IPMK AC011466.1 0.404717060402839 9.51061111053126e-18 postive

OTULIN AC011466.1 0.507275291792571 1.86282958406195e-28 postive

BRAF AC011466.1 0.634348459241924 5.38568964586915e-48 postive

ATRX AC011466.1 0.616531934053852 1.04509861971359e-44 postive

GATA3 MCF2L-AS1 0.546871291677757 1.15644220069329e-33 postive

BRAF AC092755.2 0.469369585096626 4.52118596275277e-24 postive

ATRX AC092755.2 0.442607973167132 2.73952155854722e-21 postive

TSC1 AP001630.1 0.411100038888126 2.58659425564324e-18 postive

MAPK8 AP001630.1 0.43452580703398 1.70127367524103e-20 postive

OTULIN AP001630.1 0.432468658420702 2.68702925058481e-20 postive

BRAF AP001630.1 0.803129509723482 1.07552608888748e-94 postive

ATRX AP001630.1 0.462388131240485 2.54059130214282e-23 postive

TSC1 AC068790.5 0.445756683601435 1.32710783565216e-21 postive

MAPK8 AC068790.5 0.436677129667987 1.05132044983954e-20 postive

OTULIN AC068790.5 0.439214399764606 5.93296144590702e-21 postive

BRAF AC068790.5 0.662508945075141 1.17791596348887e-53 postive

ATRX AC068790.5 0.621500242731861 1.32909443731415e-45 postive

OTULIN AC124283.3 0.434190563750504 1.83322140110147e-20 postive

BRAF AC124283.3 0.55906810658217 2.066452471964e-35 postive

ATRX AC124283.3 0.588076406539657 7.15438843834358e-40 postive

TSC1 AC121764.1 0.447597286671416 8.6573182692323e-22 postive

BRAF AC121764.1 0.607326317022385 4.33525086454044e-43 postive

ATRX AC121764.1 0.527333324619435 5.22583623755532e-31 postive

ATRX AC069549.1 0.511406819459328 5.73160740079331e-29 postive

IPMK AC087286.4 0.411717957379287 2.27687449246375e-18 postive

OTULIN AC087286.4 0.462937604253731 2.22102671954103e-23 postive

BRAF AC087286.4 0.589826892267895 3.72276187002513e-40 postive

ATRX AC087286.4 0.651521432062024 2.2454713619107e-51 postive

MAPK8 AL121989.1 0.43220361229761 2.84936967882295e-20 postive

OTULIN AL121989.1 0.471620272421834 2.56965800883051e-24 postive

BRAF AL121989.1 0.683901580943177 2.20134652688152e-58 postive

ATRX AL121989.1 0.639135728321392 6.46609562928108e-49 postive

ATRX AL355102.1 0.506187216822443 2.53412367742181e-28 postive

TNFRSF1B AC073072.1 0.433529126943632 2.12380654872539e-20 postive

KLF9 AC073072.1 0.430119210608182 4.51152331347753e-20 postive

TSC1 AC010998.2 0.504670348689823 3.88467334989144e-28 postive

BRAF AC010998.2 0.446836675437299 1.03320296533678e-21 postive

ATRX AC010998.2 0.496055761816167 4.22255281643942e-27 postive

KLF9 AC005180.1 0.477695546838388 5.47505899594538e-25 postive

TSC1 AC008124.1 0.443182013509401 2.40178055001022e-21 postive

BRAF AC008124.1 0.461006977815279 3.55807928878809e-23 postive

TSC1 AC078852.2 0.407090431139206 5.87981375684199e-18 postive

MAPK8 AC078852.2 0.459140003668052 5.59623864688836e-23 postive

OTULIN AC078852.2 0.481218517822689 2.20197643965407e-25 postive

BRAF AC078852.2 0.739923728205537 6.08348144090457e-73 postive

ATRX AC078852.2 0.581961736329377 6.79489379606426e-39 postive

FASLG AC006369.1 0.613731605641791 3.28813084987904e-44 postive

TNFRSF1B AC006369.1 0.576275535119882 5.28374541481019e-38 postive

FLT3 AC006369.1 0.552189312044435 2.04100458793184e-34 postive

TSC1 AC004253.1 0.484415169905942 9.54749347497567e-26 postive

TSC1 AL450998.2 0.453355337781584 2.23702180680681e-22 postive

MAPK8 AL450998.2 0.405658064704452 7.86329126833445e-18 postive

IPMK AL450998.2 0.400050617083357 2.42097691207599e-17 postive

OTULIN AL450998.2 0.432483782635641 2.67804560663853e-20 postive

BRAF AL450998.2 0.555355975964942 7.15881367382167e-35 postive

ATRX AL450998.2 0.677673299903291 5.76418550274141e-57 postive

TRIM11 AP002761.1 0.504789450670366 3.7568238110857e-28 postive

OTULIN AL356124.1 0.420099027883221 3.9322358943803e-19 postive

BRAF AL356124.1 0.594542420743289 6.27966080665807e-41 postive

ATRX AL356124.1 0.66601598341514 2.1029823499828e-54 postive

ATRX AC007637.1 0.422241067712745 2.49032264540343e-19 postive

PANX1 MYOSLID 0.466766664721389 8.64519265296313e-24 postive

TSC1 AC004148.2 0.449347437585664 5.75345480099974e-22 postive

FASLG LINC01914 0.510037556425109 8.48599854879953e-29 postive

TNFRSF1B LINC01914 0.466858779171862 8.44992805188945e-24 postive

TSC1 AL161729.4 0.433469884855296 2.15194601235468e-20 postive

TSC1 AC100778.3 0.465737152099247 1.11547356586981e-23 postive

BRAF AC100778.3 0.402816140363794 1.3940537048453e-17 postive

OTULIN AL133445.2 0.418000124591921 6.1327748742005e-19 postive

BRAF AL133445.2 0.441041340639995 3.91805272873836e-21 postive

ATRX AL133445.2 0.618362432951265 4.90963711960393e-45 postive

BRAF AC073046.1 0.586540638147436 1.26491794612346e-39 postive

ATRX AC073046.1 0.465056199792934 1.31966538670132e-23 postive

TSC1 GARS-DT 0.564285968730496 3.50878418138706e-36 postive

BRAF GARS-DT 0.698235285718193 8.71749017715004e-62 postive

ATRX GARS-DT 0.549959890026705 4.23912917057281e-34 postive

TSC1 AL157838.1 0.40559961072156 7.95688814016203e-18 postive

MAPK8 AL157838.1 0.450717030542219 4.17199259091152e-22 postive

IPMK AL157838.1 0.404547175907056 9.84222937842816e-18 postive

OTULIN AL157838.1 0.443038334978604 2.48225056866308e-21 postive

BRAF AL157838.1 0.725956245848538 5.64179361949852e-69 postive

ATRX AL157838.1 0.616423958317807 1.09255689530281e-44 postive

STAT3 GATA3-AS1 -0.450490133689157 4.40059963212497e-22 negative

GATA3 GATA3-AS1 0.729933258441356 4.43734279726635e-70 postive

TSC1 WASHC5-AS1 0.487451646306903 4.28153140221432e-26 postive

MAPK8 WASHC5-AS1 0.492959591272655 9.79211685655771e-27 postive

IPMK WASHC5-AS1 0.425538710928051 1.22479298247529e-19 postive

OTULIN WASHC5-AS1 0.527687658696713 4.69385551954344e-31 postive

BRAF WASHC5-AS1 0.803514245307101 7.50746993002404e-95 postive

ATRX WASHC5-AS1 0.675285573052269 1.97300010610534e-56 postive

GATA3 AC124068.2 0.420424666616442 3.66920546969999e-19 postive

TSC1 AC008735.1 0.511968365747038 4.8770786360008e-29 postive

BRAF AC008735.1 0.450067813301475 4.85946690947481e-22 postive

ATRX AC008735.1 0.480431976263249 2.70101600286761e-25 postive

BRAF TMEM202-AS1 0.446898050334057 1.01858084324573e-21 postive

ATRX TMEM202-AS1 0.41382988735773 1.469467967704e-18 postive

TSC1 AP001469.2 0.434028499959023 1.90057291663345e-20 postive

MAPK8 AP001469.2 0.414259240342568 1.34379189864874e-18 postive

BRAF AP001469.2 0.590540737433292 2.84887161506494e-40 postive

ATRX AP001469.2 0.641577579723843 2.16146107091867e-49 postive

FLT3 AC136475.2 0.414079205965558 1.39514724555206e-18 postive

KLF9 AP001189.1 0.414031697952246 1.40901800191295e-18 postive

CDKN2A CDKN2A-DT 0.808999707528911 4.08383749672778e-97 postive

TSC1 AP001619.1 0.538690687102828 1.57010942227462e-32 postive

MAPK8 AP001619.1 0.454742609137134 1.60841305697754e-22 postive

OTULIN AP001619.1 0.427316302144843 8.32764451950383e-20 postive

BRAF AP001619.1 0.740189444828529 5.08402583011378e-73 postive

ATRX AP001619.1 0.621677285459851 1.23408343918527e-45 postive

MAPK8 AC004943.1 0.437245644867121 9.25218577179899e-21 postive

OTULIN AC004943.1 0.475152999986518 1.04964927602325e-24 postive

BRAF AC004943.1 0.615621820997158 1.5187603437284e-44 postive

ATRX AC004943.1 0.575909062996145 6.02219735696075e-38 postive

AXL HHIP-AS1 0.475497640257481 9.6132185558594e-25 postive

TSC1 AC025682.1 0.444086734642188 1.95098840621905e-21 postive

MAPK8 AC025682.1 0.427504023821839 7.99413018295791e-20 postive

BRAF AC025682.1 0.597312417891566 2.17733322563505e-41 postive

ATRX AC025682.1 0.458590545528309 6.39072562822131e-23 postive

MAPK8 AL358216.1 0.400598141095743 2.17124977341542e-17 postive

OTULIN AL358216.1 0.430708727814675 3.96295358695693e-20 postive

BRAF AL358216.1 0.672816915986754 6.9559740488761e-56 postive

ATRX AL358216.1 0.562106590305677 7.38679731507142e-36 postive

MAPK8 AC078960.1 0.467929773404915 6.47552553185729e-24 postive

OTULIN AC078960.1 0.470061323249171 3.80215805993996e-24 postive

BRAF AC078960.1 0.748220594452332 2.01540869139189e-75 postive

ATRX AC078960.1 0.556526498555369 4.84639111629843e-35 postive

TSC1 AL359265.3 0.406030419192596 7.29198300182563e-18 postive

BRAF AC012360.1 0.489082795762808 2.77371188411038e-26 postive

FASLG AC090559.1 0.462181307564167 2.67228207545904e-23 postive

TNFRSF1B AC090559.1 0.639230069741041 6.19920273184478e-49 postive

CYLD AC090559.1 0.530700568915623 1.87429711409683e-31 postive

AXL AC090559.1 0.440106808670989 4.84601155849849e-21 postive

BRAF AF127577.4 0.437115563612146 9.52687225655415e-21 postive

BRAF AC009137.2 0.410739631994928 2.7860031705434e-18 postive

TRIM11 AC009065.4 0.420639727972633 3.50506942778696e-19 postive

BRAF PCAT1 0.573850571866805 1.25184844780673e-37 postive

TSC1 NUTM2B-AS1 0.409277956545012 3.76174631910629e-18 postive

MAPK8 NUTM2B-AS1 0.458406710765344 6.68061487596235e-23 postive

IPMK NUTM2B-AS1 0.415805870716707 9.72747997396674e-19 postive

OTULIN NUTM2B-AS1 0.433061740173626 2.35604586091048e-20 postive

BRAF NUTM2B-AS1 0.748394595724978 1.783683754257e-75 postive

ATRX NUTM2B-AS1 0.559161339544375 2.00255788859237e-35 postive

TSC1 AC006042.1 0.528487661043737 3.68177106066748e-31 postive

BRAF AC006042.1 0.460669139138944 3.86274170599818e-23 postive

GATA3 AC006042.1 0.474945221080767 1.1067224909556e-24 postive

TSC1 AL353804.1 0.400577697993031 2.18010144062884e-17 postive

MAPK8 AL353804.1 0.409849202214873 3.34580209311232e-18 postive

IPMK AL353804.1 0.427187438586443 8.56448199979223e-20 postive

OTULIN AL353804.1 0.464364839099316 1.56464948384116e-23 postive

BRAF AL353804.1 0.60211912732838 3.38049870086529e-42 postive

ATRX AL353804.1 0.705306985069592 1.53572484780494e-63 postive

TSC1 AP000766.1 0.4906491433766 1.82414263660116e-26 postive

MAPK8 AP000766.1 0.438569923319545 6.86404624235579e-21 postive

BRAF AP000766.1 0.715307568930503 4.11748602820565e-66 postive

SIRT1 AP000766.1 0.464575209985276 1.48570295911638e-23 postive

ATRX AP000766.1 0.630304996941179 3.13518116900748e-47 postive

TSC1 AC138393.3 0.424711688567066 1.46444618315744e-19 postive

MAPK8 AC138393.3 0.416742189166264 7.99265190182646e-19 postive

IPMK AC138393.3 0.450895236436994 4.0006894819816e-22 postive

OTULIN AC138393.3 0.447079525352652 9.76528634203106e-22 postive

BRAF AC138393.3 0.588427326833143 6.27821204709425e-40 postive

ATRX AC138393.3 0.696575860920479 2.2109372594966e-61 postive

CYLD AC079921.2 0.47968397603638 3.27855085083968e-25 postive

CFLAR AC079921.2 0.407725255292899 5.16676604868512e-18 postive

BRAF Z93403.1 0.595159895277454 4.96339591754522e-41 postive

OTULIN AC108727.1 0.409536776330711 3.56734176184244e-18 postive

BRAF AC108727.1 0.602890885616876 2.49929122271694e-42 postive

ATRX AC108727.1 0.631248936280643 2.08297278168876e-47 postive

TNFRSF1B AL133467.1 0.434198055040915 1.8301656388477e-20 postive

FLT3 AL133467.1 0.511762499183612 5.17463958512102e-29 postive

BACH2 AL133467.1 0.652088856771164 1.72136556865557e-51 postive

BRAF FLJ45513 0.489954642982473 2.19721419194777e-26 postive

TSC1 AC092794.1 0.417857562220151 6.32001609499943e-19 postive

MAPK8 AC092794.1 0.42452547508784 1.52447001277079e-19 postive

BRAF AC092794.1 0.684019096157696 2.06819547147627e-58 postive

ATRX AC092794.1 0.569900591597937 5.02481630307531e-37 postive

BRAF AC012085.2 0.599281848673182 1.01890414704338e-41 postive

ATRX AC012085.2 0.445282295988871 1.48094648034064e-21 postive

FLT3 AP001107.5 0.409598193288627 3.52268114523721e-18 postive

ATRX AC005479.1 0.476311028584407 7.80906440458315e-25 postive

TSC1 AL359915.2 0.51916404824839 5.99868463649384e-30 postive

BRAF AL359915.2 0.514899879419108 2.08929078029454e-29 postive

BRAF Z93930.3 0.565080671103968 2.67097150875457e-36 postive

MAPK8 AC125257.1 0.467847210311453 6.60996924079745e-24 postive

TSC1 AC124312.2 0.48934138161783 2.58868644166149e-26 postive

MAPK8 AC124312.2 0.459838329409073 4.72575341391027e-23 postive

BRAF AC124312.2 0.783066024265001 5.29346900278872e-87 postive

SIRT1 AC124312.2 0.442502885426863 2.80623266372517e-21 postive

ATRX AC124312.2 0.521983959517825 2.60278109455078e-30 postive

TSC1 AC011481.1 0.430319358667112 4.31737349399344e-20 postive

BRAF AC011481.1 0.417508989253855 6.8018531226133e-19 postive

BRAF AC119800.1 0.647488393709342 1.46112112644127e-50 postive

ATRX AC119800.1 0.41405241778605 1.40295189603648e-18 postive

TSC1 ZNF32-AS2 0.504187167822251 4.44894622361531e-28 postive

MAPK8 ZNF32-AS2 0.43898461420087 6.24970453852806e-21 postive

BRAF ZNF32-AS2 0.470268282067244 3.60986866548176e-24 postive

ATRX ZNF32-AS2 0.439947500618964 5.02449731535237e-21 postive

GATA3 AC005332.4 0.450154941977393 4.76109009364547e-22 postive

BRAF AC132807.2 0.516150940978178 1.45141318487042e-29 postive

BRAF AC107081.2 0.476110685076671 8.21969190093681e-25 postive

ATRX AL606534.1 0.568246400095675 8.9427996979482e-37 postive

IPMK AC087286.1 0.415732783145568 9.87752708991898e-19 postive

OTULIN AC087286.1 0.469754194987401 4.10628960652076e-24 postive

BRAF AC087286.1 0.571772240447487 2.60701763528302e-37 postive

ATRX AC087286.1 0.673002061897335 6.33141193137482e-56 postive

ATRX AL139383.1 0.472021074436772 2.32267131042468e-24 postive

TSC1 AC138932.5 0.453729704163746 2.04678148047126e-22 postive

BRAF AC138932.5 0.596823322043832 2.6270823669474e-41 postive

ATRX AC138932.5 0.599106330882792 1.09047933322056e-41 postive

STAT3 AC016876.3 -0.417319750902443 7.07839733701325e-19 negative

MAPK8 AL365436.2 0.424861147763041 1.41795828158345e-19 postive

OTULIN AL365436.2 0.40814215768393 4.74550103488414e-18 postive

BRAF AL365436.2 0.652409668419703 1.48080816389646e-51 postive

ATRX AL365436.2 0.520141973021304 4.49455238697271e-30 postive

MAP3K7 LINC01139 0.41152693126415 2.3685067680658e-18 postive

TSC1 AC244093.5 0.434511324098827 1.70677553326699e-20 postive

MAPK8 AC244093.5 0.42823774771855 6.81214461248202e-20 postive

IPMK AC244093.5 0.433081589299486 2.34569296666398e-20 postive

OTULIN AC244093.5 0.465565230353517 1.16387506703111e-23 postive

BRAF AC244093.5 0.672571692156769 7.87820718203877e-56 postive

ATRX AC244093.5 0.715688069014266 3.27035212066329e-66 postive

TSC1 AL442128.2 0.4737828951363 1.48724286724373e-24 postive

BRAF FRMD6-AS1 0.413625548448946 1.5332737444835e-18 postive

ATRX FRMD6-AS1 0.459431618278373 5.21500902434165e-23 postive

BRAF HID1-AS1 0.546851135501842 1.1640012016092e-33 postive

TSC1 STAM-AS1 0.600273996611735 6.93627019681768e-42 postive

BRAF STAM-AS1 0.441111157811944 3.85622340930358e-21 postive

TSC1 AC004951.1 0.456492500159705 1.05861458744451e-22 postive

MAPK8 AC004951.1 0.425050588464675 1.36111772707271e-19 postive

OTULIN AC004951.1 0.474666447958116 1.18813391528954e-24 postive

BRAF AC004951.1 0.644559890445233 5.59272330184975e-50 postive

ATRX AC004951.1 0.574100460515174 1.14575748443327e-37 postive

BRAF AL592043.1 0.61747125489092 7.09673176548226e-45 postive

ATRX AL592043.1 0.406171513279676 7.08633772790625e-18 postive

OTULIN AC121247.1 0.401408663129364 1.84738405799992e-17 postive

BRAF AC121247.1 0.628401086430446 7.12158676498583e-47 postive

ATRX AC121247.1 0.467986939494537 6.38402231922595e-24 postive

MAPK8 AC092794.2 0.447615114550813 8.6214618259304e-22 postive

OTULIN AC092794.2 0.451915621780428 3.14534100396706e-22 postive

BRAF AC092794.2 0.719215826287507 3.79505825552391e-67 postive

ATRX AC092794.2 0.553361209939058 1.38683920227729e-34 postive

BRAF AC008687.2 0.455105781077364 1.47496627465195e-22 postive

LEF1 LINC01852 0.504508889663223 4.06488045993819e-28 postive

GATA3 AC080129.2 0.449918163013551 5.03313513665186e-22 postive

ATRX COL4A2-AS1 0.46911327516611 4.82037738919563e-24 postive

RNF31 DHRS4-AS1 0.44382129423766 2.07381474787192e-21 postive

MAPK8 AL008718.3 0.421860651375463 2.70141501525063e-19 postive

OTULIN AL008718.3 0.494314461983091 6.78391552840338e-27 postive

BRAF AL008718.3 0.707579530392264 4.08777475298152e-64 postive

ATRX AL008718.3 0.60063429394342 6.03021760610421e-42 postive

BRAF AC008655.2 0.446005401678746 1.25286027843036e-21 postive

TSC1 AC008870.4 0.455338771523494 1.39516767281454e-22 postive

MAPK8 AC008870.4 0.452716903331946 2.60246955896172e-22 postive

IPMK AC008870.4 0.400164295537069 2.36690736834748e-17 postive

OTULIN AC008870.4 0.406084579221514 7.21235966366405e-18 postive

BRAF AC008870.4 0.597186922011806 2.28487606304294e-41 postive

ATRX AC008870.4 0.646055212772654 2.82338042510972e-50 postive

BRAF AC011472.4 0.535028830412775 4.93220335427348e-32 postive

TSC1 AC245884.8 0.493329536139292 8.85980805650263e-27 postive

BRAF AC245884.8 0.534110401225084 6.55798910038281e-32 postive

BRAF AL590133.1 0.407010648746382 5.97600789842503e-18 postive

ATRX AL590133.1 0.476673909677641 7.1162060097954e-25 postive

TSC1 RNF139-AS1 0.504825380409786 3.71907812692218e-28 postive

BRAF RNF139-AS1 0.573373946179406 1.48188812475994e-37 postive

GATA3 LINC00900 0.450988579813153 3.91374883491467e-22 postive

TSC1 DLEU1 0.404574724894127 9.7876911057699e-18 postive

MAPK8 DLEU1 0.446451276152678 1.1298675059317e-21 postive

IPMK DLEU1 0.426548376802972 9.84060646819604e-20 postive

OTULIN DLEU1 0.50434037659794 4.26177239133046e-28 postive

BRAF DLEU1 0.790532938164949 9.1175905341199e-90 postive

ATRX DLEU1 0.586134724323778 1.46979048218484e-39 postive

BRAF AC019186.1 0.519129345743194 6.06034595998148e-30 postive

ATRX AC019186.1 0.417816281383493 6.37527695123506e-19 postive

TSC1 AP005057.1 0.420659075317427 3.49066277177117e-19 postive

OTULIN AP005057.1 0.4038951839806 1.12238305185469e-17 postive

BRAF AP005057.1 0.622218175809736 9.8357301026017e-46 postive

ATRX AP005057.1 0.506017669799661 2.65834335914021e-28 postive

MAPK8 AC103769.1 0.416167181162918 9.01804600405028e-19 postive

OTULIN AC103769.1 0.438225915106856 7.41852895496778e-21 postive

BRAF AC103769.1 0.647294426308566 1.59769899013784e-50 postive

ATRX AC103769.1 0.528956523375701 3.19235370822556e-31 postive

TSC1 AL157871.5 0.408180532851728 4.70846672275844e-18 postive

MAPK8 AL157871.5 0.422255618658892 2.48257933013672e-19 postive

OTULIN AL157871.5 0.435962460524111 1.23407995913894e-20 postive

BRAF AL157871.5 0.602038605166443 3.48855294811007e-42 postive

ATRX AL157871.5 0.652842489426186 1.20829640553844e-51 postive

TSC1 AL391684.1 0.490201481813477 2.05669948313616e-26 postive

BRAF AL391684.1 0.555907418095051 5.9581069925577e-35 postive

BRAF AL353801.3 0.567426332953137 1.18867545527895e-36 postive

TSC1 AC005519.1 0.53536932291343 4.43684004766355e-32 postive

MAPK8 AC005519.1 0.403029492331744 1.33565202379784e-17 postive

BRAF AC005519.1 0.573143428184219 1.60770424227436e-37 postive

ATRX AC005519.1 0.58355485062264 3.79723210229651e-39 postive

TSC1 AC093535.1 0.454655183534802 1.6422753169224e-22 postive

MAPK8 AC093535.1 0.454674253033141 1.63482975239674e-22 postive

OTULIN AC093535.1 0.42121217432695 3.10258044440955e-19 postive

BRAF AC093535.1 0.820851009024681 2.87174170271298e-102 postive

ATRX AC093535.1 0.550299248759134 3.79412205508743e-34 postive

TSC1 AC011442.1 0.469944642131488 3.9149887096387e-24 postive

MAPK8 AC011442.1 0.422802399042572 2.20817427361786e-19 postive

BRAF AC011442.1 0.677129502446645 7.63638926116968e-57 postive

ATRX AC011442.1 0.556484300752366 4.9151623703994e-35 postive

STAT3 SNHG10 -0.456627750009032 1.02483429572622e-22 negative

BRAF AC004466.3 0.542356935170649 4.92137682210752e-33 postive

ATRX AC004466.3 0.557203728126503 3.86446075232037e-35 postive

TSC1 AC110769.2 0.488076466120575 3.62658906194526e-26 postive

MAPK8 AC110769.2 0.465844231477937 1.08633673547057e-23 postive

OTULIN AC110769.2 0.469170570951938 4.75184457665355e-24 postive

BRAF AC110769.2 0.652379794026179 1.50172321333839e-51 postive

ATRX AC110769.2 0.639770286568403 4.8684100464943e-49 postive

TSC1 C10orf111 0.400740072455127 2.1107615016339e-17 postive

MAPK8 C10orf111 0.46055452733312 3.97183422742232e-23 postive

BRAF C10orf111 0.457001104714169 9.37004801184569e-23 postive

LEF1 AC069120.1 0.575464571247413 7.05614226283874e-38 postive

KLF9 AL583785.1 0.412643330969622 1.88007456917408e-18 postive

IPMK AC098484.1 0.437507348918815 8.72298606291359e-21 postive

BRAF AC098484.1 0.479283117085678 3.63660365798157e-25 postive

ATRX AC098484.1 0.42997237372708 4.65940335217466e-20 postive

TSC1 AC079160.1 0.507730907240118 1.63705932860973e-28 postive

BRAF AC079160.1 0.75332168918573 5.38172150792621e-77 postive

ATRX AC079160.1 0.466102157918389 1.01920090305594e-23 postive

OTULIN AL157400.4 0.423934968237193 1.73128747102158e-19 postive

BRAF AL157400.4 0.557230688868869 3.82974363000498e-35 postive

ATRX AL157400.4 0.4977679254998 2.64216744244157e-27 postive

KLF9 LINC01936 0.499004994960058 1.87987344481623e-27 postive

BRAF AC104126.1 0.403574967542282 1.19705285564592e-17 postive

TSC1 AL138902.1 0.487729340574884 3.97715972874888e-26 postive

BRAF AL138902.1 0.539275343186314 1.30615841460451e-32 postive

ATRX AL138902.1 0.411273864619364 2.49550508172546e-18 postive

TRAF2 AL390729.1 0.458297908387939 6.85825542749223e-23 postive

OTULIN AC022001.3 0.416327970799683 8.71894526372783e-19 postive

BRAF AC022001.3 0.533309282831746 8.40208943680484e-32 postive

ATRX AC022001.3 0.569703907273977 5.38222053397971e-37 postive

BRAF AL161729.3 0.494842271493791 5.87749995743813e-27 postive

TRAF2 AL441992.1 0.545379681232445 1.87067171077209e-33 postive

CDKN2A AL441992.1 0.431833390284826 3.0924114541889e-20 postive

BRAF AC006460.1 0.463539830208023 1.91622453496963e-23 postive

ATRX AC006460.1 0.452125641621586 2.9931068662174e-22 postive

BRAF AC005841.1 0.431288851932623 3.4874191429126e-20 postive

TRIM11 AC005785.1 0.426730031705862 9.45994441329273e-20 postive

TSC1 Z98885.3 0.474269136384702 1.31447153247442e-24 postive

MAPK8 Z98885.3 0.448727231469572 6.65167718071074e-22 postive

IPMK Z98885.3 0.427012374663749 8.89688684109069e-20 postive

OTULIN Z98885.3 0.517579809725468 9.5564048289995e-30 postive

BRAF Z98885.3 0.733103567273014 5.65750737418909e-71 postive

ATRX Z98885.3 0.714212821072716 7.97149019551806e-66 postive

ATRX AC234772.2 0.486874434764315 4.9897019839605e-26 postive

TRIM11 AC010618.2 0.436284516213645 1.1481423809829e-20 postive

TSC1 AL049780.1 0.434309679698464 1.78522297832416e-20 postive

BRAF AL049780.1 0.530272870504799 2.1363802846904e-31 postive

ATRX AL049780.1 0.495493199509379 4.92295826357402e-27 postive

BRAF AL359878.2 0.445134809671993 1.53226267432594e-21 postive

BCL2 AL121929.2 0.405975497624901 7.373608543265e-18 postive

TSC1 AC073896.3 0.409095265584734 3.90520534234677e-18 postive

BRAF AC073896.3 0.456225934207262 1.12843957861566e-22 postive

ATRX AC073896.3 0.47416336224631 1.35028363093644e-24 postive

FASLG LINC02361 0.416649202036767 8.15032749099492e-19 postive

TNFRSF1B LINC02361 0.451711056727981 3.30095276400002e-22 postive

TSC1 AL031666.3 0.434955114338834 1.54588924888312e-20 postive

MAPK8 AL031666.3 0.475095099225211 1.06525496009878e-24 postive

OTULIN AL031666.3 0.507068199092384 1.97537044796496e-28 postive

BRAF AL031666.3 0.823207035456165 2.4446707679883e-103 postive

ATRX AL031666.3 0.598754823166586 1.24913762239367e-41 postive

RNF31 AL118506.1 0.439021063231104 6.19837270969457e-21 postive

BRAF IQCH-AS1 0.453217175469906 2.31155002525984e-22 postive

MAPK8 AC108704.1 0.410382136398122 2.9987194809707e-18 postive

BRAF AC108704.1 0.496598697514458 3.64025768574968e-27 postive

ATRX AC108704.1 0.400574440654144 2.18151510966118e-17 postive

GATA3 AL390719.2 0.401384688256172 1.8562438667716e-17 postive

TSC1 AC092279.1 0.500854622846067 1.12711427870413e-27 postive

MAPK8 AC092279.1 0.486539120582136 5.45297699324383e-26 postive

OTULIN AC092279.1 0.417296334402102 7.11337727144972e-19 postive

BRAF AC092279.1 0.810939979678786 6.20109923479837e-98 postive

ATRX AC092279.1 0.542604636235237 4.54801687734831e-33 postive

BRAF AP003086.2 0.449710647791924 5.28412109792526e-22 postive

TSC1 LINC01572 0.445792985388724 1.31600664800095e-21 postive

MAPK8 LINC01572 0.477564372361943 5.66277606721529e-25 postive

OTULIN LINC01572 0.484456539247616 9.4442517990674e-26 postive

BRAF LINC01572 0.722685493242972 4.4168519114771e-68 postive

ATRX LINC01572 0.586846775745891 1.12936254725084e-39 postive

ATRX AL122035.1 0.415230867357555 1.09712844286467e-18 postive

TSC1 AC107419.1 0.424822143974612 1.42994802393082e-19 postive

MAPK8 AC107419.1 0.415667840656922 1.00127619361536e-18 postive

OTULIN AC107419.1 0.440219592070807 4.72344335394822e-21 postive

BRAF AC107419.1 0.755090920114138 1.50027074540167e-77 postive

ATRX AC107419.1 0.510040642778149 8.47851268036523e-29 postive

ATRX AL158166.2 0.429390728794366 5.29355733154427e-20 postive

LEF1 AL158166.2 0.417674490348669 6.56873263578197e-19 postive

ATRX AC008966.2 0.545594665486273 1.74565065046558e-33 postive

BRAF AC040169.3 0.460255943078612 4.27051727659625e-23 postive

ATRX AC040169.3 0.444832279292636 1.64307155502355e-21 postive

TSC1 AL592430.1 0.45921108903763 5.50084873695677e-23 postive

MAPK8 AL592430.1 0.470535767691763 3.37550105985475e-24 postive

OTULIN AL592430.1 0.460941503312921 3.61521108852416e-23 postive

BRAF AL592430.1 0.824184138948205 8.70593826572925e-104 postive

ATRX AL592430.1 0.556700483490765 4.57276009910923e-35 postive

LEF1 SH3RF3-AS1 0.407812821089182 5.07533761375215e-18 postive

ATRX AC098829.1 0.5056884587641 2.91697656942432e-28 postive

LEF1 LINC01405 0.507970000721125 1.52963018154667e-28 postive

TRIM11 AC018809.1 0.472208138402103 2.21556530695117e-24 postive

HAT1 AL021707.8 -0.407913610595085 4.97207079733985e-18 negative

TSC1 NARF-IT1 0.444546062981143 1.75515971910599e-21 postive

MAPK8 NARF-IT1 0.427474820604894 8.04513619807109e-20 postive

BRAF NARF-IT1 0.594942437238733 5.39238841998707e-41 postive

ATRX NARF-IT1 0.513438188628222 3.19164735580543e-29 postive

TSC1 AL121832.3 0.50576159272273 2.85745405720259e-28 postive

BRAF AL121832.3 0.518821697274529 6.63512874876906e-30 postive

TSC1 AL669831.5 0.566313764648131 1.74655989090467e-36 postive

MAPK8 AL669831.5 0.477749120879509 5.40017091650422e-25 postive

OTULIN AL669831.5 0.431227474074881 3.53494570558325e-20 postive

BRAF AL669831.5 0.757499937890054 2.58909000308228e-78 postive

ATRX AL669831.5 0.629127854291785 5.21021470999524e-47 postive

KLF9 FENDRR 0.422007801861202 2.61775064421771e-19 postive

TSC1 AC108062.1 0.504006465159152 4.68017167103726e-28 postive

BRAF AC108062.1 0.562068921956381 7.48209239580054e-36 postive

TSC1 AL450384.1 0.484589610042807 9.11957856055421e-26 postive

MAPK8 AL450384.1 0.435736336823334 1.2981750108077e-20 postive

OTULIN AL450384.1 0.423910290632298 1.74050694631215e-19 postive

BRAF AL450384.1 0.812659851187473 1.14524849618702e-98 postive

ATRX AL450384.1 0.535003087370866 4.9718064697504e-32 postive

OTULIN LINC01146 0.433254186750549 2.25753829196978e-20 postive

BRAF LINC01146 0.640544481827454 3.44045428998595e-49 postive

TSC1 STX18-AS1 0.431965159221614 3.00365407503773e-20 postive

MAPK8 STX18-AS1 0.410797996354611 2.75271436119129e-18 postive

BRAF STX18-AS1 0.511277026982098 5.94927819458623e-29 postive

ATRX STX18-AS1 0.53712735320404 2.56390946035562e-32 postive

BRAF AB015752.1 0.608104037235368 3.17962671109832e-43 postive

CYLD AC023825.2 0.423543519469757 1.88336676058842e-19 postive

ATRX AC023825.2 0.526305113983767 7.13091586756226e-31 postive

GATA3 CEBPA-DT 0.43760290630888 8.53728526844932e-21 postive

IDH1 CEBPA-DT 0.476443089969575 7.54953212969822e-25 postive

SIRT1 LMF1-AS1 0.400798246496674 2.08645032325968e-17 postive

TSC1 PWAR6 0.455998327811944 1.19164008539073e-22 postive

MAPK8 PWAR6 0.402592299950981 1.45802512030134e-17 postive

BRAF PWAR6 0.747644533530786 3.01764273251689e-75 postive

ATRX PWAR6 0.437798234372002 8.16973111399434e-21 postive

MAP3K7 AC020928.1 0.566077016782757 1.89523890765134e-36 postive

TSC1 AC008764.8 0.570157151854879 4.59371711890566e-37 postive

BRAF AC008764.8 0.438000876311406 7.80489432289892e-21 postive

BRAF AC009948.3 0.534529846647399 5.75851536393564e-32 postive

ATRX AC009948.3 0.428859821382226 5.94617082989595e-20 postive

BRAF AC012170.3 0.516733338857347 1.22438468558951e-29 postive

ATRX AC012170.3 0.538746218512655 1.54292349491024e-32 postive

ATRX LINC00954 0.419156406068134 4.80276729931791e-19 postive

ATRX AC093484.4 0.479795401669767 3.1853631559878e-25 postive

TSC1 CAPN10-DT 0.543272977134457 3.6747537277179e-33 postive

BRAF CAPN10-DT 0.448630675335104 6.80343181594939e-22 postive

TNFRSF1B AL109741.1 0.439479427157225 5.58724788349188e-21 postive

FLT3 AL109741.1 0.463982610013391 1.71882252116654e-23 postive

KLF9 AL109741.1 0.664931940028488 3.59101567909084e-54 postive

BRAF AP001033.2 0.457874695563469 7.59463723826287e-23 postive

LEF1 LINC01411 0.479581269506983 3.36682779627167e-25 postive

TSC1 GTF3C2-AS1 0.485794534307959 6.63902865277205e-26 postive

MAPK8 GTF3C2-AS1 0.480125515424737 2.92436097194876e-25 postive

OTULIN GTF3C2-AS1 0.463645445486292 1.86719841639122e-23 postive

BRAF GTF3C2-AS1 0.811983893436348 2.22902052040083e-98 postive

ATRX GTF3C2-AS1 0.628178137233923 7.83681466405901e-47 postive

TSC1 AC010618.3 0.426879204949053 9.15822173467416e-20 postive

GATA3 AC010326.3 0.460402699557131 4.12104240131703e-23 postive

OTULIN AC104170.2 0.426815331937313 9.28623532429673e-20 postive

BRAF AC104170.2 0.52322713707062 1.79677766923914e-30 postive

ATRX AC104170.2 0.617497663710417 7.01978995416766e-45 postive

TSC1 AC004076.2 0.48234281237397 1.64287186319413e-25 postive

BRAF AC004076.2 0.54997178434145 4.22269209020374e-34 postive

ATRX AC004076.2 0.429497881770836 5.17066579385784e-20 postive

TSC1 AC027601.3 0.460104038328591 4.43086809645624e-23 postive

TSC1 HMGA1P4 0.440290412266106 4.64804575785705e-21 postive

BRAF HMGA1P4 0.483105496449752 1.34598797769217e-25 postive

TSC1 AC020978.1 0.404176447260212 1.06058789064159e-17 postive

MAPK8 AC020978.1 0.432154408962233 2.88055245697051e-20 postive

OTULIN AC020978.1 0.4471483688668 9.61027724596006e-22 postive

BRAF AC020978.1 0.675038885699611 2.23898097496537e-56 postive

ATRX AC020978.1 0.612806080989374 4.79046486184779e-44 postive

OTULIN AL162734.1 0.407107222219673 5.85976346599561e-18 postive

BRAF AL162734.1 0.624702141192732 3.44983183406316e-46 postive

ATRX AL162734.1 0.492669520081784 1.05903476589037e-26 postive

TSC1 AC090579.1 0.438234215898728 7.40464381935946e-21 postive

BRAF AC090579.1 0.75044688286971 4.19137748745807e-76 postive

ATRX AC090579.1 0.471026958670355 2.98357315980143e-24 postive

TSC1 AC139100.2 0.465017346824329 1.33236832932617e-23 postive

BRAF AC139100.2 0.541905212210164 5.68198905387351e-33 postive

TSC1 AC106864.1 0.539677911746781 1.1504448536763e-32 postive

MAPK8 AC106864.1 0.417513316254611 6.79565582401608e-19 postive

BRAF AC106864.1 0.655699599224539 3.12947747104147e-52 postive

ATRX AC106864.1 0.608955467676301 2.26229968039144e-43 postive

TNFRSF1B AC090825.1 0.454039170765961 1.90163948994146e-22 postive

FLT3 AC090825.1 0.548539439198219 6.73419792004761e-34 postive

KLF9 AC090825.1 0.478298969844349 4.68768503779648e-25 postive

TSC1 AC097641.2 0.499001268977024 1.88180569793882e-27 postive

MAPK8 AC097641.2 0.416329930532122 8.71536066677677e-19 postive

BRAF AC097641.2 0.72229927253929 5.62076234249118e-68 postive

ATRX AC097641.2 0.503691235925244 5.11230399399978e-28 postive

KLF9 BX322562.1 0.439200638884398 5.95147627746612e-21 postive

LEF1 BX322562.1 0.445797552098386 1.31461663342179e-21 postive

TSC1 AC022893.3 0.450605149228642 4.28321469561767e-22 postive

MAPK8 AC022893.3 0.424828775536423 1.42790246608564e-19 postive

OTULIN AC022893.3 0.459862443788885 4.69821127367705e-23 postive

BRAF AC022893.3 0.755385630114635 1.21143844130557e-77 postive

ATRX AC022893.3 0.629341049310462 4.75306631179035e-47 postive

BNIP3 LINC01550 0.506686557975644 2.20064698963087e-28 postive

BRAF AC016571.1 0.599798974841279 8.33985929529042e-42 postive

TSC1 AL359220.1 0.47891471141833 3.99957102049883e-25 postive

MAPK8 AL359220.1 0.445420068294978 1.43454233979096e-21 postive

BRAF AL359220.1 0.737303364789285 3.52956041958079e-72 postive

ATRX AL359220.1 0.566837561911343 1.45741163963465e-36 postive

KLF9 HAND2-AS1 0.517184982725024 1.07283221890049e-29 postive

TSC1 AL512343.2 0.491531433002303 1.43921897431341e-26 postive

MAPK8 AL512343.2 0.536141420628654 3.48855179198018e-32 postive

OTULIN AL512343.2 0.439766866129356 5.23472189448754e-21 postive

BRAF AL512343.2 0.734351528014921 2.49453058011252e-71 postive

ATRX AL512343.2 0.557924749419409 3.03496093020222e-35 postive

BRAF AC010789.1 0.483867836974847 1.10230148561039e-25 postive

BACH2 LINC02202 0.508329562604706 1.38105828549276e-28 postive

KLF9 LINC02202 0.434519683492308 1.70359778516676e-20 postive

BRAF AC026704.1 0.530563938148866 1.9543626263389e-31 postive

TSC1 UBOX5-AS1 0.472862968093259 1.87763991011243e-24 postive

BRAF UBOX5-AS1 0.633527255963609 7.7185047386017e-48 postive

ATRX UBOX5-AS1 0.532614005622209 1.04124564157021e-31 postive

TSC1 AC005828.4 0.456703118793854 1.00647364894667e-22 postive

MAPK8 AC005828.4 0.448459186375463 7.08141620196173e-22 postive

BRAF AC005828.4 0.781040946488291 2.84753563429991e-86 postive

ATRX AC005828.4 0.471891516015193 2.39983095665463e-24 postive

BRAF BX284668.6 0.43822376062911 7.42213704555648e-21 postive

ATRX AC025280.1 0.496862649334216 3.38657807359635e-27 postive

MAPK8 AC004918.3 0.41439910919931 1.30518193627175e-18 postive

IPMK AC004918.3 0.404096680306664 1.07776499969809e-17 postive

OTULIN AC004918.3 0.436089083844736 1.19956091811074e-20 postive

BRAF AC004918.3 0.656482431313422 2.15577166550616e-52 postive

ATRX AC004918.3 0.648326543995641 9.92349411470159e-51 postive

ATRX AP003486.1 0.425550230922456 1.2217436821333e-19 postive

OTULIN AC004943.2 0.526781946052497 6.17449408096811e-31 postive

DNMT1 AC004943.2 0.424477756487468 1.54023744247748e-19 postive

TSC1 AL592211.1 0.46827201755369 5.94641571322515e-24 postive

BRAF AL135905.1 0.418599985145275 5.40295381621346e-19 postive

TSC1 AC002128.1 0.59471797142353 5.87374230733052e-41 postive

MAPK8 AC002128.1 0.429725449811821 4.91891743940056e-20 postive

BRAF AC002128.1 0.739084433337983 1.07083458824442e-72 postive

ATRX AC002128.1 0.532229269947802 1.17223410217714e-31 postive

BRAF AC005332.7 0.42448198001819 1.5388354291444e-19 postive

OTULIN AC005730.3 0.463501372976869 1.9343898115821e-23 postive

BRAF AC005730.3 0.531636872805989 1.40644517206432e-31 postive

ATRX AC005730.3 0.603616115078478 1.88033196603177e-42 postive

GATA3 LINC00910 0.437051314620404 9.66549266818831e-21 postive

IDH1 LINC00910 0.401434698002675 1.83781006619959e-17 postive

TSC1 AC024361.1 0.532580908982861 1.05191998044395e-31 postive

BRAF AC024361.1 0.681540296664915 7.66406098396762e-58 postive

TSC1 LENG8-AS1 0.471689644414282 2.52512730064757e-24 postive

TSC1 AL136531.1 0.414135783064962 1.37880380907401e-18 postive

BRAF AL136531.1 0.521872770026167 2.69029591696422e-30 postive

MAP3K7 NIFK-AS1 0.47755061355239 5.68282999187419e-25 postive

TSC1 AC127024.5 0.435804756596393 1.27844290709331e-20 postive

BRAF AC127024.5 0.4866200655314 5.33739557177114e-26 postive

TSC1 AC084018.1 0.416064133724125 9.21502002923288e-19 postive

DIABLO AC084018.1 0.408478216492755 4.43067825915984e-18 postive

TSC1 AL442647.1 0.42070622886759 3.45579440822772e-19 postive

BRAF AL442647.1 0.751150865068386 2.54209716417132e-76 postive

ATRX AL442647.1 0.496630087688524 3.60913220725109e-27 postive

TSC1 AC005391.1 0.417336931390516 7.05284057159171e-19 postive

MAPK8 AC005391.1 0.418596342425801 5.40711664284927e-19 postive

OTULIN AC005391.1 0.440039927773656 4.9201696571663e-21 postive

BRAF AC005391.1 0.698413374222313 7.88586630232179e-62 postive

ATRX AC005391.1 0.562174613411501 7.21774379590207e-36 postive

TSC1 AL031666.2 0.405659730090872 7.86064056561361e-18 postive

MAPK8 AL031666.2 0.452738999338281 2.58888988766121e-22 postive

OTULIN AL031666.2 0.531647381084096 1.40191246022511e-31 postive

BRAF AL031666.2 0.779110045005295 1.39309571167509e-85 postive

ATRX AL031666.2 0.573128450462204 1.61623562625063e-37 postive

TSC1 AC144548.1 0.461257184834292 3.34784230803273e-23 postive

BRAF AC144548.1 0.422192793293765 2.51618264622178e-19 postive

ATRX AC144548.1 0.539752924141242 1.1235307638985e-32 postive

BRAF AC008496.2 0.456192788888227 1.13743321361675e-22 postive

TSC1 AC073534.1 0.404637180635165 9.66514661895016e-18 postive

BRAF AC073534.1 0.758585808833543 1.16488448054894e-78 postive

ATRX AC073534.1 0.402186458999943 1.5814530109703e-17 postive

BRAF AC091057.4 0.405789872328232 7.65619506906437e-18 postive

ATRX AC091057.4 0.538277556475817 1.78779984566362e-32 postive

OTULIN AC009041.4 0.458464329849931 6.58838685746453e-23 postive

BRAF AC009041.4 0.431428326647378 3.38174760055046e-20 postive

ATRX AC009041.4 0.478827272241602 4.09084048597263e-25 postive

FLT3 LINC00892 0.42805494178784 7.08944712869124e-20 postive

BACH2 LINC00892 0.522563353279714 2.19033843166109e-30 postive

BRAF AC020663.3 0.558531952955278 2.47504550803844e-35 postive

TRIM11 AC069281.2 0.420073809009327 3.95336360216803e-19 postive

OTULIN AC022784.6 0.422124284752576 2.55333614905392e-19 postive

BRAF AC022784.6 0.756363414589053 5.94613576713313e-78 postive

TSC1 KDM4A-AS1 0.579998768561757 1.38567621338341e-38 postive

BRAF KDM4A-AS1 0.678005180990047 4.85355371682278e-57 postive

ATRX KDM4A-AS1 0.465596944425494 1.15479329120115e-23 postive

BRAF AC010175.1 0.446584676241857 1.09543998908327e-21 postive

DDX58 U62317.1 0.597416084771683 2.09229129895115e-41 postive

TERT AC026740.1 0.542635179885509 4.50396478563859e-33 postive

TSC1 AC092910.3 0.615985082600043 1.30845803724644e-44 postive

MAPK8 AC092910.3 0.463610567474716 1.88325018984026e-23 postive

BRAF AC092910.3 0.599996347535459 7.72540369279819e-42 postive

ATRX AC092910.3 0.520661141408432 3.85446321476266e-30 postive

BRAF AC079684.1 0.413334448123017 1.62890332407049e-18 postive

GATA3 AC084125.2 0.415836643244908 9.66497773045653e-19 postive

TSC1 AC007038.2 0.403050759092226 1.32996415813005e-17 postive

BRAF AC011479.2 0.484352551287076 9.70588877195116e-26 postive

ATRX AC011479.2 0.474838337421008 1.13726387241161e-24 postive

BRAF AC005537.1 0.504562587916388 4.00404849307762e-28 postive

TSC1 PPP1R26-AS1 0.50973682726926 9.24762695331255e-29 postive

IDH1 AATBC 0.40882359035355 4.12854385869527e-18 postive

TSC1 AC091906.1 0.406249089334725 6.97571137984546e-18 postive

MAPK8 AC091906.1 0.49059119574584 1.85271566687769e-26 postive

OTULIN AC091906.1 0.485249811708891 7.66479685120903e-26 postive

BRAF AC091906.1 0.800762313887086 9.65346241193495e-94 postive

ATRX AC091906.1 0.54723997680836 1.02644237912426e-33 postive

TSC1 AC020911.1 0.482982952415372 1.38983816085291e-25 postive

BRAF AC020911.1 0.453521841838067 2.15034843137379e-22 postive

ATRX AC020911.1 0.460666618784274 3.86510859939287e-23 postive

BRAF AL137145.2 0.424155006552542 1.65117909932198e-19 postive

ATRX AL137145.2 0.555255678299351 7.4016251613465e-35 postive

FASLG AL365361.1 0.491248573420696 1.55295588556511e-26 postive

TNFRSF1B AL365361.1 0.615903753526395 1.35287953311387e-44 postive

CYLD AL365361.1 0.455851926950847 1.23412362749236e-22 postive

FLT3 AL365361.1 0.737111211016176 4.01193186234166e-72 postive

BACH2 AL365361.1 0.457038384439459 9.28653742782058e-23 postive

MAPK8 LINC02163 0.476018085499178 8.41661855158756e-25 postive

BRAF LINC02163 0.620495404392241 2.02295956465565e-45 postive

ATRX LINC02163 0.455533080945426 1.33188537360776e-22 postive

TRIM11 AL691482.3 0.411126577973307 2.5724784641693e-18 postive

GATA3 AL691482.3 0.402129442789974 1.59959655243702e-17 postive

TSC1 AC100823.1 0.457018853997994 9.33019574526248e-23 postive

BRAF AC100823.1 0.728972706217182 8.23448971059678e-70 postive

ATRX AC100823.1 0.511383279624582 5.77049223836774e-29 postive

BRAF AC011465.1 0.450204615101508 4.70588585524696e-22 postive

ATRX AC011465.1 0.487355149052829 4.39259518681188e-26 postive

TSC1 AL139021.2 0.473293964539467 1.68353760532469e-24 postive

MAPK8 AL139021.2 0.410834685118837 2.73198910044199e-18 postive

BRAF AL139021.2 0.705104538512395 1.72684850622851e-63 postive

ATRX AL139021.2 0.58664105941331 1.21876425095207e-39 postive

STAT3 MHENCR -0.408378138199423 4.52222020272005e-18 negative

FASLG LINC00861 0.442343557771085 2.91044112715907e-21 postive

TNFRSF1B LINC00861 0.562747846478426 5.93714483470634e-36 postive

FLT3 LINC00861 0.759346151860564 6.64212517525822e-79 postive

BACH2 LINC00861 0.418066830472888 6.04704889797425e-19 postive

MAP3K7 A2M-AS1 0.546439129980323 1.32968339302539e-33 postive

TSC1 AC025178.1 0.476111507472554 8.21796349293083e-25 postive

OTULIN AC025178.1 0.454809528312787 1.58295958376981e-22 postive

BRAF AC025178.1 0.618876085464477 3.96817525144682e-45 postive

TSC1 AC004477.1 0.468513917773803 5.59840521452072e-24 postive

BRAF AC004477.1 0.406071748948181 7.23114444981552e-18 postive

TSC1 SLFNL1-AS1 0.408684833273029 4.24741271804016e-18 postive

BRAF SLFNL1-AS1 0.464993163093658 1.34033601682263e-23 postive

BRAF AC098820.1 0.429122806632449 5.61356028689129e-20 postive

TSC1 AC245052.4 0.501730471088908 8.83680844888828e-28 postive

BRAF JARID2-AS1 0.426961004830911 8.99681435414064e-20 postive

ATRX JARID2-AS1 0.47987597333819 3.11961324544809e-25 postive

TSC1 AC005899.7 0.437928841509507 7.93270863085339e-21 postive

BRAF AC005899.7 0.581151904842209 9.12249913095846e-39 postive

ATRX AC005899.7 0.474025497595101 1.39840964987115e-24 postive

OTULIN AL031710.2 0.500290874106007 1.31772240011542e-27 postive

BRAF AL031710.2 0.620737538852697 1.82846859687056e-45 postive

ATRX AL031710.2 0.463408899854847 1.97876782534736e-23 postive

TSC1 AC055713.1 0.423509314847603 1.89726384987088e-19 postive

BRAF LINC02466 0.564330963706437 3.45506810659829e-36 postive

MAPK8 BACH1-IT2 0.400912156863397 2.03964120134386e-17 postive

OTULIN BACH1-IT2 0.474110724591051 1.3684624087508e-24 postive

BRAF BACH1-IT2 0.57564374530548 6.61975317850344e-38 postive

ATRX BACH1-IT2 0.63776531432355 1.19080579107888e-48 postive

BRAF FIRRE 0.620079722733167 2.40581433459032e-45 postive

ATRX FIRRE 0.512734401629579 3.91110635024588e-29 postive

TSC1 AC078852.1 0.420165367289323 3.87718773961155e-19 postive

MAPK8 AC078852.1 0.430723666982297 3.94994208042814e-20 postive

OTULIN AC078852.1 0.489183116394266 2.70042822024766e-26 postive

BRAF AC078852.1 0.752976328429473 6.89702184436294e-77 postive

ATRX AC078852.1 0.592842094970489 1.19703505370941e-40 postive

TSC1 AC124312.5 0.442219347226475 2.9943184116628e-21 postive

MAPK8 AC124312.5 0.437113051301133 9.53225570751705e-21 postive

OTULIN AC124312.5 0.410965827975714 2.65915457583397e-18 postive

BRAF AC124312.5 0.771144203005066 8.24979544487632e-83 postive

ATRX AC124312.5 0.509862869557211 8.92051840038271e-29 postive

TSC1 AL139397.1 0.536036115533833 3.60498842317141e-32 postive

MAPK8 AL139397.1 0.412361344292526 1.9931719928817e-18 postive

BRAF AL139397.1 0.663844856683684 6.12735853756204e-54 postive

ATRX AL139397.1 0.40293346745933 1.36163340971851e-17 postive

FLT3 LINC01081 0.42693790213203 9.04211454185112e-20 postive

KLF9 LINC01081 0.55505573798582 7.91024429854632e-35 postive

HDAC9 AC026250.1 0.425695051909489 1.18404186097319e-19 postive

KLF9 AC036108.3 0.453896492281998 1.96725011128454e-22 postive

TSC1 AL356299.2 0.424959962285269 1.38802400056025e-19 postive

BRAF AL356299.2 0.522128403823184 2.49328415822495e-30 postive

ATRX AL356299.2 0.458631173928226 6.32835243493836e-23 postive

BRAF LINC00649 0.434395215062888 1.75152185915651e-20 postive

ATRX LINC00649 0.419774513626534 4.21279922812138e-19 postive

TSC1 AC010201.2 0.562356482518267 6.78433643724721e-36 postive

BRAF AC010201.2 0.644514641166547 5.70926800972491e-50 postive

ATRX AC010201.2 0.41395779176601 1.43086630727684e-18 postive

MAPK8 TBL1XR1-AS1 0.464385624762392 1.55666843815764e-23 postive

IPMK TBL1XR1-AS1 0.412346086988702 1.99947858475616e-18 postive

OTULIN TBL1XR1-AS1 0.501129108051526 1.04444046560903e-27 postive

BRAF TBL1XR1-AS1 0.754385943523119 2.49910870303222e-77 postive

ATRX TBL1XR1-AS1 0.618141444335814 5.37991291887481e-45 postive

TSC1 AL583810.1 0.421305654923931 3.04132505639907e-19 postive

OTULIN AL442125.2 0.429242903271673 5.46782882582991e-20 postive

ATRX AL442125.2 0.478566457611038 4.37547073649751e-25 postive

KLF9 MIR100HG 0.57644309913598 4.97669565028657e-38 postive

KLF9 RASSF8-AS1 0.410622856695337 2.85380055527084e-18 postive

MAPK8 AC005021.1 0.414035396837306 1.40793319740722e-18 postive

OTULIN AC005021.1 0.415950522340647 9.43709567992958e-19 postive

BRAF AC005021.1 0.652443312413294 1.45759978871266e-51 postive

ATRX AC005021.1 0.58117969799171 9.03086188579229e-39 postive

TSC1 AL162727.2 0.484357706266124 9.69275169782748e-26 postive

MAPK8 AL162727.2 0.438289838441434 7.31225974577501e-21 postive

OTULIN AL162727.2 0.434530105857028 1.69964397964391e-20 postive

BRAF AL162727.2 0.732187541604656 1.02894935839368e-70 postive

ATRX AL162727.2 0.629858326006817 3.80258758567681e-47 postive

TSC1 AF117829.1 0.508742408052383 1.22799524409736e-28 postive

BRAF AF117829.1 0.648436532717216 9.43139985694194e-51 postive

ATRX AF117829.1 0.53764209066753 2.18224206801011e-32 postive

MAP3K7 AC092376.2 0.561077610472439 1.04778022247217e-35 postive

TSC1 AC008750.5 0.502051642197761 8.08107423300561e-28 postive

OTULIN AC008750.5 0.422646848538556 2.28304488412632e-19 postive

BRAF AC008750.5 0.596704613917617 2.74944635778792e-41 postive

ATRX AC008750.5 0.639117055058178 6.52025796884971e-49 postive

TSC1 AC104825.1 0.411621696754498 2.32260465621343e-18 postive

GATA3 AC104825.1 0.415096244281721 1.12843795932309e-18 postive

IDH1 AC104825.1 0.439238783567007 5.90029309435373e-21 postive

TSC1 AC074117.1 0.432277470915003 2.80318469467168e-20 postive

GATA3 AC074117.1 0.436180458597977 1.17524372227711e-20 postive

TARDBP CEP83-DT 0.431322017927408 3.46200030348786e-20 postive

LEF1 AC106897.1 0.431641211699041 3.22650924434182e-20 postive

BACH2 LINC02352 0.416178921777795 8.99586858132049e-19 postive

TSC1 ZNF436-AS1 0.590361523805018 3.04698695356223e-40 postive

BRAF ZNF436-AS1 0.642516490275216 1.41451955220008e-49 postive

BRAF AC024581.1 0.507731583478538 1.63674520742934e-28 postive

ATRX ANKRD44-IT1 0.532194439941673 1.18486769259085e-31 postive

ATRX C1RL-AS1 0.51993424957195 4.77914843835621e-30 postive

TSC1 AC010186.3 0.451986687200065 3.09299308270608e-22 postive

MAPK8 AC010186.3 0.447197154488724 9.50190181736557e-22 postive

OTULIN AC010186.3 0.517484865570178 9.82609839451886e-30 postive

BRAF AC010186.3 0.739677020538558 7.18513899248226e-73 postive

ATRX AC010186.3 0.608194387042271 3.06697859271913e-43 postive

GATA3 AP001065.1 0.447901831833296 8.06451020466079e-22 postive

KLF9 AP003071.3 0.512665159803228 3.99001371380408e-29 postive

LEF1 AL163953.1 0.657499899576456 1.32584712865084e-52 postive

BRAF DLEU2 0.462183404349206 2.6709137829115e-23 postive

ATRX DLEU2 0.525765193088984 8.39159982452498e-31 postive

BRAF LINC01545 0.461461550585645 3.18525568375066e-23 postive

TSC1 AC009090.1 0.438942464272102 6.30958748298429e-21 postive

TSC1 AC027601.1 0.421432699553151 2.95998060668991e-19 postive

TSC1 AC005070.3 0.409583077329018 3.53362173876226e-18 postive

MAPK8 AC005070.3 0.431328943167412 3.45671578688246e-20 postive

OTULIN AC005070.3 0.452844729863488 2.52487145155974e-22 postive

BRAF AC005070.3 0.684440554217084 1.65312560042982e-58 postive

ATRX AC005070.3 0.645757595646365 3.23582792474628e-50 postive

TSC1 SEPT7-AS1 0.417530120018965 6.77164146826212e-19 postive

BRAF SEPT7-AS1 0.467322596023029 7.53094532810279e-24 postive

ATRX SEPT7-AS1 0.450359608061617 4.53765957058845e-22 postive

TSC1 AC015849.3 0.50386505398523 4.86936845957278e-28 postive

BRAF AC015849.3 0.516643172504784 1.25708369671711e-29 postive

ATRX AC015849.3 0.653142874826887 1.04903247734749e-51 postive

TSC1 AC068790.3 0.441516420922423 3.51586124128123e-21 postive

MAPK8 AC068790.3 0.481682664669872 1.95143235106291e-25 postive

IPMK AC068790.3 0.415236064076907 1.09593712561269e-18 postive

OTULIN AC068790.3 0.501431038917257 9.60410171964693e-28 postive

BRAF AC068790.3 0.712545414057754 2.1674041285236e-65 postive

ATRX AC068790.3 0.650271090861479 4.02530876794574e-51 postive

TSC1 LINC00456 0.500950162653775 1.09762942289799e-27 postive

IPMK LINC00456 0.406101298611441 7.18795282367991e-18 postive

BRAF LINC00456 0.657339051949366 1.43192907052228e-52 postive

ATRX LINC00456 0.475097586949618 1.06457977274966e-24 postive

AXL MIR4435-2HG 0.415724349199554 9.89498768190544e-19 postive

HSP90AA1 AL078644.1 0.420970332661406 3.26674448937555e-19 postive

MAPK8 AL731577.2 0.416731620160405 8.01042114589683e-19 postive

DNMT1 AL731577.2 0.423594811709475 1.86271471287085e-19 postive

SIRT1 AL731577.2 0.536083673729678 3.55193442768741e-32 postive

ATRX AL731577.2 0.540128874097307 9.97746433596389e-33 postive

TSC1 AC023794.1 0.414536187853605 1.26840195797649e-18 postive

MAPK8 AC023794.1 0.470202521922702 3.6699035157751e-24 postive

OTULIN AC023794.1 0.493043558260325 9.57235499831293e-27 postive

BRAF AC023794.1 0.810719976886557 7.68710551670706e-98 postive

ATRX AC023794.1 0.550429708288617 3.63563654466765e-34 postive

FLT3 LINC01781 0.586070565145576 1.50505205242372e-39 postive

BACH2 LINC01781 0.442250782834301 2.97286798893558e-21 postive

BRAF AC008781.1 0.493306054557707 8.9162849832703e-27 postive

GATA3 AC010761.4 0.419563764855673 4.40546309431588e-19 postive

TSC1 AL080317.3 0.517260639416125 1.04932600755736e-29 postive

MAPK8 AL080317.3 0.446247120614808 1.18463073345978e-21 postive

OTULIN AL080317.3 0.403761232273849 1.15304434314228e-17 postive

BRAF AL080317.3 0.791024143770045 5.94441335376413e-90 postive

ATRX AL080317.3 0.535215246013174 4.6545848191327e-32 postive

BRAF AL445231.1 0.575959975805947 5.91379679930866e-38 postive

TSC1 AC073130.2 0.409834514451269 3.35590707887775e-18 postive

MAPK8 AC073130.2 0.430765037559085 3.91412891138607e-20 postive

IPMK AC073130.2 0.419228943284987 4.72952760291418e-19 postive

OTULIN AC073130.2 0.51181666628798 5.09464970580689e-29 postive

BRAF AC073130.2 0.6840190451717 2.06825147185001e-58 postive

ATRX AC073130.2 0.641288232356378 2.46243287552977e-49 postive

BRAF AL357140.2 0.417416758203266 6.93528071508399e-19 postive

TSC1 THAP9-AS1 0.415185268962753 1.10763639794295e-18 postive

TNFRSF1B AC007384.1 0.407312846863614 5.61961345172146e-18 postive

BACH2 AC007384.1 0.441267404121547 3.72131596714884e-21 postive

TSC1 AL354993.2 0.503715090342901 5.0782681167156e-28 postive

MAPK8 AL354993.2 0.456345592576206 1.09655132460037e-22 postive

BRAF AL354993.2 0.747490837453975 3.36015693542236e-75 postive

SIRT1 AL354993.2 0.401320827132938 1.88004778924673e-17 postive

ATRX AL354993.2 0.572395615280039 2.09326113754439e-37 postive

MAPK8 AC234775.3 0.402058419688181 1.62248376035532e-17 postive

BRAF AC234775.3 0.691033349475848 4.7285363451239e-60 postive

ATRX AC234775.3 0.643668661976722 8.39002931653138e-50 postive

TSC1 AP005432.1 0.407139550935615 5.82134880757206e-18 postive

MAPK8 AP005432.1 0.440194783957324 4.75013892648195e-21 postive

OTULIN AP005432.1 0.470685130083446 3.25123331346125e-24 postive

BRAF AP005432.1 0.818102655174697 4.85874407975673e-101 postive

ATRX AP005432.1 0.498232418928403 2.32553718441269e-27 postive

TSC1 PCAT7 0.441655251667049 3.40621361827284e-21 postive

BRAF PCAT7 0.429586113838503 5.07158713184763e-20 postive

IDH1 PCAT7 0.470466872693911 3.43438944014172e-24 postive

TSC1 AC013356.2 0.428264596490471 6.77232711681998e-20 postive

BRAF AC013356.2 0.522826243550842 2.02519603189503e-30 postive

ATRX AC013356.2 0.454230057648096 1.81722412447669e-22 postive

TSC1 MAP3K14-AS1 0.413188157438287 1.67915304470789e-18 postive

ATRX MAP3K14-AS1 0.405195183540324 8.63515479628972e-18 postive

IDH1 MAP3K14-AS1 0.432156077086895 2.87948979355896e-20 postive

GATA3 LINC01833 0.404192804517766 1.05709890463228e-17 postive

KLF9 AL445426.1 0.48946300564289 2.50592951707659e-26 postive

OTULIN AC004637.1 0.437431694742858 8.87282585896892e-21 postive

BRAF AC004637.1 0.581435437866476 8.229322527247e-39 postive

ATRX AC004637.1 0.59480743887002 5.67698372942298e-41 postive

TSC1 AC008781.3 0.409378940719051 3.68468582318379e-18 postive

OTULIN AC008781.3 0.456654007319065 1.01840054156395e-22 postive

BRAF AC008781.3 0.767285179056254 1.65752890213278e-81 postive

ATRX AC008781.3 0.503213919746309 5.84274995508416e-28 postive

TSC1 AC006557.1 0.525149508387419 1.00997448014766e-30 postive

TSC1 SLC25A25-AS1 0.632246512740104 1.35003579820288e-47 postive

TSC1 AC009087.1 0.4832734074485 1.28811479687823e-25 postive

BRAF AC009087.1 0.583229745645047 4.27712179908036e-39 postive

ATRX AC009087.1 0.487176243357754 4.60608654447909e-26 postive

TSC1 AP000845.1 0.427644780408435 7.75271822175986e-20 postive

TSC1 AC022762.2 0.401092885062855 1.96748474794987e-17 postive

BRAF Z99127.1 0.507538268174127 1.72901228187048e-28 postive

ATRX Z99127.1 0.428905279834555 5.88732120141523e-20 postive

TSC1 AL590369.1 0.595541188370156 4.2912716980718e-41 postive

BRAF AL590369.1 0.53732492765852 2.41017477573834e-32 postive

ATRX AL590369.1 0.430905845768563 3.79461594863345e-20 postive

BNIP3 MEG3 0.533449146532512 8.04673514073869e-32 postive

ID1 AC005790.1 0.459865407178945 4.69483759340204e-23 postive

OTULIN OSMR-AS1 0.421018519554679 3.23336687649521e-19 postive

MAPK8 AC007684.1 0.453618777767754 2.10142271132856e-22 postive

OTULIN AC007684.1 0.471445968547013 2.68499480337682e-24 postive

BRAF AC007684.1 0.769463016499242 3.07066326918905e-82 postive

ATRX AC007684.1 0.595049057527518 5.17764879110678e-41 postive

TSC1 AP005899.1 0.486693120438746 5.23515947228658e-26 postive

MAPK8 AP005899.1 0.440590134765355 4.34186753510635e-21 postive

OTULIN AP005899.1 0.490521817091408 1.88750709752868e-26 postive

BRAF AP005899.1 0.576033751211511 5.76013899227527e-38 postive

ATRX AP005899.1 0.638935846018446 7.06979337559245e-49 postive

TNFRSF1B AC009093.6 0.423025771393368 2.10486947539474e-19 postive

FLT3 HSPC324 0.429308847327563 5.3894001864364e-20 postive

KLF9 HSPC324 0.478733942265661 4.19052792756151e-25 postive

ATRX AP005328.1 0.460185241831369 4.34442459306756e-23 postive

TSC1 AC024075.3 0.542210971046722 5.15542602729066e-33 postive

BRAF AC024075.3 0.618548951404161 4.54459770824076e-45 postive

SIRT1 AC024075.3 0.404992688312349 8.99580697629875e-18 postive

ATRX AC024075.3 0.6347405703041 4.53364765233574e-48 postive

TSC1 AC002451.1 0.404916825820049 9.13470090548046e-18 postive

BRAF AC002451.1 0.655163689356476 4.03650862909053e-52 postive

TSC1 AC034102.8 0.448805062971749 6.53178490284542e-22 postive

MAPK8 AC034102.8 0.44154911564837 3.48973013203417e-21 postive

OTULIN AC034102.8 0.434042780591009 1.89454144111275e-20 postive

BRAF AC034102.8 0.816195029962227 3.36492152232792e-100 postive

ATRX AC034102.8 0.502174618796676 7.80893085222438e-28 postive

TSC1 RORA-AS1 0.430683753134205 3.98480002241671e-20 postive

MAPK8 RORA-AS1 0.444747155520564 1.67564937048999e-21 postive

IPMK RORA-AS1 0.432643335178903 2.58505682102534e-20 postive

OTULIN RORA-AS1 0.452786512692866 2.55992554682293e-22 postive

BRAF RORA-AS1 0.633606248799365 7.45623740870294e-48 postive

ATRX RORA-AS1 0.649477700203702 5.82143796845844e-51 postive

TSC1 AC245060.6 0.560050131346691 1.48366137827802e-35 postive

BRAF AC245060.6 0.47684460033698 6.81164394732336e-25 postive

ATRX AC245060.6 0.430055506707371 4.57510329977771e-20 postive

TSC1 AC211433.1 0.403125825019981 1.31007726250911e-17 postive

MAPK8 AC211433.1 0.458048457549524 7.28330686148481e-23 postive

IPMK AC211433.1 0.410551203195564 2.89620184621369e-18 postive

OTULIN AC211433.1 0.51379047305065 2.88234698220507e-29 postive

BRAF AC211433.1 0.757608054170786 2.39162608011953e-78 postive

ATRX AC211433.1 0.609693590346944 1.68280558654234e-43 postive

DNMT1 TMPO-AS1 0.469784599375306 4.07514158707915e-24 postive

TARDBP TMPO-AS1 0.415698480902699 9.94873184142401e-19 postive

BRAF AC129507.1 0.549763488240207 4.51988016624434e-34 postive

ATRX AC129507.1 0.485073588554306 8.02901894667236e-26 postive

TSC1 AC007619.1 0.402845161436863 1.38596453497153e-17 postive

MAPK8 AC007619.1 0.439832646646787 5.15718095733408e-21 postive

OTULIN AC007619.1 0.464046219057262 1.69216417827779e-23 postive

BRAF AC007619.1 0.648983711219588 7.32070954753088e-51 postive

ATRX AC007619.1 0.634169098670893 5.8266326584011e-48 postive

BRAF AC141002.1 0.439650709146641 5.37446104870412e-21 postive

TSC1 AC007785.3 0.478198350575384 4.81073648369975e-25 postive

BRAF AC007785.3 0.567045750557794 1.35613324092788e-36 postive

ATRX AC007785.3 0.414454469745404 1.29020335016323e-18 postive

BRAF AC080013.3 0.429573992298227 5.08508719296786e-20 postive

ATRX AL133297.1 0.400480460958091 2.22269214722256e-17 postive

MAP3K7 AC027307.3 0.439532222467859 5.52078921161247e-21 postive

BRAF AC093503.1 0.579250749060426 1.81569669657397e-38 postive

AXL NKILA 0.494371310818579 6.68000509873964e-27 postive

BRAF AL139423.1 0.406827783570955 6.20236694276812e-18 postive

TSC1 AL353804.2 0.423491546772144 1.90452268169556e-19 postive

MAPK8 AL353804.2 0.421924597750381 2.66473891299655e-19 postive

IPMK AL353804.2 0.424117024945895 1.66474142094919e-19 postive

OTULIN AL353804.2 0.497300120714668 3.00404832030146e-27 postive

BRAF AL353804.2 0.658110741117321 9.89369123169407e-53 postive

ATRX AL353804.2 0.680854933496707 1.09837751847168e-57 postive

TSC1 AP001625.2 0.537671117513594 2.16247920759403e-32 postive

BRAF AP001625.2 0.716998348787861 1.47518430564827e-66 postive

ATRX AP001625.2 0.511701226296488 5.2666199115649e-29 postive

KLF9 ACTA2-AS1 0.475516024267913 9.56822160344897e-25 postive

TSC1 AC096642.1 0.468107706605141 6.19489004476963e-24 postive

BRAF AC007128.2 0.669645649487492 3.44822922290805e-55 postive

ATRX AC007128.2 0.498181677681051 2.35821478647194e-27 postive

OTULIN AC104564.5 0.411387435189638 2.43770334558398e-18 postive

BRAF AC104564.5 0.491466012377578 1.46476990273343e-26 postive

ATRX AC104564.5 0.550953874413844 3.0622757560315e-34 postive

CYLD AC026356.1 0.402385259068863 1.51975994586523e-17 postive

MAP3K7 LINC01719 0.554680721196911 8.95929202218503e-35 postive

TSC1 AC010132.4 0.475257724453303 1.0219945538607e-24 postive

MAPK8 AC010132.4 0.47972967297117 3.24001234022389e-25 postive

OTULIN AC010132.4 0.463375972853799 1.99481082646589e-23 postive

BRAF AC010132.4 0.792329138659547 1.89726685390657e-90 postive

ATRX AC010132.4 0.564182338387602 3.63566606347287e-36 postive

BRAF LINC00885 0.469980104660041 3.8803513653675e-24 postive

IDH1 LINC00885 0.516167502491334 1.44441608730908e-29 postive

BRAF AC007953.1 0.411225393202377 2.52058318423681e-18 postive

TSC1 TXNDC12-AS1 0.415572195723668 1.02152546164455e-18 postive

MAPK8 TXNDC12-AS1 0.433281793994158 2.24374387150681e-20 postive

IPMK TXNDC12-AS1 0.408278930201954 4.61480138016243e-18 postive

OTULIN TXNDC12-AS1 0.489618274846536 2.40406714456818e-26 postive

BRAF TXNDC12-AS1 0.703993021869414 3.28223154905456e-63 postive

ATRX TXNDC12-AS1 0.656141460729695 2.53609707012311e-52 postive

MAPK8 AC092171.1 0.417127803063962 7.37019959609822e-19 postive

IPMK AC092171.1 0.437548504978511 8.64252279262349e-21 postive

OTULIN AC092171.1 0.467829663989351 6.63889416102706e-24 postive

BRAF AC092171.1 0.629131049304439 5.20305202720831e-47 postive

ATRX AC092171.1 0.663830575928757 6.17042775336359e-54 postive

KLF9 LINC02489 0.4799589819673 3.05327607879773e-25 postive

ATRX SNHG16 0.401161664971993 1.94068844640974e-17 postive

BRAF AC090517.2 0.402001434631677 1.64107972111628e-17 postive

TSC1 AL731563.3 0.482097335605733 1.75153354661585e-25 postive

BRAF AL731563.3 0.522691823545756 2.10803637490797e-30 postive

ATRX AL731563.3 0.437468335785435 8.79994100348596e-21 postive

TSC1 SNHG4 0.408812457841227 4.13795875692989e-18 postive

ATRX SNHG4 0.527690617953399 4.68964624544924e-31 postive

OTULIN AC127024.3 0.404625034190691 9.68886040282374e-18 postive

BRAF AC127024.3 0.51335363491881 3.27063729181808e-29 postive

ATRX AC127024.3 0.571778712013703 2.60109038133523e-37 postive

TSC1 AL445309.1 0.412163061082714 2.07667964397069e-18 postive

MAPK8 AL445309.1 0.479967346540106 3.04666919464932e-25 postive

IPMK AL445309.1 0.40213357063457 1.59827615794585e-17 postive

OTULIN AL445309.1 0.488980523972811 2.85044243816505e-26 postive

BRAF AL445309.1 0.789366579134333 2.50609251225546e-89 postive

ATRX AL445309.1 0.59136229777953 2.09213430117867e-40 postive

BRAF AC006160.1 0.442700857921201 2.68185996898148e-21 postive

ATRX AC006160.1 0.60892370958279 2.29124767513946e-43 postive

PANX1 AC010503.4 -0.401069036877685 1.97686053529073e-17 negative

ID1 AC010503.4 0.440046695193865 4.9126153183663e-21 postive

GATA3 AC010503.4 0.457120332545624 9.10553663710619e-23 postive

TSC1 AP4B1-AS1 0.520057559068689 4.60812570221146e-30 postive

BRAF AP4B1-AS1 0.42886916195372 5.93403160934599e-20 postive

ATRX AP4B1-AS1 0.507618018486722 1.69034149588969e-28 postive

TSC1 AC024267.3 0.555354642410526 7.16198977944794e-35 postive

BRAF AC024267.3 0.681629451385561 7.31301236940455e-58 postive

ATRX AC024267.3 0.561371842163975 9.48230350368101e-36 postive

CD40 LINC01353 0.437253622673782 9.23559573837188e-21 postive

BRAF FARP1-AS1 0.463664861188851 1.85832136557695e-23 postive

TSC1 AC005828.1 0.401404208932019 1.84902693672488e-17 postive

MAPK8 AC005828.1 0.472868110695648 1.87519854584602e-24 postive

OTULIN AC005828.1 0.505343947390226 3.21426672535583e-28 postive

BRAF AC005828.1 0.821290369039535 1.81894330399037e-102 postive

ATRX AC005828.1 0.555849044118667 6.07513032380234e-35 postive

MAP3K7 CACNA1C-AS2 0.484393011468059 9.60324978302104e-26 postive

FLT3 LINC01215 0.44727096814579 9.34021407935798e-22 postive

BACH2 LINC01215 0.581270335784066 8.7383073235507e-39 postive

ATRX ARMCX5-GPRASP2 0.436279545351233 1.14942290018595e-20 postive

TSC1 AC022973.3 0.426661034601798 9.60279052743298e-20 postive

MAPK8 AC022973.3 0.441520205615931 3.51282650102241e-21 postive

IPMK AC022973.3 0.417681248128534 6.55938278763294e-19 postive

OTULIN AC022973.3 0.521082528002245 3.40191594299987e-30 postive

BRAF AC022973.3 0.73084280613042 2.46489995877996e-70 postive

ATRX AC022973.3 0.671089001602054 1.66816891513944e-55 postive

TSC1 AC008243.1 0.406921875225648 6.08486244623892e-18 postive

MAPK8 AC008243.1 0.453410765700036 2.20779310238448e-22 postive

IPMK AC008243.1 0.403674387560781 1.17336143858016e-17 postive

OTULIN AC008243.1 0.477203780794953 6.21216579055583e-25 postive

BRAF AC008243.1 0.6905960464379 6.0033904614935e-60 postive

ATRX AC008243.1 0.629768457683014 3.95299470350297e-47 postive

TSC1 AC025287.3 0.455502751315333 1.34157325499718e-22 postive

MAPK8 AC025287.3 0.448837397242041 6.48260563054129e-22 postive

OTULIN AC025287.3 0.402954885726198 1.35579556827468e-17 postive

BRAF AC025287.3 0.638082309686754 1.03422835305547e-48 postive

ATRX AC025287.3 0.55517463546898 7.60376257064344e-35 postive

TSC1 AL135999.1 0.476647245445309 7.16498153833463e-25 postive

TSC1 AP001462.1 0.403582587860723 1.19522043997136e-17 postive

TSC1 DCUN1D2-AS 0.446623318798128 1.08566184392856e-21 postive

BRAF DCUN1D2-AS 0.515761112019571 1.62614277670895e-29 postive

ATRX DCUN1D2-AS 0.621476593575698 1.34232299159636e-45 postive

TSC1 AC024267.5 0.440456516236298 4.47582753984337e-21 postive

MAPK8 AC024267.5 0.442017893097714 3.13544941904509e-21 postive

IPMK AC024267.5 0.426678138484563 9.56718325420527e-20 postive

OTULIN AC024267.5 0.503137259824984 5.96930888098939e-28 postive

BRAF AC024267.5 0.701355665399439 1.48863231706726e-62 postive

ATRX AC024267.5 0.696497951036013 2.30932382975575e-61 postive

BRAF AC020891.3 0.524564686486055 1.2038994641801e-30 postive

ATRX AC020891.3 0.563234643949843 5.02821975451671e-36 postive

TSC1 AC015923.1 0.405187126065617 8.64922995733639e-18 postive

MAPK8 AC015923.1 0.461540420390993 3.1246145195319e-23 postive

OTULIN AC015923.1 0.505824655674879 2.80709309238043e-28 postive

BRAF AC015923.1 0.826520679800053 7.18098242592316e-105 postive

ATRX AC015923.1 0.550216835628611 3.89774384857707e-34 postive

TSC1 NCBP2-AS1 0.606159558365637 6.89138487873629e-43 postive

MAPK8 NCBP2-AS1 0.408613838173337 4.30952733640682e-18 postive

BRAF NCBP2-AS1 0.716153061289191 2.4667183267301e-66 postive

ATRX NCBP2-AS1 0.644198679031083 6.5929979973238e-50 postive

TSC1 AC026771.1 0.416846181094784 7.81986997844771e-19 postive

MAPK8 AC026771.1 0.458773714671933 6.11423405703075e-23 postive

OTULIN AC026771.1 0.437334827714661 9.06838670648351e-21 postive

BRAF AC026771.1 0.672045214987725 1.02880889326279e-55 postive

ATRX AC026771.1 0.619999269908001 2.4878151200118e-45 postive

ATRX NOP53-AS1 0.410113887006502 3.16874838601427e-18 postive

TSC1 AC124312.4 0.425637298025307 1.19893781410174e-19 postive

MAPK8 AC124312.4 0.446622337721864 1.08590902851481e-21 postive

OTULIN AC124312.4 0.467662561781736 6.92069508365525e-24 postive

BRAF AC124312.4 0.795477679282468 1.16590217513795e-91 postive

ATRX AC124312.4 0.520327856441008 4.25412512125746e-30 postive

TSC1 AC018521.6 0.508602268609429 1.27797252964826e-28 postive

MAPK8 AC018521.6 0.458916305117557 5.90719388660601e-23 postive

BRAF AC018521.6 0.68550105694454 9.39242103773915e-59 postive

SIRT1 AC018521.6 0.401495341253964 1.81569801594513e-17 postive

ATRX AC018521.6 0.54428760536456 2.656204800739e-33 postive

TSC1 IGBP1-AS1 0.431532038493928 3.30522197540388e-20 postive

FASLG AC023449.2 0.406883553598496 6.13245301973656e-18 postive

TNFRSF1B AC023449.2 0.426616577596849 9.69595372326538e-20 postive

MAP3K7 MAGI2-AS3 0.445071970893307 1.55465554335351e-21 postive

FLT3 MAGI2-AS3 0.408756687529229 4.18544318684385e-18 postive

KLF9 MAGI2-AS3 0.533646764358541 7.56985317718002e-32 postive

BRAF AC063919.1 0.561258364181255 9.85463321917453e-36 postive

FLT3 U62631.1 0.430508506677776 4.14146772543057e-20 postive

BACH2 U62631.1 0.806465943371366 4.63488034694197e-96 postive

CD40 U62631.1 0.428996756211888 5.77063075373145e-20 postive

BRAF NPTN-IT1 0.489305721712964 2.61345830418816e-26 postive

ATRX NPTN-IT1 0.595778216426059 3.91976516716362e-41 postive

TSC1 RNASEH2B-AS1 0.447635532315682 8.58057628227597e-22 postive

MAPK8 RNASEH2B-AS1 0.44088931458759 4.05608491989682e-21 postive

OTULIN RNASEH2B-AS1 0.46813423119176 6.15409661704049e-24 postive

BRAF RNASEH2B-AS1 0.799768708234074 2.40378445235675e-93 postive

ATRX RNASEH2B-AS1 0.551308508671174 2.72609368727241e-34 postive

BRAF AC068768.2 0.636866110297319 1.77469260169702e-48 postive

ATRX AC068768.2 0.474551185149805 1.22349688284612e-24 postive

TNFRSF1B AC243960.3 0.487952712361958 3.74792272013574e-26 postive

FLT3 AC243960.3 0.420208692632117 3.84164674551819e-19 postive

BACH2 AC243960.3 0.517057068774392 1.11376438666493e-29 postive

TSC1 AC009088.1 0.451451615835875 3.50926600826241e-22 postive

BRAF AC009088.1 0.416415783569696 8.55974002685214e-19 postive

BRAF LINC00628 0.419362700208307 4.59734641049602e-19 postive

TSC1 AC016405.1 0.403617180866585 1.18693666987365e-17 postive

MAPK8 AC016405.1 0.471016821968335 2.99118821895133e-24 postive

OTULIN AC016405.1 0.513589532951566 3.05494252575748e-29 postive

BRAF AC016405.1 0.781806658501388 1.51039463124198e-86 postive

ATRX AC016405.1 0.60001626928064 7.6659340741271e-42 postive

CD40 AL117335.1 0.48787374703098 3.82743008385422e-26 postive

BCL2L11 AL117335.1 0.455022720000024 1.50448930821908e-22 postive

TSC1 Z98884.2 0.40838183085958 4.51880969862753e-18 postive

BRAF Z98884.2 0.545311790674746 1.91196341851495e-33 postive

ATRX Z98884.2 0.506890129504249 2.07749405074484e-28 postive

TSC1 LINC00894 0.555617047052972 6.56313646889822e-35 postive

BRAF LINC00894 0.635502283548645 3.24223874561868e-48 postive

ATRX LINC00894 0.526032091916015 7.74304521824965e-31 postive

TSC1 AL359644.1 0.468183347579687 6.07925685476116e-24 postive

MAPK8 AL359644.1 0.406420830794528 6.73680818228155e-18 postive

BRAF AL359644.1 0.719845657613587 2.57457780125165e-67 postive

ATRX AL359644.1 0.460402906169353 4.12083564373301e-23 postive

TSC1 AC073111.1 0.409929229688522 3.29126781231212e-18 postive

MAPK8 AC073111.1 0.460953306094638 3.60484576676915e-23 postive

BRAF AC073111.1 0.617450240717756 7.15855311464757e-45 postive

ATRX AC073111.1 0.506240584306353 2.49622398875106e-28 postive

TSC1 AC018616.1 0.42408979585401 1.67453171399277e-19 postive

MAPK8 AC018616.1 0.434300300437223 1.78895706647677e-20 postive

OTULIN AC018616.1 0.40008477518516 2.40460374770656e-17 postive

BRAF AC018616.1 0.77698252658607 7.8645298677268e-85 postive

ATRX AC018616.1 0.462280060515646 2.60858386492793e-23 postive

ATRX AC093620.1 0.445492576847609 1.41069888733609e-21 postive

BRAF AC093382.1 0.676742533488646 9.3250359072852e-57 postive

TNFRSF1B LINC02285 0.657525078219794 1.30996248252626e-52 postive

FLT3 LINC02285 0.474190979841999 1.34084125626587e-24 postive

TSC1 AL158212.3 0.561793562027358 8.21667302828707e-36 postive

MAPK8 AL158212.3 0.492455127410768 1.12213140799324e-26 postive

BRAF AL158212.3 0.516970431657697 1.14236126483148e-29 postive

SIRT1 AL158212.3 0.47363729630105 1.54320726371769e-24 postive

ATRX AL158212.3 0.545007179796302 2.1086124228405e-33 postive

TSC1 SSSCA1-AS1 0.414709643180345 1.22332043043003e-18 postive

BRAF CASC20 0.549020408479164 5.758787583435e-34 postive

TSC1 AL355388.2 0.45370696829881 2.05786556806148e-22 postive

BRAF AL355388.2 0.603430084635511 2.02285683981712e-42 postive

BRAF AP002907.1 0.554748735579286 8.75931102774011e-35 postive

ATRX AP002907.1 0.545940782768372 1.56152157445402e-33 postive

CD40 AL133346.1 0.558860489166513 2.21607866473476e-35 postive

BCL2L11 AL133346.1 0.491155807119098 1.5921560823595e-26 postive

TSC1 AL645608.1 0.470963044277919 3.03190993677698e-24 postive

BRAF AL645608.1 0.55606517654009 5.65289245559991e-35 postive

TSC1 NFYC-AS1 0.485315141841481 7.53395818168656e-26 postive

MAPK8 NFYC-AS1 0.401964643724768 1.65319686074493e-17 postive

BRAF NFYC-AS1 0.481412078659592 2.09385024746226e-25 postive

ATRX NFYC-AS1 0.448037773332055 7.81298916767298e-22 postive

TRIM11 AL136295.2 0.46110790493049 3.47175233772104e-23 postive

TSC1 AL355488.1 0.423390960372781 1.94613391920826e-19 postive

BRAF AL355488.1 0.459846785028237 4.71607777905857e-23 postive

BRAF AC072039.2 0.401439843017107 1.83592383330628e-17 postive

BRAF AC004908.2 0.52086488260215 3.62867499403239e-30 postive

ATRX AC004908.2 0.467686074069895 6.88034178801933e-24 postive

TSC1 AL160314.2 0.475629385169955 9.29531588025748e-25 postive

BRAF AL160314.2 0.649993725035648 4.58004543593404e-51 postive

TSC1 SSBP3-AS1 0.553277946847639 1.42551384719199e-34 postive

MAPK8 SSBP3-AS1 0.446835590417739 1.03346331589118e-21 postive

BRAF SSBP3-AS1 0.630535832444698 2.83722169922327e-47 postive

ATRX SSBP3-AS1 0.626215635431218 1.81359804470653e-46 postive

STAT3 AC008608.2 -0.418979171490437 4.98644686892203e-19 negative

LEF1 AC021242.3 0.435918586490081 1.24626737156722e-20 postive

BRAF AC069542.1 0.497975675725927 2.49560554697716e-27 postive

ATRX AC069542.1 0.546524571580303 1.2935086716777e-33 postive

TSC1 AP001178.1 0.448756527471234 6.60629688460672e-22 postive

MAPK8 AP001178.1 0.429960308508518 4.67176439480955e-20 postive

OTULIN AP001178.1 0.45251500029017 2.72985258463841e-22 postive

BRAF AP001178.1 0.631959055234768 1.52998352702188e-47 postive

ATRX AP001178.1 0.503810720936167 4.94405466659557e-28 postive

AXL GAS6-DT 0.591391066753155 2.06960439260976e-40 postive

TRIM11 AL390728.6 0.427634015744219 7.77092437764543e-20 postive

TSC1 AC091057.1 0.441830938626418 3.27227705452999e-21 postive

DNMT1 AC091057.1 0.472546687078674 2.03397561267763e-24 postive

ATRX AC091057.1 0.470544169526823 3.36838806919493e-24 postive

MAPK8 FO680682.1 0.425926274759319 1.1262082445647e-19 postive

IPMK FO680682.1 0.442124849987343 3.05972227054219e-21 postive

OTULIN FO680682.1 0.512290587558308 4.44495302737217e-29 postive

BRAF FO680682.1 0.626336226676151 1.72275857000764e-46 postive

ATRX FO680682.1 0.653925439833477 7.25338723214795e-52 postive

TNFRSF1B LINC02384 0.412843585980668 1.80361573113937e-18 postive

GATA3 AC024060.1 0.432593564921125 2.61371679145456e-20 postive

KLF9 ADAMTS9-AS1 0.572355865187502 2.12279322079005e-37 postive

TRIM11 AC009148.1 0.476508244726246 7.42464285866009e-25 postive

IPMK AC092756.1 0.408703984462504 4.23080842759777e-18 postive

OTULIN AC092756.1 0.445004914905939 1.5789070404114e-21 postive

BRAF AC092756.1 0.586978675497824 1.0754912735558e-39 postive

ATRX AC092756.1 0.565716085060124 2.14634910288711e-36 postive

TSC1 AC005954.1 0.426611302194796 9.70706764766217e-20 postive

BRAF AC005954.1 0.437471382167631 8.79390789453417e-21 postive

ATRX AC005954.1 0.50289116873457 6.39417287615882e-28 postive

TSC1 AC124069.1 0.402504594986381 1.48386976789027e-17 postive

MAPK8 AC124069.1 0.436036717371299 1.21371974536989e-20 postive

OTULIN AC124069.1 0.467900776853355 6.52243251782785e-24 postive

BRAF AC124069.1 0.807112595703419 2.50198743007123e-96 postive

ATRX AC124069.1 0.509648662868009 9.48345280293346e-29 postive

TSC1 AC009318.3 0.418209125071926 5.86810309019459e-19 postive

MAPK8 AC009318.3 0.436716201528248 1.04213693877894e-20 postive

OTULIN AC009318.3 0.432034607847005 2.9578892939413e-20 postive

BRAF AC009318.3 0.775648138623661 2.30626033731728e-84 postive

ATRX AC009318.3 0.503304053634929 5.69733501888157e-28 postive

TSC1 LINC01355 0.575531428384226 6.89007260867682e-38 postive

MAPK8 LINC01355 0.413771221800147 1.48751429578678e-18 postive

BRAF LINC01355 0.659088110019077 6.18469881768812e-53 postive

ATRX LINC01355 0.452929713124831 2.47454899133695e-22 postive

ATRX LRRC8C-DT 0.426315253953048 1.03512861441395e-19 postive

TSC1 AC137630.2 0.414561523189433 1.26171670969929e-18 postive

BRAF AC137630.2 0.532324162306632 1.13848666645121e-31 postive

GATA3 AC004982.2 0.511099991130608 6.25940663252454e-29 postive

TSC1 AC011466.3 0.501314060348812 9.9214265846631e-28 postive

BRAF AC011466.3 0.489832038486146 2.27049083921978e-26 postive

ATRX AC011466.3 0.507731370108327 1.63684431411973e-28 postive

BRAF AC107032.2 0.419007257809419 4.95688493677921e-19 postive

MYC AC006329.1 0.621908009560072 1.12030278226012e-45 postive

TSC1 LINC00630 0.456526044957755 1.05013534682018e-22 postive

MAPK8 LINC00630 0.516432295611747 1.33696779021178e-29 postive

IPMK LINC00630 0.496419291797611 3.82330705516439e-27 postive

OTULIN LINC00630 0.533561679179977 7.7716488762819e-32 postive

MAP3K7 LINC00630 0.418914985952966 5.05465754986812e-19 postive

BRAF LINC00630 0.708368184510258 2.57458346563247e-64 postive

SIRT1 LINC00630 0.445377209537897 1.44882219404364e-21 postive

ATRX LINC00630 0.761914973913033 9.80109369787041e-80 postive

TSC1 NR2F2-AS1 0.431278071097301 3.49572123198115e-20 postive

MAPK8 NR2F2-AS1 0.412187600576794 2.06616101662319e-18 postive

OTULIN NR2F2-AS1 0.412996566267037 1.74727569242221e-18 postive

BRAF NR2F2-AS1 0.753409564519641 5.05217873814359e-77 postive

ATRX NR2F2-AS1 0.529441183883257 2.7540784864328e-31 postive

STAT3 SNHG9 -0.453045878352542 2.40735880913662e-22 negative

RNF31 SNHG9 -0.460248180416672 4.27857094996828e-23 negative

BRAF AC009806.1 0.460331053382587 4.19335949157031e-23 postive

FASLG AC008750.1 0.410975091265486 2.65408274413776e-18 postive

BRAF AL109936.2 0.458623645644985 6.33986444354282e-23 postive

MAPK8 LINC02042 0.452293594279935 2.8766137633292e-22 postive

OTULIN LINC02042 0.480125553118345 2.92433241014532e-25 postive

BRAF LINC02042 0.813756107468776 3.86679620161272e-99 postive

ATRX LINC02042 0.503634689749797 5.1938881809491e-28 postive

LEF1 TGFB2-AS1 0.536043226285569 3.5970063968266e-32 postive

MAP3K7 ZFHX4-AS1 0.424772784675493 1.44526421587583e-19 postive

TSC1 AC138956.2 0.557647054940836 3.33118268478454e-35 postive

MAPK8 AC138956.2 0.442903792837356 2.56000989955728e-21 postive

OTULIN AC138956.2 0.433566243691117 2.10636144118483e-20 postive

BRAF AC138956.2 0.69691604595962 1.82786270655774e-61 postive

SIRT1 AC138956.2 0.40708279958367 5.88894891228427e-18 postive

ATRX AC138956.2 0.709174599791415 1.60217340948431e-64 postive

MAPK8 CASC2 0.414750816376107 1.21285312425432e-18 postive

SIRT1 CASC2 0.426681564877432 9.56006575427184e-20 postive

BRAF AC130324.1 0.40724405759882 5.69885513819783e-18 postive

TSC1 AP000692.1 0.458292580753286 6.86707244461059e-23 postive

BRAF AP000692.1 0.630532070151854 2.84184525536003e-47 postive

ATRX AP000692.1 0.588121955595763 7.03415745370371e-40 postive

TSC1 AP001107.1 0.494903572045799 5.78031387436579e-27 postive

BRAF AP001107.1 0.581078269951466 9.36975839971009e-39 postive

ATRX AP001107.1 0.616375415112003 1.11458318398543e-44 postive

BRAF AC084876.1 0.449982026010401 4.95828650346299e-22 postive

TSC1 AP006621.2 0.575986976143248 5.85709574446272e-38 postive

BRAF AP006621.2 0.540342327454303 9.32640613412829e-33 postive

ATRX AP006621.2 0.449732904650001 5.25662175824579e-22 postive

TSC1 AC107068.1 0.402239753208115 1.56467689553221e-17 postive

BRAF AC107068.1 0.451126850991287 3.78837512531816e-22 postive

DNMT1 CDKN2B-AS1 0.443675000726598 2.14473436844088e-21 postive

CDKN2A CDKN2B-AS1 0.511117118585739 6.22871787314341e-29 postive

MAPK8 AC011933.3 0.417823731481758 6.36526878486786e-19 postive

MAP3K7 AC011933.3 0.444022340453024 1.98010976540612e-21 postive

BRAF AC011933.3 0.586561918680734 1.25499499335602e-39 postive

ATRX AC011933.3 0.582653105204438 5.28058243527995e-39 postive

BRAF LINC01290 0.600169654577679 7.22299394349595e-42 postive

TSC1 AC096992.2 0.428297566242082 6.72374545065858e-20 postive

BRAF AC096992.2 0.423176046365593 2.03806313897964e-19 postive

ATRX AC096992.2 0.478693714935129 4.23423156872072e-25 postive

TSC1 AC006270.1 0.40662789586342 6.45944806460942e-18 postive

MAPK8 AC006270.1 0.458942757237315 5.86955330396876e-23 postive

OTULIN AC006270.1 0.488658403718161 3.10607893831332e-26 postive

BRAF AC006270.1 0.826015317680856 1.23575245275143e-104 postive

ATRX AC006270.1 0.529855027583883 2.42734103147308e-31 postive

TSC1 AC087301.1 0.4738119518811 1.47631640677178e-24 postive

BRAF AC087301.1 0.499108983738254 1.82672980073067e-27 postive

ATRX AC087301.1 0.583856678904291 3.39962553166368e-39 postive

TSC1 AL513327.3 0.537826648394802 2.05956430137072e-32 postive

MAPK8 AL513327.3 0.448453921073179 7.09012613465142e-22 postive

OTULIN AL513327.3 0.401065136174481 1.97839824988445e-17 postive

BRAF AL513327.3 0.683664762537065 2.49606277527695e-58 postive

ATRX AL513327.3 0.624220110363797 4.23073923628986e-46 postive

FLT3 LINC01082 0.403855826716442 1.13130771255398e-17 postive

TSC1 AC016737.1 0.408257987327586 4.63458232037326e-18 postive

BRAF AC016737.1 0.407185585199148 5.76707559144522e-18 postive

MLKL TTLL11-IT1 0.453528638786008 2.14688154474139e-22 postive

AXL TTLL11-IT1 0.403224807854215 1.28430052601896e-17 postive

DDX58 TTLL11-IT1 0.564623589124944 3.12505565401989e-36 postive

TSC1 AC112722.1 0.487711451678389 3.99610615998455e-26 postive

BRAF AC112722.1 0.68334588319668 2.95562240289696e-58 postive

ATRX AC112722.1 0.534223620551453 6.33196340157715e-32 postive

TSC1 ZFHX2-AS1 0.452329557270263 2.85225691065246e-22 postive

BRAF ZFHX2-AS1 0.421367393462192 3.00152378540707e-19 postive

TSC1 AC110792.3 0.425435272556254 1.25251061277434e-19 postive

MAPK8 AC110792.3 0.426627977901318 9.6719789722123e-20 postive

OTULIN AC110792.3 0.422728347956847 2.24351062211073e-19 postive

BRAF AC110792.3 0.722669487954178 4.4612287135844e-68 postive

ATRX AC110792.3 0.53562893525745 4.09251649997661e-32 postive

MAPK8 AC131532.1 0.415178445854977 1.10921724820726e-18 postive

BRAF AC131532.1 0.538114380397557 1.88178349716424e-32 postive

MAP3K7 MAGEA10-MAGEA5 0.500834747876726 1.13334559270913e-27 postive

TSC1 AL354726.1 0.436677428599885 1.05124988664791e-20 postive

MAPK8 AL354726.1 0.443343360196702 2.31447736320729e-21 postive

IPMK AL354726.1 0.422698975508464 2.25768050399811e-19 postive

OTULIN AL354726.1 0.506227227164605 2.50565686570523e-28 postive

BRAF AL354726.1 0.667338345605725 1.09154108384984e-54 postive

ATRX AL354726.1 0.679268871717684 2.5165652897628e-57 postive

OTULIN AC006059.1 0.441006055269321 3.94967167385224e-21 postive

BRAF AC006059.1 0.622248210889284 9.71245998335012e-46 postive

ATRX AC006059.1 0.63580883761532 2.83224740633444e-48 postive

MAP3K7 AC116614.1 0.478030091954614 5.02366999451191e-25 postive

TSC1 AC008764.2 0.494380026508919 6.6642138572516e-27 postive

ATRX AC008764.2 0.497209918828042 3.07925790473929e-27 postive

RNF31 AC008764.2 0.412551650685909 1.91613442702224e-18 postive

TSC1 AC007681.1 0.485557187060532 7.0681474635905e-26 postive

MAPK8 AC007681.1 0.438513630810474 6.95190059825849e-21 postive

BRAF AC007681.1 0.712634374501282 2.05514245505066e-65 postive

ATRX AC007681.1 0.554764039933372 8.71492458944807e-35 postive

TSC1 AC253576.2 0.480460585251023 2.68104653835534e-25 postive

MAPK8 AC253576.2 0.473041689381026 1.79460743477971e-24 postive

BRAF AC253576.2 0.684004723998975 2.08404082808522e-58 postive

ATRX AC253576.2 0.462912280928764 2.2348412875483e-23 postive

MAPK8 LIPC-AS1 0.424611425204867 1.49646983288288e-19 postive

OTULIN LIPC-AS1 0.456424413241796 1.07603332199625e-22 postive

BRAF LIPC-AS1 0.799941269533649 2.05232054945079e-93 postive

ATRX LIPC-AS1 0.47760065875859 5.61022190268416e-25 postive

TSC1 AC004233.1 0.413841003636273 1.46607280623277e-18 postive

BRAF AC004233.1 0.554990270040315 8.08418836631993e-35 postive

ATRX WWTR1-IT1 0.534480183276752 5.84789648646288e-32 postive

TSC1 AC023794.4 0.428527353331474 6.3945507659261e-20 postive

MAPK8 AC023794.4 0.483906172439944 1.09127134780259e-25 postive

OTULIN AC023794.4 0.497202929249293 3.08516281948884e-27 postive

BRAF AC023794.4 0.818210472381795 4.35238980494476e-101 postive

ATRX AC023794.4 0.572016493279986 2.39232061294747e-37 postive

TSC1 AC023908.3 0.463633860532114 1.87251508689721e-23 postive

MAPK8 AC023034.1 0.424375317696597 1.57463010252948e-19 postive

OTULIN AC023034.1 0.433154888751575 2.30784832102351e-20 postive

BRAF AC023034.1 0.66490988440217 3.63023841054126e-54 postive

ATRX AC023034.1 0.503515259268288 5.37045321822895e-28 postive

BRAF AC009955.4 0.504911270151373 3.63035947810064e-28 postive

DNMT1 AL513165.1 0.416305943217661 8.75933641701649e-19 postive

BRAF AC084880.3 0.532936761781276 9.42608406817457e-32 postive

ATRX AC084880.3 0.460814700551573 3.72844211390019e-23 postive

TSC1 NDUFA6-DT 0.405026770645552 8.93408491807211e-18 postive

BRAF NDUFA6-DT 0.480720046768697 2.50646788018344e-25 postive

ATRX NDUFA6-DT 0.485224985731169 7.71510278411293e-26 postive

MAPK8 AC024270.4 0.466172517489092 1.00160891316432e-23 postive

OTULIN AC024270.4 0.425257282350606 1.30165816687524e-19 postive

BRAF AC024270.4 0.655301699783702 3.78061199121555e-52 postive

ATRX AC024270.4 0.609330005105228 1.94706644003897e-43 postive

BRAF MAST4-AS1 0.441495444411065 3.53272807020258e-21 postive

TSC1 AC134407.1 0.434538479996856 1.69647373494857e-20 postive

MAPK8 AC134407.1 0.41655346399748 8.31586550852078e-19 postive

OTULIN AC134407.1 0.461354844601236 3.26915099113541e-23 postive

BRAF AC134407.1 0.725126650336164 9.53495499424545e-69 postive

ATRX AC134407.1 0.626634823869426 1.51678438394197e-46 postive

CFLAR MAP3K20-AS1 0.402655505351099 1.439674833769e-17 postive

ATRX MAP3K20-AS1 0.44819824083958 7.52602810175896e-22 postive

TSC1 AC068790.7 0.452722250144873 2.59917710927073e-22 postive

MAPK8 AC068790.7 0.469620880443278 4.24566150047558e-24 postive

OTULIN AC068790.7 0.469362732015964 4.52894184751589e-24 postive

BRAF AC068790.7 0.699517322230995 4.22881722654969e-62 postive

ATRX AC068790.7 0.629719535060731 4.0373378986711e-47 postive

TSC1 MIS18A-AS1 0.425646306698124 1.1966021989666e-19 postive

MAPK8 MIS18A-AS1 0.465351169560035 1.22704461504278e-23 postive

BRAF MIS18A-AS1 0.453189605692794 2.3267122627905e-22 postive

ATRX MIS18A-AS1 0.412100404886602 2.10377620945513e-18 postive

TSC1 AC007314.1 0.413297759841673 1.64136469616183e-18 postive

MAPK8 AC007314.1 0.41773759347586 6.48193332793263e-19 postive

OTULIN AC007314.1 0.43830696436369 7.28404447756459e-21 postive

BRAF AC007314.1 0.658853939663496 6.92268470528542e-53 postive

ATRX AC007314.1 0.603005624224048 2.38938410470217e-42 postive

ATRX AP003900.1 0.430092988436973 4.53758840473477e-20 postive

BRAF AC010595.1 0.462379561019833 2.54591880643975e-23 postive

FLT3 AL662844.4 0.419781427906237 4.20662084614566e-19 postive

BACH2 AL662844.4 0.507421292456616 1.78732051973379e-28 postive

IPMK AC099811.5 0.40722949461057 5.7157713634585e-18 postive

OTULIN AC099811.5 0.452110127805069 3.00409916828212e-22 postive

BRAF AC099811.5 0.571430162414966 2.94011911990488e-37 postive

ATRX AC099811.5 0.662055595026554 1.46928379065988e-53 postive

OTULIN AC099811.1 0.437995966312187 7.81354151818273e-21 postive

BRAF AC099811.1 0.548719649035882 6.35093120375241e-34 postive

ATRX AC099811.1 0.61881256551171 4.07412022106556e-45 postive

TSC1 AL359878.1 0.540187824908344 9.79329158397662e-33 postive

MAPK8 AL359878.1 0.462977530655923 2.19941671533307e-23 postive

OTULIN AL359878.1 0.458506227284384 6.52211342760271e-23 postive

BRAF AL359878.1 0.815366059184107 7.74734101389462e-100 postive

ATRX AL359878.1 0.539594964987598 1.18094884243773e-32 postive

BRAF NARF-AS1 0.459488065992071 5.14423106092597e-23 postive

ATRX NARF-AS1 0.462758035633258 2.32083576570463e-23 postive

BRAF AC005332.3 0.55086057500297 3.15733785205786e-34 postive

TSC1 SUGT1P4-STRA6LP 0.502592021766413 6.95104012337968e-28 postive

ATRX SUGT1P4-STRA6LP 0.483921653876814 1.08684788316368e-25 postive

GATA3 AC108471.2 0.428930862429014 5.85445509353668e-20 postive

TSC1 SNHG14 0.444468310983271 1.78688922351757e-21 postive

BRAF SNHG14 0.652757133266891 1.25777654377375e-51 postive

SIRT1 SNHG14 0.407587490952305 5.31389617116234e-18 postive

ATRX SNHG14 0.402778008615203 1.40475293088349e-17 postive

TSC1 AC092119.2 0.441514126620409 3.51770217522377e-21 postive

TSC1 AC025031.4 0.424968880381374 1.38535321831137e-19 postive

MAPK8 AC025031.4 0.463299772743268 2.03243151272352e-23 postive

OTULIN AC025031.4 0.496072667104884 4.20310538868655e-27 postive

BRAF AC025031.4 0.833573649607582 3.04815373786993e-108 postive

ATRX AC025031.4 0.543022729959904 3.98036121118415e-33 postive

BRAF AL133255.1 0.493295369329567 8.94210224419148e-27 postive

TSC1 OPA1-AS1 0.415622365983705 1.01085403019001e-18 postive

MAPK8 OPA1-AS1 0.467688033296627 6.87698973619178e-24 postive

IPMK OPA1-AS1 0.451237081403936 3.69127075711781e-22 postive

OTULIN OPA1-AS1 0.51049568195729 7.44333875810833e-29 postive

BRAF OPA1-AS1 0.731964361156317 1.18993035437221e-70 postive

ATRX OPA1-AS1 0.683801019246461 2.32201981475884e-58 postive

TSC1 AC015871.3 0.477491776337117 5.76938109668284e-25 postive

BRAF AC015871.3 0.41219823484498 2.06161903089396e-18 postive

ATRX AC015871.3 0.455073620548979 1.48632893171156e-22 postive

TSC1 SNRK-AS1 0.441184245602709 3.79252781248003e-21 postive

BRAF AC105429.1 0.550786371773386 3.23502319484974e-34 postive

ATRX AC105429.1 0.48947552144929 2.49756310471374e-26 postive

BRAF AL139260.1 0.508078397469874 1.48325040729156e-28 postive

TSC1 ZKSCAN2-DT 0.502066346439598 8.04804724695708e-28 postive

BRAF ZKSCAN2-DT 0.465279353568036 1.24898621442278e-23 postive

TSC1 AC100778.2 0.539275715579829 1.30600514908456e-32 postive

BRAF AC100778.2 0.436426722631542 1.11209866151935e-20 postive

ATRX AC100778.2 0.511187138596419 6.10479625111359e-29 postive

BRAF AC007966.1 0.485074679951633 8.02671145254426e-26 postive

DNMT1 AC016205.1 0.471374402719436 2.73381605305575e-24 postive

TSC1 AC022150.2 0.46676890359113 8.64039431096262e-24 postive

BRAF AC107027.3 0.460775859188612 3.76382105273882e-23 postive

SIRT1 AC107027.3 0.434261573288235 1.80445683073987e-20 postive

ATRX AC107027.3 0.440434054296102 4.49874332037616e-21 postive

TSC1 MIR2052HG 0.438976874546365 6.26065823094338e-21 postive

MAPK8 MIR2052HG 0.482353515311554 1.63828871520904e-25 postive

OTULIN MIR2052HG 0.436427738070056 1.11184533945863e-20 postive

BRAF MIR2052HG 0.795962514452589 7.554751302056e-92 postive

ATRX MIR2052HG 0.491074767286046 1.62719982965416e-26 postive

TSC1 SDCBP2-AS1 0.451143262132529 3.77376021442408e-22 postive

MAPK8 SDCBP2-AS1 0.413629111136443 1.53213827152548e-18 postive

OTULIN SDCBP2-AS1 0.434276654194982 1.7984053357371e-20 postive

BRAF SDCBP2-AS1 0.580627827105894 1.10335007178298e-38 postive

ATRX SDCBP2-AS1 0.655767187507316 3.03050767807241e-52 postive

BRAF AC012435.2 0.488220220493345 3.49051006875929e-26 postive

ATRX AC012435.2 0.47642428175547 7.58596804700485e-25 postive

TSC1 AC008870.2 0.420099270985388 3.93203277159279e-19 postive

TARDBP AL391244.1 0.400340099412257 2.28561779156428e-17 postive

BRAF AC115102.1 0.589267448818967 4.58906372943147e-40 postive

ATRX AC115102.1 0.553338729511024 1.39717753015561e-34 postive

TSC1 AC022167.2 0.463095060176117 2.13700114481674e-23 postive

BRAF AC022167.2 0.431162472404028 3.5859738699643e-20 postive

TSC1 AL139099.2 0.502093232092478 7.98800430253442e-28 postive

BRAF AL139099.2 0.592899472886915 1.17133077613241e-40 postive

ATRX AL139099.2 0.568313973250136 8.73522975542943e-37 postive

BRAF AL023755.1 0.484532218739066 9.25823054059297e-26 postive

ATRX AL023755.1 0.418989020636626 4.97606056100566e-19 postive

STAT3 SNHG7 -0.445313339231994 1.4703631216673e-21 negative

TSC1 AC067852.3 0.430927036323596 3.7769441024603e-20 postive

MAPK8 AC067852.3 0.400111724124975 2.39176304241269e-17 postive

IPMK AC067852.3 0.406528782288185 6.59077863446738e-18 postive

OTULIN AC067852.3 0.411544547330339 2.35990696948777e-18 postive

BRAF AC067852.3 0.529794720096157 2.47245205666322e-31 postive

ATRX AC067852.3 0.70349850716874 4.36355850525986e-63 postive

OTULIN AC027097.2 0.423434716925528 1.92792356834757e-19 postive

CYLD AC027097.2 0.400905949504905 2.0421651773302e-17 postive

BRAF AC027097.2 0.408573524817794 4.34519486040892e-18 postive

BACH2 AC027097.2 0.489610989773389 2.40875364641851e-26 postive

ATRX AC027097.2 0.445924543971463 1.27653727772188e-21 postive

TSC1 AC011405.1 0.432014560297396 2.97102910838612e-20 postive

MAPK8 AC011405.1 0.417582554693992 6.69724239750325e-19 postive

OTULIN AC011405.1 0.463794871308781 1.79994302732165e-23 postive

BRAF AC011405.1 0.698819500968306 6.27238089585211e-62 postive

ATRX AC011405.1 0.627082961733955 1.25260826013851e-46 postive

LEF1 LINC01124 0.45318804708884 2.32757235325453e-22 postive

ATRX LINC02256 0.410585978238526 2.87554671980901e-18 postive

TSC1 AC002347.1 0.423605615588648 1.85839320441999e-19 postive

BRAF AC002347.1 0.636566008047602 2.02687356331143e-48 postive

TSC1 AL365330.1 0.426002479511954 1.10776430861203e-19 postive
