# Supplementary material for: A five necroptosis-related lncRNA signature predicts the prognosis of bladder cancer and identifies hot or cold tumors
Source: Medicine (Baltimore). 2023 Oct 13;102(41):e35196. doi: 10.1097/MD.0000000000035196 (PMC10578762; doi:10.1097/MD.0000000000035196)

**Supplementary Figure 3:** ROC curves for a predictor that constructed by the predictive model, age, gender, and stage.


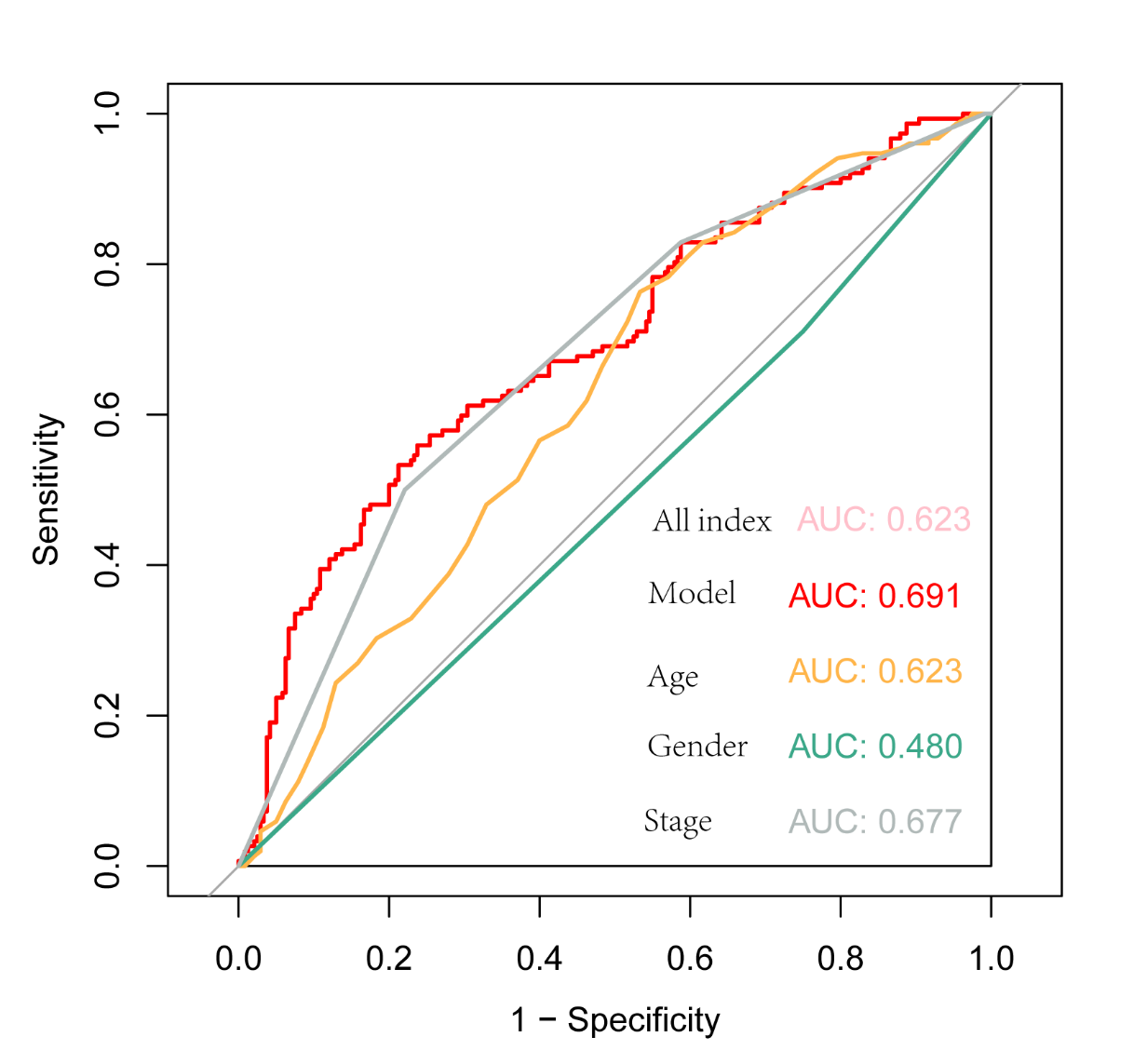

Supplement: Supplementary file 3 [file medi-102-e35196-s003.docx]
