# Supplementary figures and images for: A five necroptosis-related lncRNA signature predicts the prognosis of bladder cancer and identifies hot or cold tumors
Source: Medicine (Baltimore). 2023 Oct 13;102(41):e35196. doi: 10.1097/MD.0000000000035196 (PMC10578762; doi:10.1097/MD.0000000000035196)

A

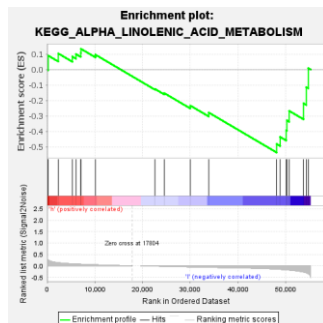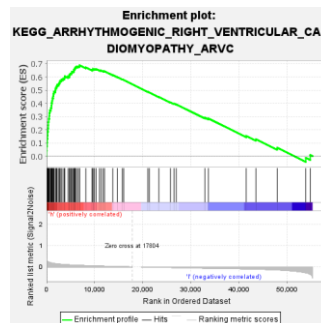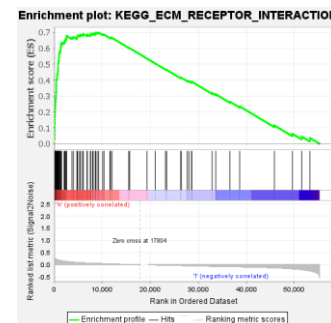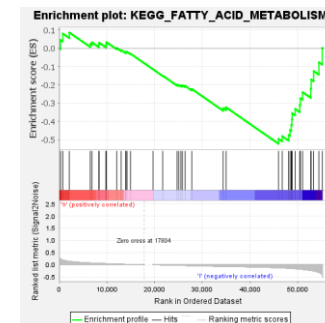

B

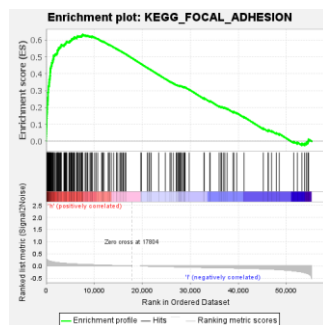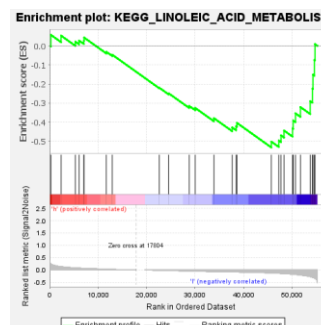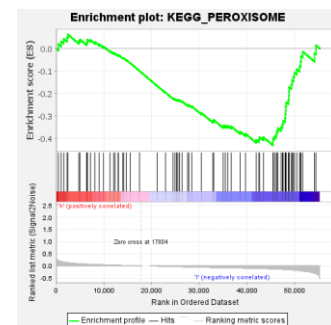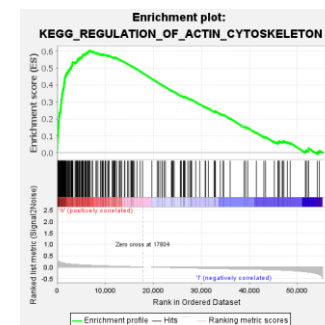

C

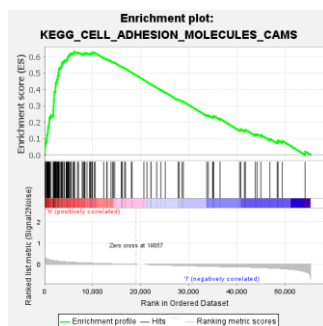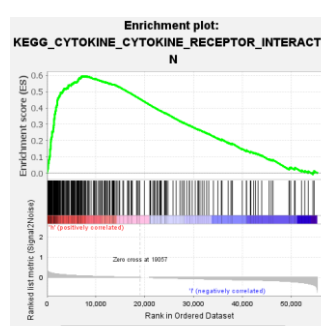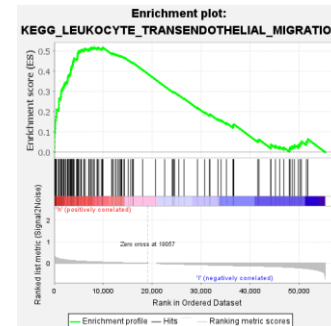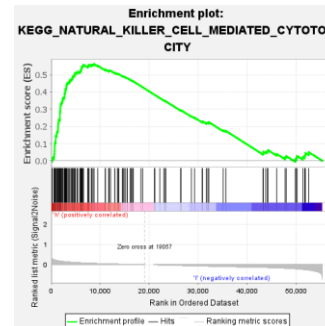

Supplement: Supplementary file 4 [file medi-102-e35196-s004.pdf]

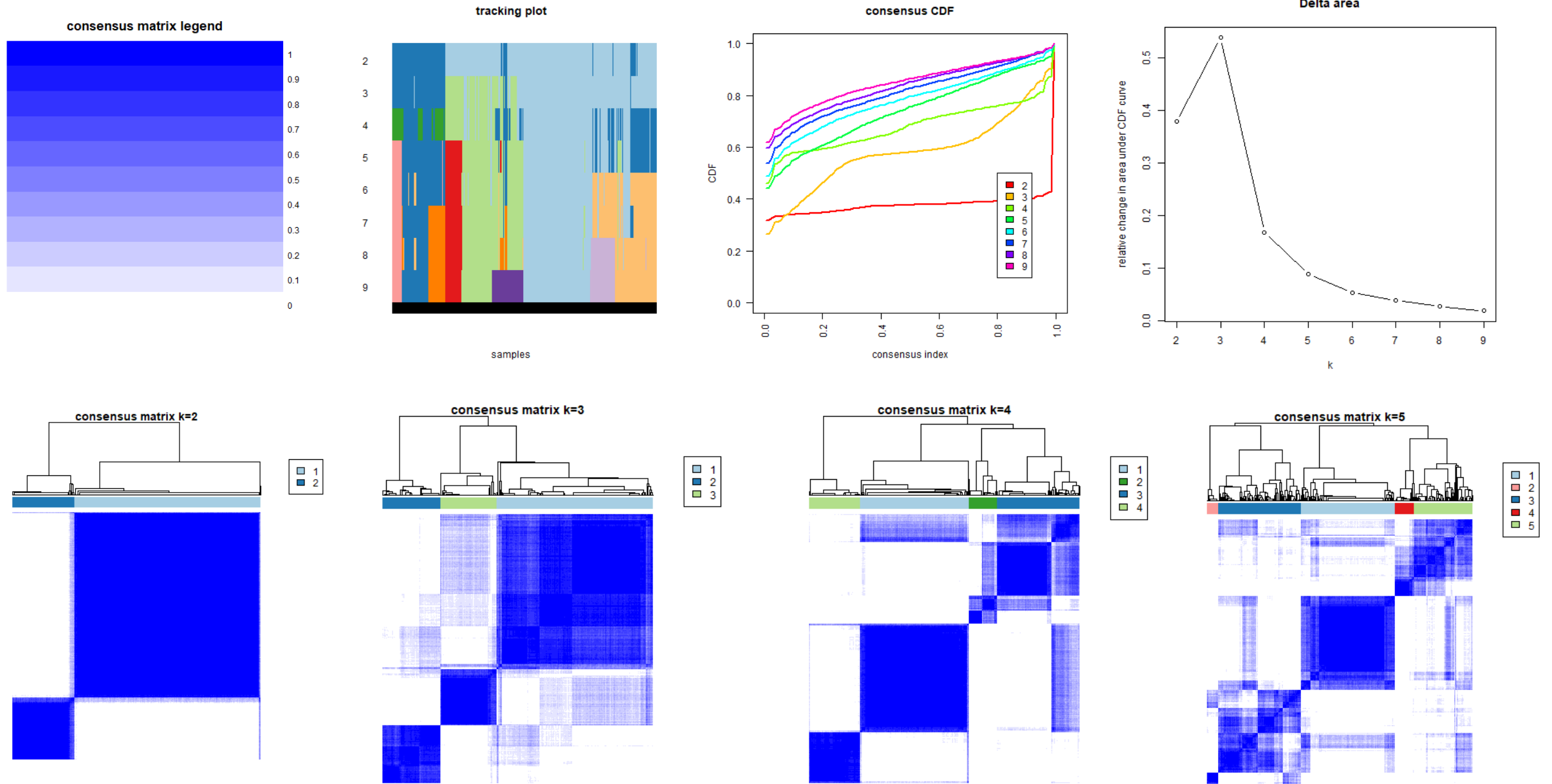

Supplement: Supplementary file 6 [file medi-102-e35196-s006.pdf]
