# Supplementary material for: A five necroptosis-related lncRNA signature predicts the prognosis of bladder cancer and identifies hot or cold tumors
Source: Medicine (Baltimore). 2023 Oct 13;102(41):e35196. doi: 10.1097/MD.0000000000035196 (PMC10578762; doi:10.1097/MD.0000000000035196)
Supplement: Supplementary file 5 [file medi-102-e35196-s005.docx]

| immune | cor | pvalue |
| --- | --- | --- |
| B cell_TIMER | -0.13344 | 0.007841 |
| T cell CD8+_TIMER | 0.340473 | 3.33E-12 |
| Neutrophil_TIMER | 0.176611 | 0.000414 |
| Macrophage_TIMER | 0.310137 | 2.82E-10 |
| Myeloid dendritic cell_TIMER | 0.265749 | 7.94E-08 |
| B cell naive_CIBERSORT | 0.165117 | 0.000974 |
| B cell memory_CIBERSORT | -0.11158 | 0.026397 |
| B cell plasma_CIBERSORT | -0.10786 | 0.031883 |
| T cell CD8+_CIBERSORT | -0.24687 | 6.55E-07 |
| T cell CD4+ memory resting_CIBERSORT | 0.137619 | 0.006088 |
| T cell follicular helper_CIBERSORT | -0.32341 | 4.30E-11 |
| T cell regulatory (Tregs)_CIBERSORT | -0.17545 | 0.000452 |
| NK cell activated_CIBERSORT | -0.15012 | 0.002746 |
| Macrophage M0_CIBERSORT | 0.228176 | 4.50E-06 |
| Macrophage M2_CIBERSORT | 0.242087 | 1.09E-06 |
| Myeloid dendritic cell activated_CIBERSORT | -0.26481 | 8.86E-08 |
| Neutrophil_CIBERSORT | 0.141387 | 0.00482 |
| B cell naive_CIBERSORT-ABS | 0.175165 | 0.000462 |
| T cell CD4+ memory resting_CIBERSORT-ABS | 0.186158 | 0.000195 |
| T cell follicular helper_CIBERSORT-ABS | -0.12078 | 0.016187 |
| Macrophage M0_CIBERSORT-ABS | 0.242992 | 9.89E-07 |
| Macrophage M1_CIBERSORT-ABS | 0.101137 | 0.044284 |
| Macrophage M2_CIBERSORT-ABS | 0.23636 | 1.97E-06 |
| Myeloid dendritic cell activated_CIBERSORT-ABS | -0.24118 | 1.20E-06 |
| Mast cell resting_CIBERSORT-ABS | 0.107727 | 0.032096 |
| Neutrophil_CIBERSORT-ABS | 0.147157 | 0.003334 |
| Macrophage M1_QUANTISEQ | 0.240672 | 1.26E-06 |
| Macrophage M2_QUANTISEQ | 0.115861 | 0.021106 |
| Monocyte_QUANTISEQ | 0.12282 | 0.01446 |
| NK cell_QUANTISEQ | -0.11317 | 0.024305 |
| Myeloid dendritic cell_QUANTISEQ | -0.11341 | 0.024008 |
| uncharacterized cell_QUANTISEQ | -0.13605 | 0.006699 |
| T cell_MCPCOUNTER | -0.1308 | 0.009164 |
| Monocyte_MCPCOUNTER | 0.184381 | 0.000225 |
| Macrophage/Monocyte_MCPCOUNTER | 0.184381 | 0.000225 |
| Myeloid dendritic cell_MCPCOUNTER | 0.145814 | 0.003637 |
| Endothelial cell_MCPCOUNTER | 0.196371 | 8.36E-05 |
| Cancer associated fibroblast_MCPCOUNTER | 0.354071 | 3.87E-13 |
| Myeloid dendritic cell activated_XCELL | 0.135634 | 0.006871 |
| T cell CD4+ central memory_XCELL | -0.25794 | 1.94E-07 |
| T cell CD4+ effector memory_XCELL | -0.12098 | 0.016011 |
| T cell CD8+ naive_XCELL | -0.22395 | 6.80E-06 |
| T cell CD8+_XCELL | -0.26609 | 7.63E-08 |
| Class-switched memory B cell_XCELL | -0.11995 | 0.016941 |
| Common myeloid progenitor_XCELL | 0.099271 | 0.04837 |
| Myeloid dendritic cell_XCELL | 0.127796 | 0.010912 |
| Endothelial cell_XCELL | 0.243343 | 9.53E-07 |
| Eosinophil_XCELL | -0.13362 | 0.007755 |
| Cancer associated fibroblast_XCELL | 0.281033 | 1.27E-08 |
| Granulocyte-monocyte progenitor_XCELL | 0.187785 | 0.000171 |
| Hematopoietic stem cell_XCELL | 0.221671 | 8.48E-06 |
| Macrophage_XCELL | 0.21026 | 2.46E-05 |
| Macrophage M1_XCELL | 0.22549 | 5.86E-06 |
| Macrophage M2_XCELL | 0.208073 | 3.00E-05 |
| Monocyte_XCELL | 0.259544 | 1.62E-07 |
| NK cell_XCELL | -0.12108 | 0.015924 |
| B cell plasma_XCELL | -0.20122 | 5.51E-05 |
| T cell CD4+ Th2_XCELL | 0.247254 | 6.28E-07 |
| T cell regulatory (Tregs)_XCELL | -0.11363 | 0.02373 |
| immune score_XCELL | 0.11219 | 0.025578 |
| stroma score_XCELL | 0.2817 | 1.17E-08 |
| microenvironment score_XCELL | 0.200007 | 6.12E-05 |
| Cancer associated fibroblast_EPIC | 0.320874 | 6.20E-11 |
| T cell CD4+_EPIC | -0.31655 | 1.15E-10 |
| T cell CD8+_EPIC | -0.11547 | 0.021552 |
| Endothelial cell_EPIC | 0.124113 | 0.013452 |
| Macrophage_EPIC | 0.199865 | 6.20E-05 |
| NK cell_EPIC | 0.143418 | 0.00424 |
| uncharacterized cell_EPIC | -0.14662 | 0.003452 |
