# Supplementary material for: A five necroptosis-related lncRNA signature predicts the prognosis of bladder cancer and identifies hot or cold tumors
Source: Medicine (Baltimore). 2023 Oct 13;102(41):e35196. doi: 10.1097/MD.0000000000035196 (PMC10578762; doi:10.1097/MD.0000000000035196)
Supplement: Supplementary file 7 [file medi-102-e35196-s007.docx]

immune pvalue

T cell CD8+_TIMER 2.8165052068841e-10

Neutrophil_TIMER 3.52434023285204e-06

Macrophage_TIMER 0.0350993889606209

Myeloid dendritic cell_TIMER 4.61748794386209e-09

B cell plasma_CIBERSORT 0.000264967118835318

T cell CD4+ memory activated_CIBERSORT 0.00652164375643246

T cell follicular helper_CIBERSORT 0.000183782228536571

T cell regulatory (Tregs)_CIBERSORT 0.00686631491584446

Macrophage M1_CIBERSORT 0.00223933587216566

Macrophage M2_CIBERSORT 0.00378891572245255

Myeloid dendritic cell activated_CIBERSORT 0.000254943330313975

Mast cell resting_CIBERSORT 0.0275709209737392

Eosinophil_CIBERSORT 0.022535308428755

Neutrophil_CIBERSORT 0.024944280919752

B cell naive_CIBERSORT-ABS 0.0410517411956861

T cell CD8+_CIBERSORT-ABS 0.00989712978073916

T cell CD4+ memory activated_CIBERSORT-ABS 0.00436964395232137

NK cell activated_CIBERSORT-ABS 0.0100614101257362

Macrophage M1_CIBERSORT-ABS 0.000399243690042013

Macrophage M2_CIBERSORT-ABS 5.68560169558903e-06

Myeloid dendritic cell activated_CIBERSORT-ABS 0.00601069119813751

Mast cell resting_CIBERSORT-ABS 0.00391588843651848

Neutrophil_CIBERSORT-ABS 0.012344998406407

Macrophage M1_QUANTISEQ 6.37948878477201e-07

Monocyte_QUANTISEQ 0.0386060356020269

NK cell_QUANTISEQ 0.00747955487255943

T cell CD8+_QUANTISEQ 0.00594998977279846

T cell regulatory (Tregs)_QUANTISEQ 0.0404315018932506

Myeloid dendritic cell_QUANTISEQ 0.000245606821668058

uncharacterized cell_QUANTISEQ 0.00375843198371238

T cell CD8+_MCPCOUNTER 0.0201861901578409

cytotoxicity score_MCPCOUNTER 1.48147459269891e-05

NK cell_MCPCOUNTER 0.00866454443204292

Monocyte_MCPCOUNTER 0.000230937643411098

Macrophage/Monocyte_MCPCOUNTER 0.000230937643411098

Myeloid dendritic cell_MCPCOUNTER 0.000853757879773593

Neutrophil_MCPCOUNTER 0.0185070747735473

Cancer associated fibroblast_MCPCOUNTER 4.81901131312907e-09

Myeloid dendritic cell activated_XCELL 4.08067219334564e-07

T cell CD4+ central memory_XCELL 1.63621230051328e-06

T cell CD8+ central memory_XCELL 0.0252346093140727

T cell CD8+ effector memory_XCELL 0.0446414512108755

Myeloid dendritic cell_XCELL 7.53831686391773e-06

Endothelial cell_XCELL 0.00499797893123833

Cancer associated fibroblast_XCELL 1.90764434904009e-05

Granulocyte-monocyte progenitor_XCELL 0.00259996844172046

Macrophage_XCELL 2.04109834317595e-06

Macrophage M1_XCELL 1.09925517936656e-07

Macrophage M2_XCELL 0.000513378771508895

B cell memory_XCELL 0.00343894769503768

Monocyte_XCELL 3.28272693380918e-07

B cell naive_XCELL 0.0374075395472133

Plasmacytoid dendritic cell_XCELL 7.2495703336644e-05

T cell CD4+ Th1_XCELL 0.000581259315242431

T cell CD4+ Th2_XCELL 1.39977138074173e-07

immune score_XCELL 9.28231771238064e-05

stroma score_XCELL 0.000103488210461064

microenvironment score_XCELL 6.39426901109907e-06

Cancer associated fibroblast_EPIC 1.7637856746592e-07

T cell CD4+_EPIC 0.000178668076179633

Macrophage_EPIC 4.74455008232811e-06

NK cell_EPIC 3.42208959641125e-06

uncharacterized cell_EPIC 0.0170922597976287
